# Nuclear RhoA Activation Regulates Nucleus Size and DNA Content via Nuclear Activation of ROCK and pErk

**DOI:** 10.3390/cells14060404

**Published:** 2025-03-10

**Authors:** Lap P. Nguyen, Julius Svensmark, Xin Jiang, Alexander Jordan, Cord Brakebusch

**Affiliations:** Biotech Research and Innovation Center (BRIC), University of Copenhagen, Ole Maaløes Vej 5, 2200 Copenhagen, Denmark; nphuoclap27@gmail.com (L.P.N.); juliussvensmark@gmail.com (J.S.); xin.jiang@bric.ku.dk (X.J.); alexandersvorre@gmail.com (A.J.)

**Keywords:** RhoA, nuclear F-actin, ROCK

## Abstract

RhoA is a major regulator of the actin cytoskeleton. Its function in the nucleus, however, is unclear. Fusing wildtype, fast cycling, constitutively active, and dominant negative forms of RhoA with tags promoting nuclear or cytoplasmic location and allowing specific detection, we established a platform to distinguish the functions of nuclear and cytoplasmic RhoA. Our data show that nuclear but not cytoplasmic activation of RhoA regulates DNA amount and nuclear size. This is mediated by sequential nuclear activation of the RhoA effector ROCK and Erk, a major cell cycle regulating kinase. The inhibition of ROCK or Erk activation in untransfected cells reduced DNA amounts to a similar extent, suggesting that endogenous activation levels of nuclear RhoA-ROCK-Erk signaling are sufficient for regulation. We reveal, furthermore, that GDP-bound, but not activated RhoA, translocates to the nucleus, indicating relatively separated cytoplasmic and nuclear RhoA signaling. Moreover, even the massive nuclear activation of RhoA does not cause an obvious increase in nuclear F-actin, indicating that RhoA activation is not critical for nuclear F-actin formation.

## 1. Introduction

RhoA is a ubiquitously expressed small GTPase of about 21 kD which is present in an active GTP-bound and an inactive GDP-bound form [1,2,3]. The activation of RhoA is mediated by guanine nucleotide exchange factors (GEFs), while inactivation is promoted by GTPase-activating proteins (GAPs). The binding of RhoA to cytosolic guanine nucleotide dissociation inhibitors (GDIs) is preventing the biological function of RhoA, but also its degradation [4]. Only in its active form can RhoA combines with different types of effector molecules that mediate its biological effects. By competing for GDI1 or interacting with GEFs, crosstalk can occur between RhoA and the small GTPases Rac1 and Cdc42, which regulate in particular branched actin polymerization and cell polarity [4,5]. As a master regulator of the actin cytoskeleton, RhoA is crucial for cell migration and the regulation of cell shape and cell adhesion. In addition, it is important for mechanosignaling [1,6]. The inactivation of the GTPase function results in constitutively active (CA) mutant forms of RhoA (G14V, Q63L) [7]. The fast-cycling (FC) mutation F30L confers enhanced intrinsic GDP by the GTP exchange rate but with normal GTPase activity [8]. Dominant negative (DN) mutants of RhoA strongly bind to GEFs without exchanging GDP to GTP and thus prevent the GEF-dependent activation of endogenous RhoA, RhoB, and RhoC [9]. In cancer, interestingly, dominant negative mutations of RhoA have been found, but constitutively active or fast-cycling mutants have not been found [10].

While there is plenty of information about the cytoplasmic effects of RhoA signaling, relatively little is known about its presence and role in the nucleus. Because of its small size, RhoA is expected to diffuse relatively freely in and out of the nucleus [11]. Cell fractionation experiments and studies with GFP-fusion proteins indicated the presence of RhoA in the nucleus of mammalian cells to a low (5%) [12] or medium (20%) [13] extent. RhoA prenylation was reported by one study to be essential for nuclear localization [12], while another found that only non-prenylated RhoA will translocate to the nucleus [14]. In chondrocytes, the strong nuclear localization of RhoA was shown by immunostaining [15]. Functionally, nuclear RhoA was correlated in these cells with dedifferentiation. Immunostaining also indicated the strong nuclear localization of RhoA in several cancer cell lines [8,16,17]. Here, nucleolar staining was noticed. Irradiation led to the activation of RhoA in isolated nuclei mediated by the GEF Net1, but the consequences of this activation were not investigated [12].

Several studies indicated the nuclear presence of RhoA effectors such as mDia1, mDia2, ROCK1, and ROCK2 [18,19,20,21]. Of these, mDia1 and mDia2 were found to mediate serum-induced nuclear actin polymerization in NIH 3T3 cells [21], and mDia2-dependent nuclear F-actin formation was reported to promote centromere movement [22], potentially downstream of Rho signaling, and to initiate DNA replication [23]. Moreover, a database analysis predicted the nuclear localization of the RhoA effectors RHPN, RHPN2, RTKN, RTKN2, PKN1, PKN2, PKN3, and CIT [24].

Although these data suggest an important function of RhoA in nuclear actin polymerization, this has not been studied directly up to now. Recent data demonstrate that nuclear actin polymerization is important for DNA repair, transcription, and replication [25], which further increased interest in this topic. Yet, since cytoplasmic stress fibers are connected to the nuclear membrane via the LINC complex, RhoA might also affect the nuclear shape and intranuclear functions by cytoplasmic activation [6]. Finally, it should be noted that it is unclear if and how cytoplasmic and nuclear RhoA activation are connected, and whether cytoplasmic RhoA activation will trigger nuclear RhoA signaling. A major obstacle in addressing these questions is the difficulty in distinguishing nuclear effects that are dependent on nuclear RhoA activation from the effects of cytoplasmic RhoA activation that, via downstream effectors, regulate nuclear functions.

Here, we report the establishment of a novel platform to investigate the nuclear and cellular functions of RhoA by tagging wildtype and mutant forms of RhoA with nuclear localization or nuclear export sequences. We reveal that activated RhoA is not able to translocate efficiently to the nucleus and the nuclear activation of a RhoA-ROCK-Erk cascade also controls DNA amounts under normal growth conditions.

## 2. Materials and Methods

### 2.1. Cell Culture

Fibroblast NIH 3T3 cells (ATCC CRL-1658, Manassas, VA, USA) were cultured in Dulbecco’s modified Eagle’s medium (DMEM, Cytiva, Freiburg, Germany) supplemented with 10% fetal bovine serum (Cytiva HyClone, SV30160.03, Cytiva, Freiburg, Germany) and 1% penicillin–streptomycin (Gibco, 15140-122, Thermo Fisher Scientific, Roskilde, Denmark) in a 5% CO_2_ incubator at 37 °C.

### 2.2. Plasmids

cDNA of human RhoA was inserted into the pcDNA3.1+ vector (Thermo Fisher Scientific, Roskilde, Denmark) by restricted enzyme digestion. PCR mutagenesis was performed on the cDNA of human RhoA, Rac1, and Cdc42 to generate CA (Q63L), FC (F30L), and DN (T19N) forms of RhoA, and CA (Q61L), FC (F28L), and DN (T17N) forms of Rac1 and Cdc42, respectively. After confirming the mutant forms of RhoA by DNA sequencing, a second PCR was conducted to add HA-, NES-, or NLS-tags to the N-terminus of RhoA using the primer listed in Table S1. Both the forward and reverse primer contained a sequence for restriction enzyme digestion which was used to clone the RhoA cDNAs to the pcDNA3.1+ expression vector by restriction enzyme digestion. The NLS was from the SV40 large T-antigen with the amino acid sequence PPKKKRKV, the NES used was from HIV-1 Rev protein with the amino acid sequence LPPLERLTL, and the HA tag had the amino acid sequence YPYDVPDYA. DNA sequencing by Eurofins Genomics (Ebersberg, Germany) was performed to confirm the correct cloning. pcDNA3-GFP was used as a control for transfected cells.

### 2.3. DNA Transfection

Additionally, 3 × 10^5^ NIH 3T3 cells were seeded on a 6-well plate overnight. Cells were transfected with 2.5 µg of DNA plasmids per well using Lipofectamine™ LTX Plus (Thermo Fisher Scientific, Roskilde, Denmark; #15338100) following the instructions of the manufacturer. Twenty-four hours after transfection, cells were re-seeded into 8-well plastic chamber slides (Thermo Fisher Scientific, Roskilde, Denmark; #177445) at 0.3 × 10^5^ cells per well and cultured for twenty-four hours before fixation.

### 2.4. Inhibitor Treatment

Twelve hours after transfection, cells were re-seeded in 8-well chamber slides with 0.3 × 10^5^ cells per well and cultured for twelve hours. Cells were then treated with or without 10 µM Y27632 (Tocris Bioscience, Abingdon, UK; # 1254) for 24 h or for 3 h with 10 µM U0126 (Sigma-Aldrich, Søborg, Denmark; # 109511-58-2) before fixation.

### 2.5. Immunofluorescence Staining

Cells seeded in 8-well chamber slides were fixed with 4% paraformaldehyde in a phosphate-buffered saline (PBS) for 15 min at room temperature. Then, cells were permeabilized with 0.1% Triton X-100 in PBS for 10 min at room temperature. Fixed and permeabilized cells were washed three times with PBS and blocked with 1% bovine serum albumin in PBS for 1 h at room temperature. Cells were then incubated with a rabbit anti-HA tag antibody (Cell Signaling Technology, Herlev, Denmark; #3724) overnight at 4 °C. After three washes with PBS, cells were incubated for 1 h at room temperature with goat anti-rabbit IgG coupled with Alexa-488 (Thermo Fisher Scientific, Roskilde, Denmark; # A1034) for anti-HA-tag detection, with Alexa Fluor Plus 647 Phalloidin (Thermo Fisher Scientific, Roskilde, Denmark; # A30107) for F-actin staining, and with 4′,6′-diamidino-2-phenylindole (DAPI) (Thermo Fisher Scientific, Roskilde, Denmark; # D1306) to label nuclei. After the last wash, the walls of the wells were removed, leaving behind only the slide with the stained cells. Slides were mounted with 5 μL of mounting media (Agilent Dako; Glostrup, Denmark; # S3023) and stored at 4 °C. For the detection of nuclear pErk, cells were stained with rabbit anti-phospho-p44/42 (Erk1/2) (Cell Signaling Technology, Herlev, Denmark; #4370) and mouse anti-HA tag (Cell Signaling Technology, Herlev, Denmark; #6E2) antibodies. As secondary antibodies, goat-anti-rabbit IgG coupled with Alexa-488 and goat-anti-mouse IgG coupled with Alexa 647 (Thermo Fisher Scientific, Roskilde, Denmark; # A-21235) were used.

### 2.6. Imaging Acquisition

Images were taken using an EVOS M7000 microscope (Thermo Fisher Scientific, Roskilde, Denmark). All images were taken with a 40× magnification objective, and each image included both the transfected and untransfected cells. For 3D Z-stack acquisition, a confocal microscope (Zeiss LSM800; Carl Zeiss Microscopy, Oberkochen, Germany) was used to capture 15 images at a step distance of 0.4 μm with a pinhole setting of 0.65 AU/28 µm for phalloidin and HA and 0.73 AU/28 µm for DAPI. The pictures were taken at 63x magnification using the oil immersion objective. All images of the experiment were captured with the same exposure time of the camera for the respective fluorescent channels acquired with ZEN 3.6 software (Carl Zeiss Microscopy). Images from the experiments involving both ROCK and Erk inhibitors were acquired by a high-throughput content screening inverted widefield microscope system (ScanR, Olympus, Søborg, Denmark) and analyzed using CellProfiler software (latest v. 4.2.8). Movies of the 3D image, reconstructed by ZEN 3.6 software (Carl Zeiss Microscopy, Oberkochen, Germany), were made from a sequence of screenshots using the Window Screen Recorder.

### 2.7. CellProfiler Analysis

Images were analyzed using CellProfiler version 4.0.7 (www.cellprofiler.org) [26,27]. The analysis pipeline and module settings used for the quantification of image sets are shown in Table S2. Nuclei were identified by DAPI staining and cells by phalloidin staining. Integrated intensity is the sum of the intensities of each pixel of an object, while the mean intensity is defined by the average intensity of all pixels of an object. For the background subtraction of HA, the mean intensity of all untransfected cells imaged on that slide multiplied by the area of the transfected cells was used. HA staining was used to identify transfected cells and intracellular localization of RhoA constructs. The area of nuclei and cells was detected using CellProfiler modules and staining with DAPI or phalloidin, respectively. Nuclear fraction was calculated by dividing the integrated intensity measured in the nuclear area by the integrated intensity measured in the whole cell. For the GFP transfection control, the nuclear fraction of GFP is calculated. All phalloidin and DAPI values of the transfected cells were normalized by the average values of the untransfected cells imaged in the same experiment to allow the combination of different experiments in a single figure. Integrated intensities are the sum of all intensities measured within an object (cell or nucleus). Mean intensities are the integrated intensities divided by the area of the corresponding object (cell or nucleus). The form factor is defined as the (perimeter)2/(4π × area), which will be 1 in the case of a circle. A higher value of the form factor indicates a more star-like shape. Eccentricity is defined as the ratio of the distance between the foci of the ellipse and its major axis length. It varies between 0 for a circle and 1 for a line segment and indicates cell elongation.

### 2.8. Statistical Analysis

Data were analyzed using Prism version 9 (GraphPad Software; La Jolla, CA, USA) and presented as mean ± standard deviation of at least four independent experiments. Statistical significance was determined by one-way ANOVA with Tukey’s multiple comparison test or, where indicated, by a *t*-test. The asterisks indicate significant differences (ns (nonsignificant): *p* ≥ 0.05; *: *p* < 0.05; **: *p* < 0.01; ***: *p* < 0.001; ****: *p* < 0.0001). A complete list of Tukey’s multiple comparison tests and *p* values is presented in Supplementary Table S3.

## 3. Results

### 3.1. Establishment of a Platform to Distinguish Nuclear and Cytoplasmic Functions of RhoA

**Figure 1:**
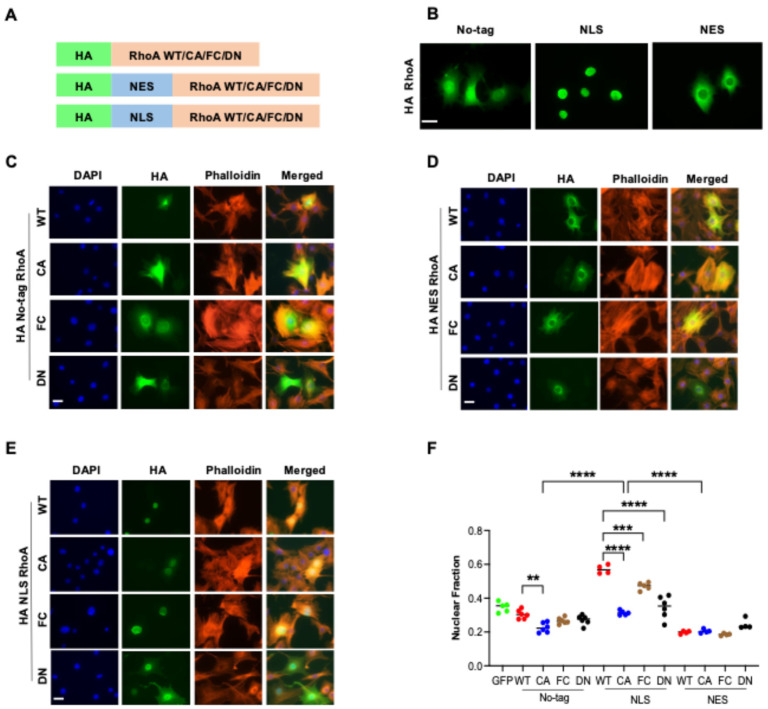
RhoA activation inhibits nuclear localization. (**A**) Schematic presentation of all RhoA constructs used, containing an HA tag for detection, and NES or NLS sequence for directing intracellular location and point mutants to increase (FC, CA) or decrease (DN) activation. (**B**) Immunofluorescence staining for HA indicating the intracellular distribution of no-tag, NLS, and NES RhoA in transiently transfected NIH 3T3 cells. (**C**–**E**) Fluorescence staining of NIH 3T3 cells transiently transfected with the indicated RhoA constructs and stained with DAPI (DNA), phalloidin (F-actin), and anti-HA (RhoA construct). Untransfected cells (UnTF) were stained as a control. Images were taken with an EVOS microscope at 40× magnification. (Scale bar = 40 µm.) (**F**) Nuclear fraction of indicated RhoA constructs or GFP transfection control quantified by CellProfiler. Each dot represents the average nuclear fraction of 100–150 analyzed transfected cells. *p*-values of selected comparisons are indicated by asterisks. Dot colors indicate type of mutation (**: *p* < 0.01; ***: *p* < 0.001; ****: *p* < 0.0001).

In order to distinguish the nuclear and cytoplasmic functions of RhoA, we fused RhoA with a nuclear localization signal (NLS) or a nuclear export sequence (NES) (Figure 1A). An HA-tag was added to specifically detect the RhoA fusion protein. To investigate the role of RhoA activation, several RhoA mutant forms with altered activation levels were generated. Firstly, a constitutively active mutant (CA; Q63L); secondly, a fast-cycling mutant (FC; F30L), which is more frequently active than the wildtype (WT) form due to a higher GDP to GTP exchange rate; and finally, a dominant negative mutant (DN; T19N), which is inactive and blocks activation of endogenous RhoA, RhoB, and RhoC by binding to Rho activating GEFs. We hypothesized that nucleus-specific effects will correlate with nuclear location and activation. To minimize potential compensatory effects in response to stable expression and to prevent selection for certain subclones during cell culture, NIH 3T3 cells were transiently transfected with WT RhoA (no-tag) or with NES- or NLS-tagged RhoA and stained after 48 h for the transfected RhoA protein by antibodies recognizing the HA-tag (Figure S1A,B). Fluorescence microscopy revealed that the NES- and NLS-tags strongly affected the distribution of RhoA. While no-tag RhoA was detected both in the cytoplasm and nucleus, NES was mostly in the cytoplasm and NLS mainly inside the nucleus (Figure 1B). Thus, a platform to check the nuclear and cytoplasmic functions of RhoA has been created.

### 3.2. RhoA Activation Inhibits Nuclear Localization

To detect the intracellular localization of RhoA, nuclei were identified by the DAPI staining of DNA, and cytoplasm was identified by the staining of F-actin with fluorescently labeled phalloidin. Images were quantified by CellProfiler (Figure 1C–E and Figures S1A and S2). The average HA expression of the cells selected for analysis was similar for the different RhoA constructs. No-tag WT RhoA was found both in the nucleus and cytoplasm with quite a lot of cell-to-cell variation (Figure 1F and Figure S1B). Importantly, the average nuclear fraction of no-tag WT RhoA was significantly higher than for NES WT RhoA, strongly suggesting that WT RhoA does indeed distribute to the nucleus. Increasing the activation of RhoA (FC, CA), which correlates with higher binding to effectors, significantly decreased the nuclear localization of the no-tag and, even more, of the NLS forms. While the NLS strongly increased the nuclear localization of WT RhoA, the effect was reduced for FC and was only very moderate for CA. Moreover, nuclear fraction was not significantly different for no-tag CA and NES CA, suggesting that constitutively active RhoA is hardly able to enter the nucleus in the absence of an NLS tag. Interestingly, NLS DN RhoA was less frequently located in the nucleus than NLS WT RhoA (Figure 1F). Possibly, cytoplasmic proteins binding strongly to DN RhoA but weakly to WT RhoA retain DN RhoA in the cytoplasm.

These results demonstrate that RhoA is unlikely to enter the nucleus in its activated form and imply that the activation of nuclear RhoA has to occur within the nucleus.

### 3.3. RhoA Regulates Cell Area and Shape by Total Level and Density of F-Actin

**Figure 2:**
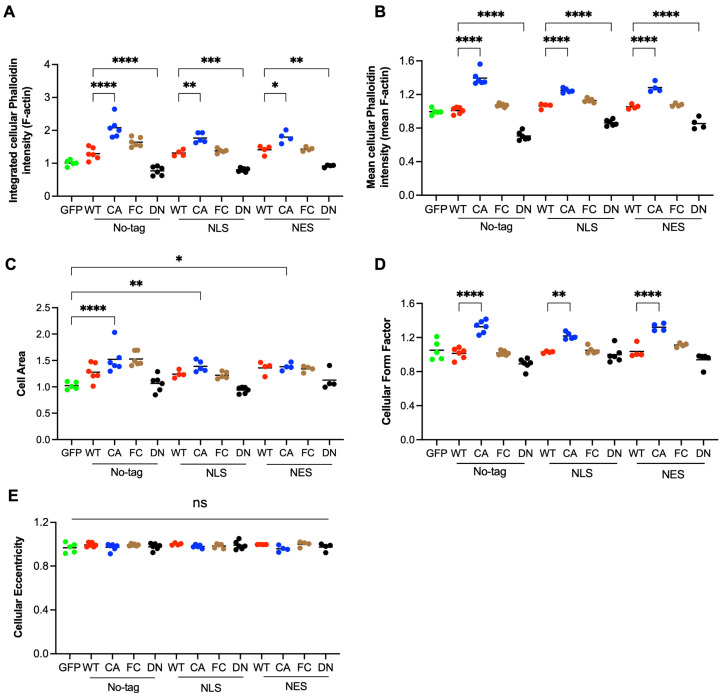
RhoA regulates cell area and shape by total cellular amount and density of F-actin. (**A**–**E**) Images as described in Figure 1 were quantitatively analyzed by CellProfiler. The averages of 4–6 independent experiments per indicated construct or GFP transfection control are shown, with each dot indicating one experiment of about 100–150 transfected cells. Data presented are fold changes compared to untransfected cells. (**A**) Integrated phalloidin staining (F-actin) per cell; (**B**) mean phalloidin staining (F-actin density) per cell; (**C**) cell area; (**D**) cell form factor; and (**E**) cell eccentricity. *p*-values of selected comparisons are indicated by asterisks. Dot colors indicate type of mutation (ns: *p* ≥ 0.05; *: *p* < 0.05; **: *p* < 0.01; ***: *p* < 0.001; ****: *p* < 0.0001).

RhoA is well known to induce the formation of F-actin and its bundling to stress fibers. As expected, the ectopic expression of WT and even more of activated RhoA (FC, CA) increased the amount of F-actin and strong stress fibers compared to untransfected cells (Figure 1C–E and Figure 2A). In the case of CA RhoA, the density of F-actin was also significantly increased (Figure 2B). In contrast, DN RhoA decreased the cellular amount and concentration of F-actin (Figure 2B). The expression of GFP did not affect F-actin compared to untransfected cells. Unexpectedly, strongly altering the intracellular location of RhoA by NLS- or NES-tags had only a minor, non-significant effect on cellular actin polymerization, suggesting that even the low cytoplasmic levels of NLS RhoA are sufficient to saturate cytoplasmic Rho effectors regulating actin polymerization.

Cellular F-actin levels strongly correlated with the cell area, indicating an important role of RhoA-dependent changes in the actin cytoskeleton of the cell area (Figure 2C and Figure S3). RhoA activation resulted in increased F-actin density that was visible for all CA forms, maybe because the cell area increase reached a maximum (Figure 2B,C). With respect to cell shape, the form factor was increased for CA RhoA indicating a more star-shaped perimeter and correlating roughly with changes in F-actin density (Figure 2B,D). Cell eccentricity was not significantly altered by any of the ectopically expressed RhoA forms, indicating no strong change in cell elongation (Figure 2E).

### 3.4. Activation of RhoA in the Nucleus Is Not Crucial for Nuclear Actin Polymerization

**Figure 3:**
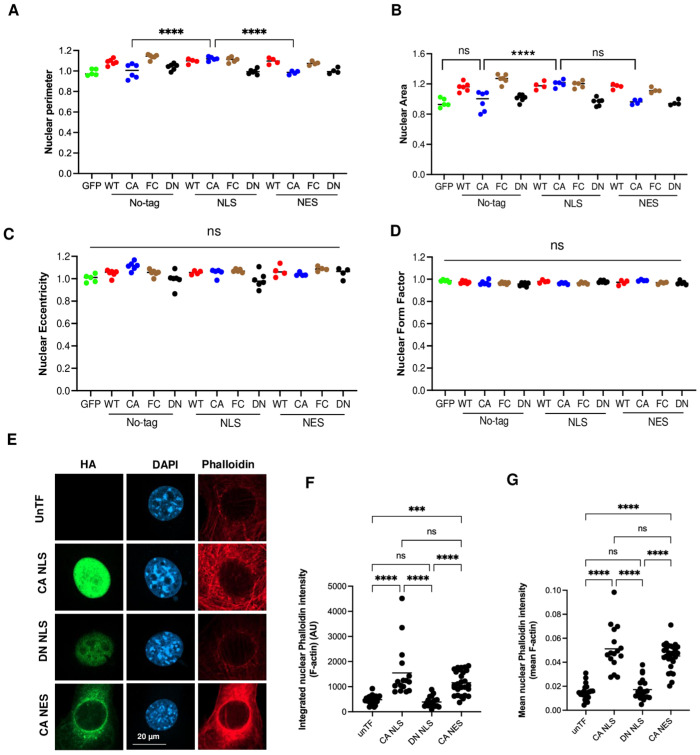
The activation of RhoA in the nucleus regulates the nuclear area but is not crucial for nuclear actin polymerization. (**A**–**E**) Images as described in Figure 1 were quantitatively analyzed by CellProfiler. The averages of 4–6 independent experiments per indicated construct or GFP transfection control are shown, with each dot indicating one experiment of about 100–150 transfected cells for the nuclear area. (**A**) Nuclear perimeter; (**B**) nuclear area; (**C**) nuclear eccentricity; and (**D**) nuclear form factor. Data presented are fold changes compared to untransfected cells. *p*-values of selected comparisons are indicated by asterisks (ns: *p* > 0.05; ***: *p* < 0.001; ****: *p* < 0.0001). (**E**) Confocal z-stack images of NIH 3T3 cells transiently transfected with indicated RhoA constructs and fluorescently stained with DAPI (DNA), phalloidin (F-actin), and anti-HA (RhoA construct). Cells without HA signal in the same image were identified as untransfected (unTF) and used as a control. The z-plane with the highest DAPI intensity is shown (Scale bar = 20 µm). (**F**,**G**) A quantitative analysis of confocal images of cells transfected with indicated RhoA constructs or untransfected (unTF) using CellProfiler. Each dot represents the integrated (**F**) or mean (**G**) intensity of phalloidin staining (F-actin) per nucleus in the z-plane with the highest DAPI intensity. Dot colors in A-D indicate type of mutation (n: 16–27 cells; ns: *p* ≥ 0.05; ***: *p* < 0.001; ****: *p* < 0.0001).

We then assessed the role of RhoA-dependent actin polymerization in changes to nuclear size and shape since cytoplasmic F-actin is connected to the nuclear membrane via the LINC complex [6]. Furthermore, nuclear actin polymerization might contribute to nuclear morphology. The expression of NLS CA RhoA significantly increased nuclear perimeter and area compared to NES CA RhoA, despite a comparable effect of both constructs on cytoplasmic F-actin (Figure 3A,B). Furthermore, inhibiting cytoplasmic F-actin by NES DN RhoA displayed no significant effect on the nuclear area. No changes were observed with respect to the eccentricity and nuclear form factor (Figure 3C,D). These data indicate that nuclear morphology in NIH 3T3 cells is independent of cytoplasmic RhoA activation but dependent on nuclear RhoA activity.

To assess whether nuclear actin polymerization is mediating the observed RhoA-dependent increase in nuclear areas, we performed confocal microscopy and quantified nuclear F-actin in the focal plane with the highest intensity of DAPI staining (Figure 3E and Figure S4A,B). Surprisingly, NLS CA and NES CA showed no significant difference in total levels or density of nuclear F-actin, suggesting firstly, that nuclear RhoA activation is not causing strong nuclear F-actin formation and, secondly, that nuclear RhoA activation increases nuclear size in an F-actin-independent manner (Figure 3F,G). Untransfected cells and cells transfected with NLS DN RhoA showed lower levels of nuclear F-actin than NLS CA and NES CA, which is most likely caused by the insufficient exclusion of cytoplasmic F-actin by confocal microscopy. A movie made from the z-stack analysis supported the notion that actin fibers apparently within the nuclear area in the xy plane picture are in fact above the nucleus (Movie S1).

These data do not indicate an important role for RhoA in the regulation of nuclear F-actin.

### 3.5. Nuclear RhoA Activity Regulates DNA Amounts

As nuclear size can correlate with the amount of DNA, for example, during the cell cycle, we tested whether cytoplasmic or nuclear RhoA activation affects DNA levels as measured by DAPI staining 48 h after transfection.

**Figure 4:**
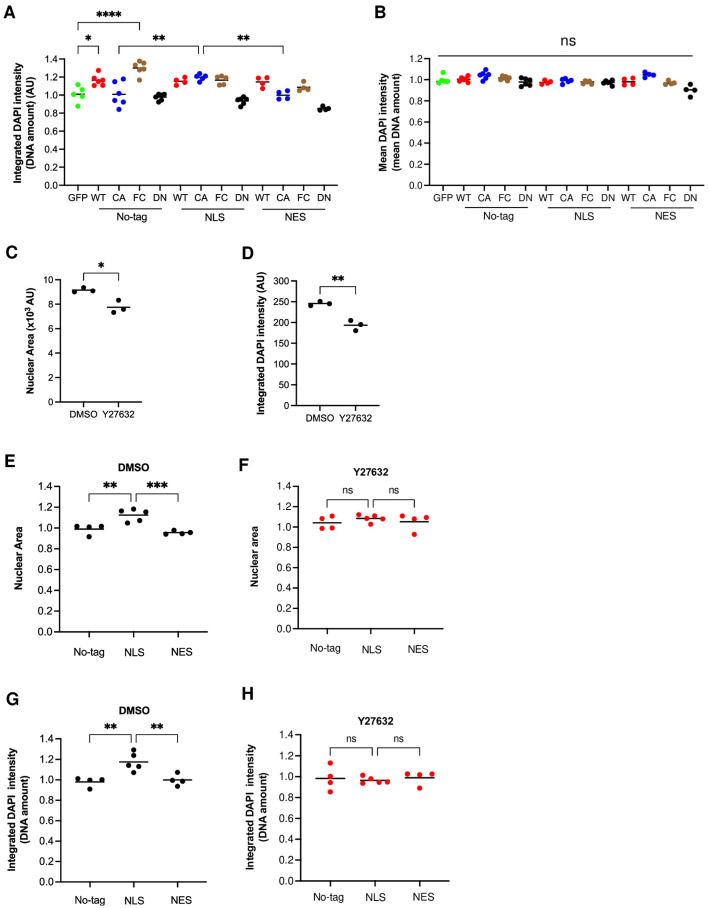
Nuclear RhoA regulates DNA amounts via ROCK activation. (**A**,**B**) Images as described in Figure 1 were quantitatively analyzed by CellProfiler. The averages of 4–6 independent experiments per indicated construct or GFP transfection control are shown, with each dot indicating one experiment of about 100–150 transfected cells. (**A**) Integrated DAPI intensity per nucleus (DNA amount); (**B**) mean DAPI intensity per nucleus (DNA density). (**C**,**D**) Fluorescence staining of NIH 3T3 cells treated for 24 h with DMSO (control) or the ROCK inhibitor Y27632 with DAPI (DNA) Each dot represents the average of an independent experiment with 600–700 cells. (**C**) Nuclear area; (**D**) integrated DAPI intensity per nucleus (DNA amount). (**E**–**H**) Fluorescence staining of NIH 3T3 cells transfected with the indicated CA RhoA constructs, treated for 24 h with DMSO (control) or the ROCK inhibitor Y27632 (10 μM), and stained with DAPI (DNA). Each dot represents the average of an independent experiment with 100–150 transfected cells and is presented as fold changes compared to DMSO-treated no-tag RhoA. (**E**,**F**) Nuclear area; (**G**,**H**) integrated DAPI intensity per nucleus (DNA amount). Dot colors indicate type of mutation in A, B and the type of treatment in E-H (ns: *p* ≥ 0.05; *: *p* < 0.05; **: *p* < 0.01; ***: *p* < 0.001; ****: *p* < 0.0001; For C and D a *t*-test was carried out).

Indeed, CA RhoA promoted DNA amounts, but only when fused with NLS (Figure 4A). Apparently, nuclear amounts of no-tag and NES CA RhoA were too low to affect DNA levels. No alteration of DNA density was observed (Figure 4B). These data demonstrate that nuclear, but not cytoplasmic RhoA activation, is regulating DNA amounts in the system tested. Moreover, even strong cytoplasmic actin polymerization as induced by NES and no-tag CA RhoA (Figure 2A,B) was not affecting DNA amounts. WT RhoA increased DNA amounts independent of the tag, suggesting that it can enter the nucleus in the GDP-bound form and become activated within the nucleus. DN RhoA showed no significant effect on DNA amounts (Figure 4A).

In all transfected cells, DNA amounts were strongly correlated with the nuclear area (Figure S5), indicating that nuclear RhoA activation increases the nuclear area by promoting DNA content, conceivably by cell cycle regulation.

### 3.6. Nuclear RhoA Regulates DNA Amounts via ROCK Activation

Since the serine/threonine kinases ROCK1 and ROCK2 were shown previously to be able to regulate the cell cycle [28], we hypothesized that endogenous nuclear RhoA activation controls DNA amounts via ROCK. If so, the inhibition of ROCK should reduce DNA amounts in non-transfected cells and prevent the NLS CA RhoA-induced increase in DNA. Indeed, in untransfected cells, the ROCK inhibitor Y27632 significantly decreased nuclear area and DNA amounts (Figure 4C,D). In addition, it reduced the total F-actin, F-actin concentration, and cell area (Figure S6A–D). In transfected cells, Y27632 completely abrogated the NLS CA RhoA-induced increase in nuclear area (Figure 4E,F) and DNA amounts (Figure 4G,H), compared to no-tag or NES CA RhoA. (Note that the data shown Figure 4F,H were normalized to untransfected cells treated with Y27632.) Y27632 reduced the nuclear fraction for no-tag CA RhoA, but not for NLS and NES CA RhoA (Figure S6E). Apparently, active ROCK contributes to the nuclear retention of untagged RhoA.

These data suggest that endogenous nuclear RhoA activation promotes an increase in nuclear area and DNA content in a ROCK-dependent manner.

### 3.7. No Specific Regulation of DNA Amounts by Nuclear Rac1 and Cdc42

RhoA can crosstalk to other Rho GTPases such as Rac1 and Cdc42 which, therefore, might be downstream mediators of certain RhoA effects. To elucidate whether the effect of nuclear RhoA could be mediated by Rac1 or Cdc42, we investigated the function of Rac1 and Cdc42 by tagging them with NLS and NES and by altering their activation by point mutations by quantitative image analysis of fluorescent staining (Figure S7). As for activated RhoA, the activated forms of Rac1 and Cdc42 showed reduced nuclear localization (Figure S8A,B). However, the expression of the NLS-tagged forms of CA Rac1 and CA Cdc42 did not result in an increased nuclear area or increased DNA amount relative to no-tagged or NES-tagged forms of Rac1 or Cdc42 (Figure S8C–F). These data suggest that the decreased nuclear localization of activated Rho GTPases is a general feature of at least RhoA, Rac1, and Cdc42 and not restricted to RhoA. The results do not support that the nuclear effect of RhoA is mediated by nuclear Rac1 or Cdc42.

### 3.8. Nuclear RhoA-ROCK Signaling Regulated DNA Amounts via Nuclear pErk

Erk kinase, which is present both in the cytoplasm and nucleus, is an important regulator of the cell cycle [29]. Earlier, ROCK was suggested to promote cell cycle progression by activation of Erk [30].

**Figure 5:**
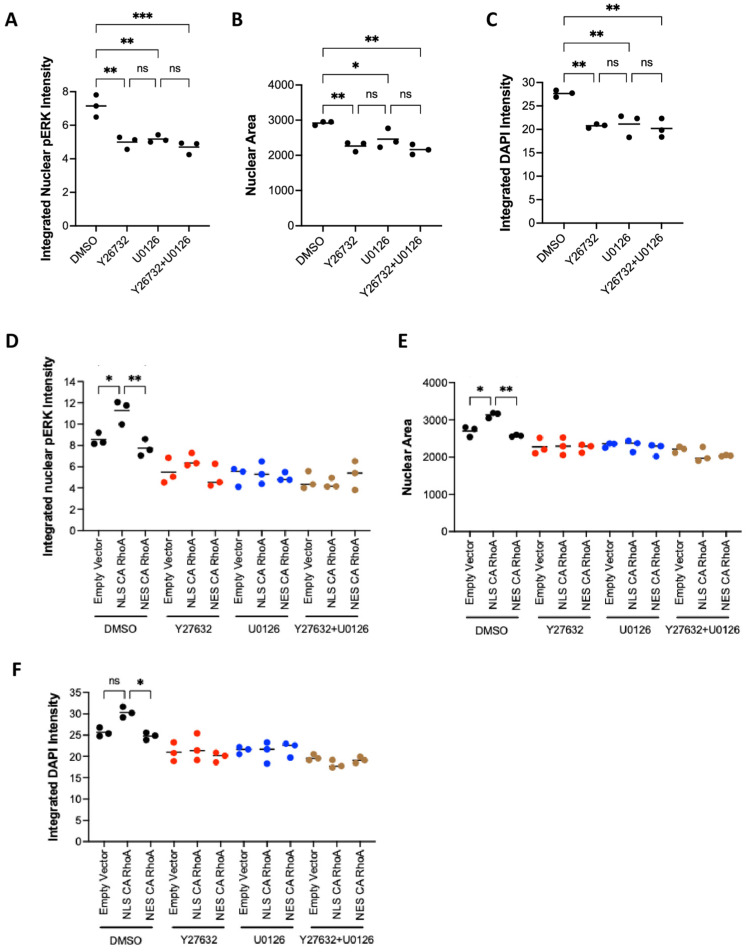
Nuclear RhoA regulates nuclear pErk via ROCK activation. (**A**–**C**) NIH 3T3 cells were treated with the indicated inhibitors for 24 h (Y27632; 10 μM) or 3h (U0126; 10 μM) and fluorescently stained for DNA with DAPI and for nuclear pErk (npErk). Each dot represents the average of an independent experiment with 10,000–15,000 cells. (**A**) npErk; (**B**) nuclear area; (**C**) integrated DAPI intensity per nucleus (DNA amount). (**D**–**F**) Fluorescence staining of NIH 3T3 cells transfected with the indicated RhoA CA constructs and treated with the indicated inhibitors. Each dot represents the average of an independent experiment with 4000–9000 cells. (**D**) npErk; (**E**) nuclear area; (**F**) integrated DAPI intensity per nucleus (DNA amount). Dot colors indicate similar inhibitor treatment in D-F (ns: *p* ≥ 0.05; *: *p* < 0.05; **: *p* < 0.01; ***: *p* < 0.001).

To assess whether the endogenous activation of ROCK is influencing nuclear levels of activated, phosphorylated Erk (npErk), we treated NIH 3T3 cells with inhibitors for ROCK (Y27632) and for the essential Erk activator Mek (U0126). ROCK inhibition significantly reduced npErk, nuclear area, and DNA amount to a similar extent as U0126 (Figure 5A–C). The combination of both inhibitors did not show an additive effect. These results show that ROCK is upstream of npErk and that ROCK is controlling DNA amounts via Erk activation.

We then tested whether the activation of RhoA in the nucleus or in the cytoplasm is regulating npErk. Only NLS CA RhoA and not cytoplasm-restricted NES CA RhoA significantly increased npErk, suggesting that only the nuclear activation of RhoA is able to increase npErk (Figure 5D and Figure S9). The addition of Y27632, U0126, or a combination of both abrogated the effect and reduced npErk, nuclear area, and DNA amount compared to untreated cells (Figure 5E,F).

These results indicate that nuclear RhoA controls DNA amounts by the regulation of npErk levels.

## 4. Discussion

The data presented in this study suggest that endogenous nuclear RhoA contributes via ROCK to the regulation of npErk, which controls DNA amounts. The inhibition of the RhoA effector ROCK reduced both npErk as well as DNA amounts, indicating that endogenous ROCK activation controls DNA replication in NIH 3T3 cells. Moreover, the nucleus-targeted NLS CA RhoA but not the cytoplasm-targeted NES RhoA increased npErk and DNA amounts, suggesting that nuclear RhoA regulation is required for npErk and cell cycle regulation. Both NLS CA RhoA and NES CA RhoA showed a similar increase in total cytoplasmic F-actin and cell area, indicating that the lack of nuclear effect of NES CA RhoA is not due to a reduced signaling activity of this construct.

RhoA activation has been connected with both increased and decreased proliferation suggesting a high context dependency [31,32,33]. The induced expression of CA RhoA was reported to increase the proportion of G2/M cells and binucleation in a ROCK-independent manner [32]. The specific role of nuclear RhoA signaling in cell cycle regulation was not investigated in these studies, due to the inability to selectively control cytoplasmic and nuclear RhoA activation. Using our novel platform, we describe here that nuclear, but not cytoplasmic RhoA activity regulates DNA amounts in NIH 3T3 cells under culture conditions in the presence of serum. These altered DNA amounts might be caused by altered cell cycle regulation but could also be due to alterations in DNA replication and replication stress. Attempts by us to investigate the cell cycle of transiently transfected cells by measuring DNA amounts and EdU incorporation, unfortunately, resulted in insufficient cell numbers for analysis. Future cell cycle studies should be carried out in stably transfected cells with a similar expression level of a RhoA construct in all cells which is not changing during culture. Further experiments with such cells should give insight into the role of nuclear RhoA/ROCK signaling in DNA replication, replication stress, and DNA repair.

Using the transient expression of WT RhoA—tagged with HA alone or with NLS or NES as well—fluorescent staining, and quantitative image analysis, we confirmed that RhoA translocates to the nucleus. This was expected because of its small size and was reported earlier based on cell fractionation and GFP-fusion protein studies [11,12,13]. However, the nuclear translocation of RhoA occurred mainly in the GDP-bound form. Increasing the fraction of RhoA GTP by point mutations (WT -> FC -> CA) correlated with reduced nuclear translocation. Furthermore, no significant difference was found between the nuclear fraction of no-tag CA RhoA and NES CA RhoA. Only tagging by NLS was able to move significant amounts of CA RhoA to the nucleus. Along that line, only NLS CA RhoA, but not no-tag CA RhoA or NES CA RhoA, increased DNA amounts, suggesting that RhoA GTP is not translocating to the nucleus in functionally relevant amounts. Cytoplasmic and nuclear RhoA signaling is therefore a relatively independent process and nuclear RhoA signaling requires RhoA activation inside the nucleus. RhoA-specific GEFs (RASGRF2, ARHGEF3, Net1, and ECT2) [5] and GAPs (ARHGAP11A, ARHGAP19, and ARHGAP31) [5] were reported to localize in the nucleus and are therefore expected to be major regulators of nuclear RhoA signaling. A possible reason for the cytoplasmic restriction of CA RhoA could be the complex formation with RhoA effectors decreasing diffusion through the nuclear pores due to increased size [11]. Unexpectedly, GDP-bound DN RhoA was less able to enter the nucleus than WT RhoA, suggesting that the DN mutation interfered with nuclear localization. The strong binding of DN RhoA to cytoplasmic GEFs might be an underlying factor. Our data also imply that CA and DN RhoA are of limited value to study the nuclear function of RhoA if they are not tagged with NLS. Different biological effects observed for WT, FC, and CA RhoA might therefore partially depend on the absence of nuclear effects for untagged CA RhoA [8]. Also, the activated forms of Rac1 and Cdc42 showed reduced presence in the nucleus compared to wildtype, suggesting that binding to Rho GTPase effectors, in general, is inhibiting nuclear translocation and not binding to a translocation effector specific for RhoA, Rac1, or Cdc42. In addition to NLS- and NES-tagged forms of CA RhoA, nuclear RhoA signaling could also be studied by KO of nucleus-specific GEFs and GAPs for RhoA, or by NLS-tagged variants of them. However, many GEFs and GAPs are not specific for RhoA, which could complicate the interpretation and expression of GEFs is not equal to the activation of GEFs. The deletion of endogenous RhoA results in most cases in a compensatory upregulation of the closely related RhoB, which will make it difficult to study the function of endogenous RhoA by this approach. Inhibition of endogenous RhoA, RhoB, and RhoC with DN RhoA is better suited to investigate the function of physiological levels of nuclear RhoA. However, with the number of repeats carried out in this study, we were not able to show significant effects.

Surprisingly, even a strong increase in CA RhoA in the nucleus did not result in an obvious elevation of nuclear F-actin detectable by phalloidin staining. Earlier, the activation of the RhoA effectors mDia1 and mDia2 were described to promote nuclear F-actin formation [21] and nuclear RhoA-GTP would therefore be expected to promote nuclear actin polymerization. In addition, the activation of the Rho effector ROCK could induce LIMK-dependent phosphorylation of cofilin [34], which will decrease cofilin-dependent severing of F-actin and contribute to increased nuclear F-actin. Indeed, nuclear cofilin levels were shown to correlate with nuclear F-actin in mouse zygotes [35,36]. On the other hand, unphosphorylated cofilin was found to be crucial for the transporting of G-actin to the nucleus and phosphorylation; the deletion of the cofilin gene can reduce nuclear actin polymerization [37]. Our data now reveal that RhoA activation is not a major limiting factor in regulating nuclear actin polymerization in NIH 3T3 cells. The reason for this could be low nuclear levels of mDia1 and mDia2a or a low concentration of G-actin in the nucleus caused by an impaired cofilin-mediated nuclear import of G-actin or by a high nuclear export of G-actin. In addition, the strong reduction in the G-actin pool in the cytoplasm due to the massive polymerization of actin triggered by CA RhoA might lead to a netto efflux of G-actin from the nucleus which decreases nuclear F-actin formation [25].

Studying the cell compartment-specific function of RhoA is difficult, since NLS CA RhoA, although preferentially located in the nucleus, apparently had cytoplasmic levels sufficient to saturate the RhoA-dependent actin polymerization machinery. Conversely, NES-tagged WT RhoA, primarily located in the cytoplasm, showed sufficient nuclear levels to saturate the RhoA/ROCK-dependent cell cycle regulation. Constitutive activation of RhoA reduced the nuclear concentration of activated RhoA to such an extent that no cell cycle regulation could be observed in the absence of an NLS tag, thus enabling us to establish the first cellular system to study nucleus-specific RhoA signaling. This system will be useful in the future to evaluate in more detail how RhoA activation is influencing cell cycle or transcription.

## Data Availability

All data are contained within the manuscript.

## Supplementary Materials

The following supporting information can be downloaded at https://www.mdpi.com/article/10.3390/cells14060404/s1. Figure S1: RhoA displays a high cell-to-cell variation in intracellular localization. (A) Schematic illustration of experimental setup including seeding, transfection, reseeding, fixation, fluorescence microscopy, and image analysis with Cellprofiler including object identification and statistics. (B) Immunofluorescence staining showing different intracellular distributions of no-tag RhoA in transiently transfected NIH 3T3 cells (Scale bar = 40 µm). Figure S2: High-resolution images of fluorescent microscopy presented in Figure 1C–E. Figure S3: Cell area is highly correlated with integrated F-actin intensity. Images as described in Figure 1 were quantitatively analyzed by CellProfiler. The cell area was plotted against integrated phalloidin staining (F-actin) per cell revealing a high correlation as indicated by the high values for the coefficient of correlation (R^2^). Each dot represents a transfected cell. (n: 400–800). Figure S4: Nuclear RhoA activation does not result in obvious nuclear actin polymerization. (A,B) Confocal z-stack images of NIH 3T3 cells transiently transfected with NES CA RhoA (A) and NLS CA RhoA (B) and fluorescently stained with DAPI (DNA), phalloidin (F-actin), and anti-HA (RhoA construct). Representative stack analyses are shown (n: 16, 27) (Scale bar = 20 µm). Figure S5: Nuclear area strongly correlates with DNA amounts. Images as described in Figure 1 were quantitatively analyzed by CellProfiler. The nuclear area was plotted against integrated DAPI staining (DNA) per cell revealing a high correlation as indicated by the high values for the coefficient of determination (R^2^). Figure S6: ROCK inhibition decreases cellular F-actin and cell area. (A–D) Fluorescence staining of NIH 3T3 cells treated for 24 h with DMSO (control) or the ROCK inhibitor Y27632 (10 μM), and stained with DAPI (DNA), phalloidin (F-actin), and anti-HA (RhoA construct). Each dot represents the average of an independent experiment with 600–700 nuclei. (A) Integrated phalloidin intensity (F-actin) per cell; (B) mean phalloidin intensity per cell (F-actin density); (C) cell area; (D) mean DAPI intensity per nucleus (DNA density). (E) NIH 3T3 cells transfected with the indicated CA RhoA constructs were treated for 24 h with DMSO (control) or the ROCK inhibitor Y27632 (10 μM). The nuclear fraction of the transfected HA-tagged RhoA constructs detected by HA staining is shown. The cell area was identified by phalloidin staining. Each dot represents the average of an independent experiment with 100–150 cells. (ns: *p* ≥ 0.05; *: *p* < 0.05; **: *p* < 0.01; ***: *p* < 0.001). Figure S7. The activation of Rac1 or Cdc42 inhibits their nuclear localization. (A–F) Fluorescence staining of NIH 3T3 cells transiently transfected with the indicated Rac1 or Cdc42 constructs and stained with DAPI (DNA), phalloidin (F-actin), and anti-HA (Rac1 or Cdc42 construct). Untransfected cells (UnTF) were stained as a control. Images were taken with an EVOS microscope at 40x magnification. (Scale bar = 40 µm). Figure S8. Activated Rac1 and Cdc42 do not efficiently translocate to the nucleus. (A–F) Images as shown in Figure S7 were quantitatively analyzed by CellProfiler. The averages of 3–6 independent experiments per indicated construct or GFP transfection control are shown, with each dot indicating one experiment of about 100–150 transfected cells. (A, B) Nuclear fraction of indicated Rac1 and Cdc42 constructs; (C,D) nuclear area and integrated DAPI intensity per nucleus (DNA amount) for indicated Rac1 constructs; (E,F) nuclear area and integrated DAPI intensity per nucleus (DNA amount) for indicated Cdc42 constructs (ns: *p* ≥ 0.05; *: *p* < 0.05; **: *p* < 0.01; ***: *p* < 0.001; ****: *p* < 0.0001). Figure S9: Nuclear RhoA regulates nuclear pErk via ROCK activation. Example images for the experiments quantified in Figure 5D–F. Table S1: Primers used to add HA-, NES-, or NLS-tags to the N-terminus of RhoA of Rac1 and Cdc42. Table S2: Modules and settings of the CellProfiler pipeline used for analysis; Table S3: Tukey’s multiple comparisons test and *p*-value for all experiments. Movie S1: Nuclear RhoA activation does not result in obvious nuclear actin polymerization. Movie of a 3D reconstruction of a Z-stack confocal analysis of NIH 3T3 cells transiently transfected with NES CA RhoA and fluorescently stained with DAPI (DNA), phalloidin (F-actin), and anti-HA (RhoA construct). Actin fibers visible in the nuclear area in the xy plane appear clearly above the nucleus in 3D.
