# Supplementary material for: Nuclear RhoA Activation Regulates Nucleus Size and DNA Content via Nuclear Activation of ROCK and pErk
Source: Cells. 2025 Mar 10;14(6):404. doi: 10.3390/cells14060404 (PMC11941300; doi:10.3390/cells14060404)
Supplement: Supplementary file 1 [file cells-14-00404-s001.zip › Suppl. Material f.pdf]

Figure S1: Wild-type RhoA displays a wide variety of distribution between nucleus and cytoplasm

A

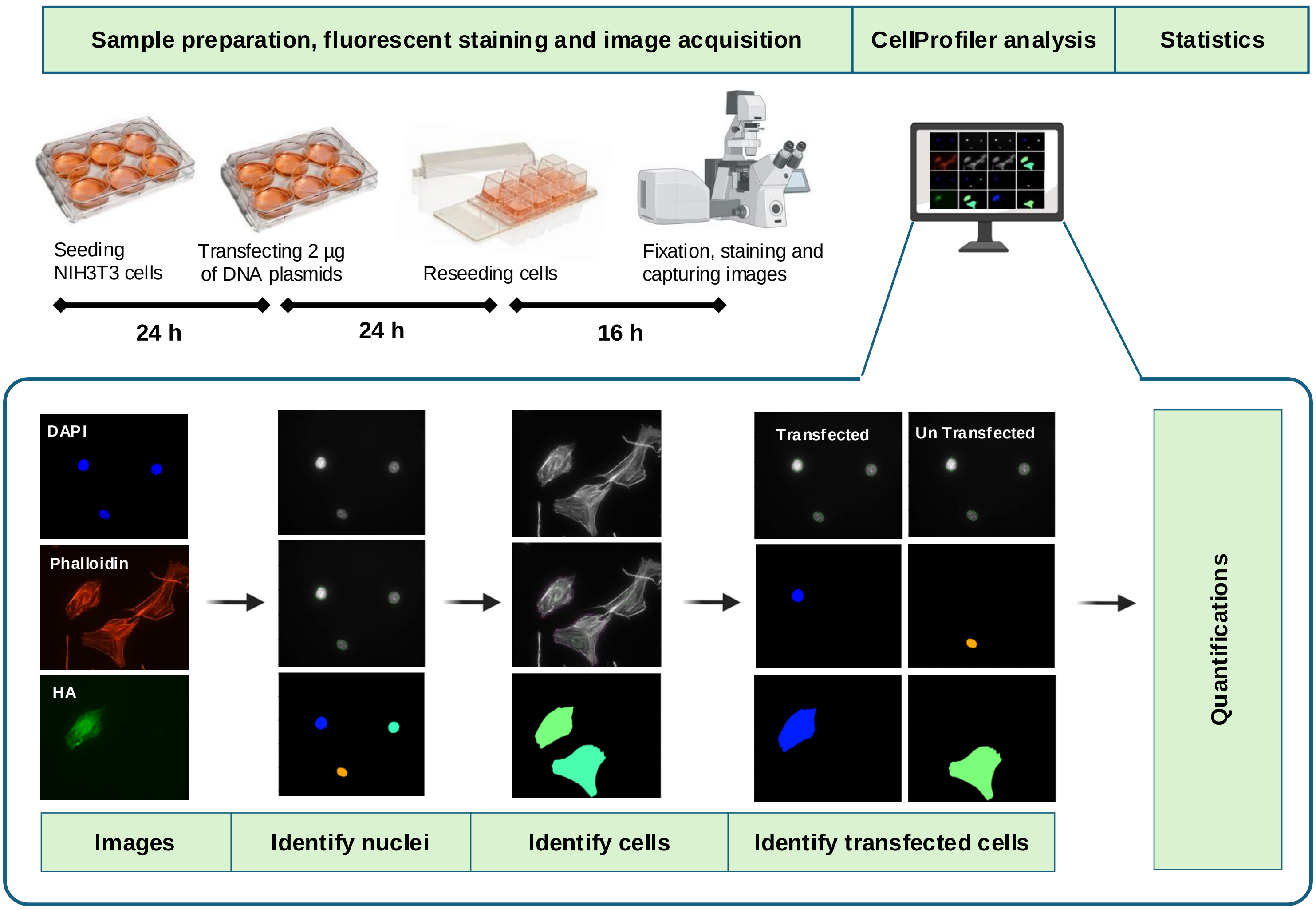

B

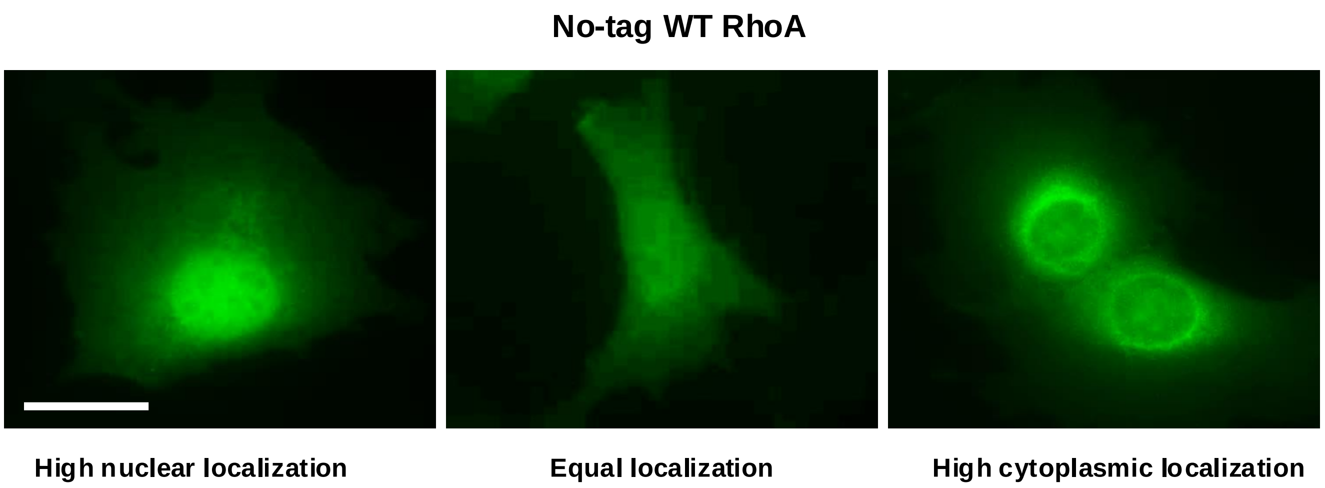

Figure S2: High resolution images of fluorescent microscopy presented in Figure 1 C-E

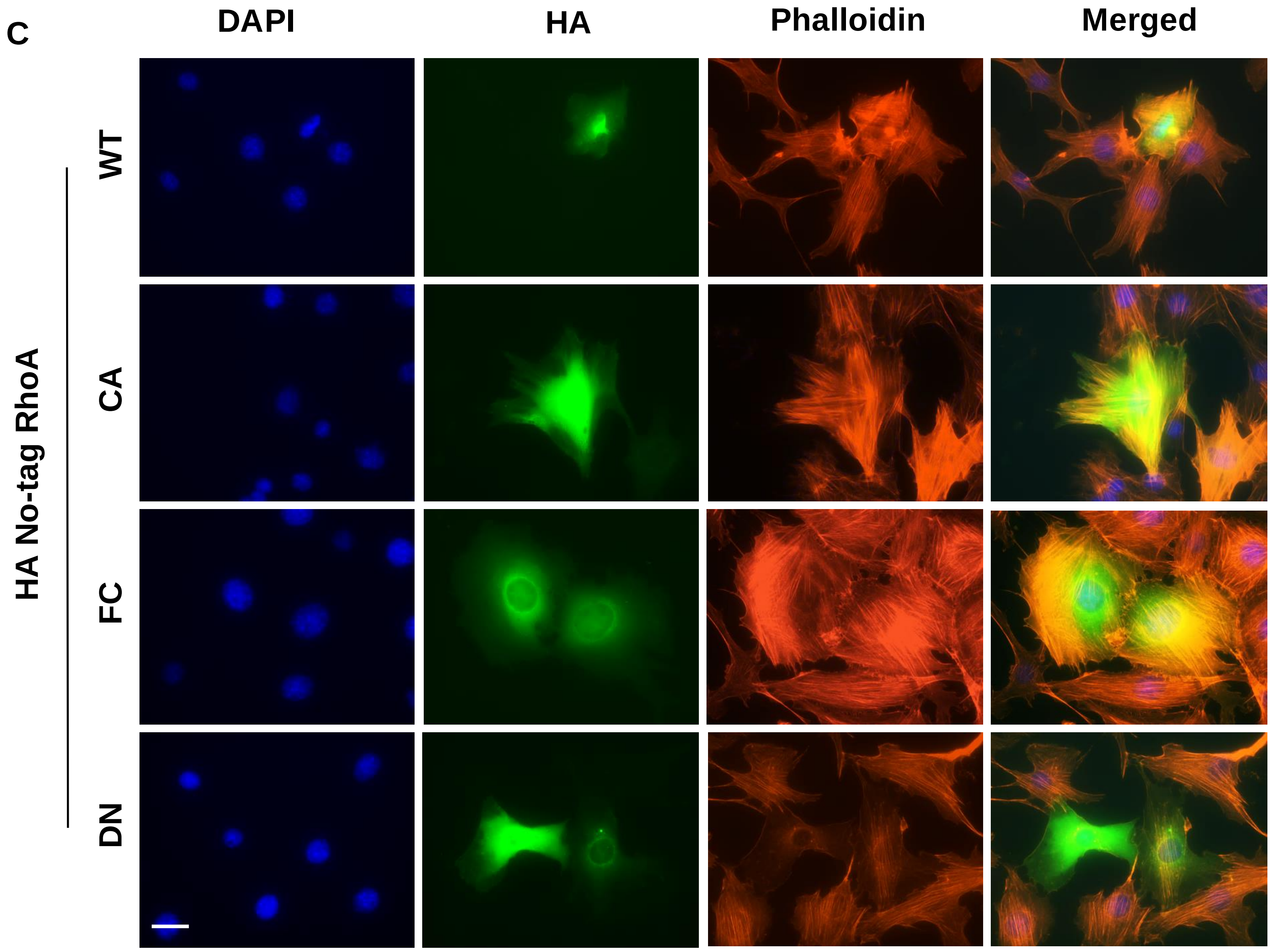

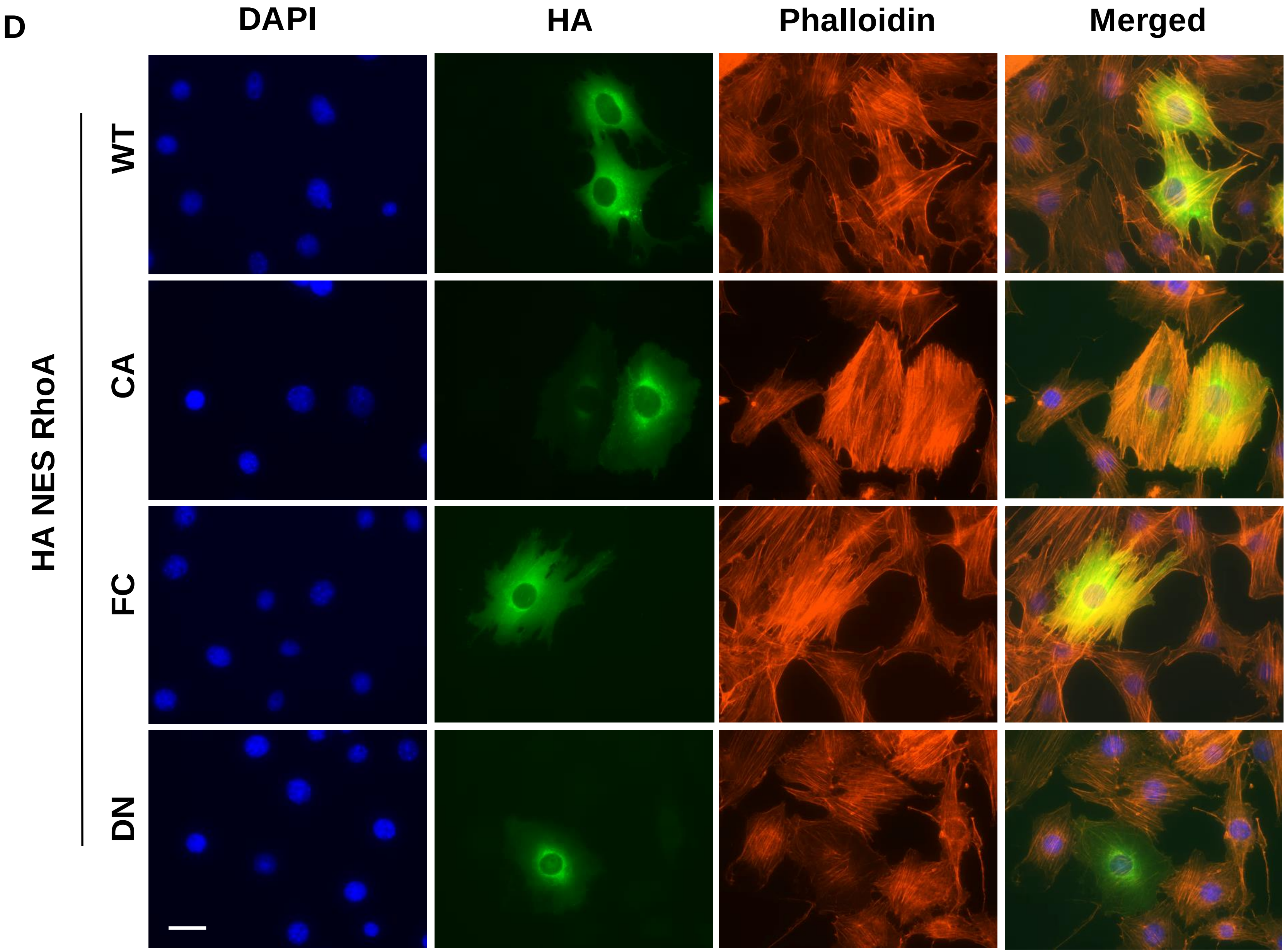

D
DAPI
HA
Phalloidin
Merged
HA NES RhoA
WT
CA
FC
DN

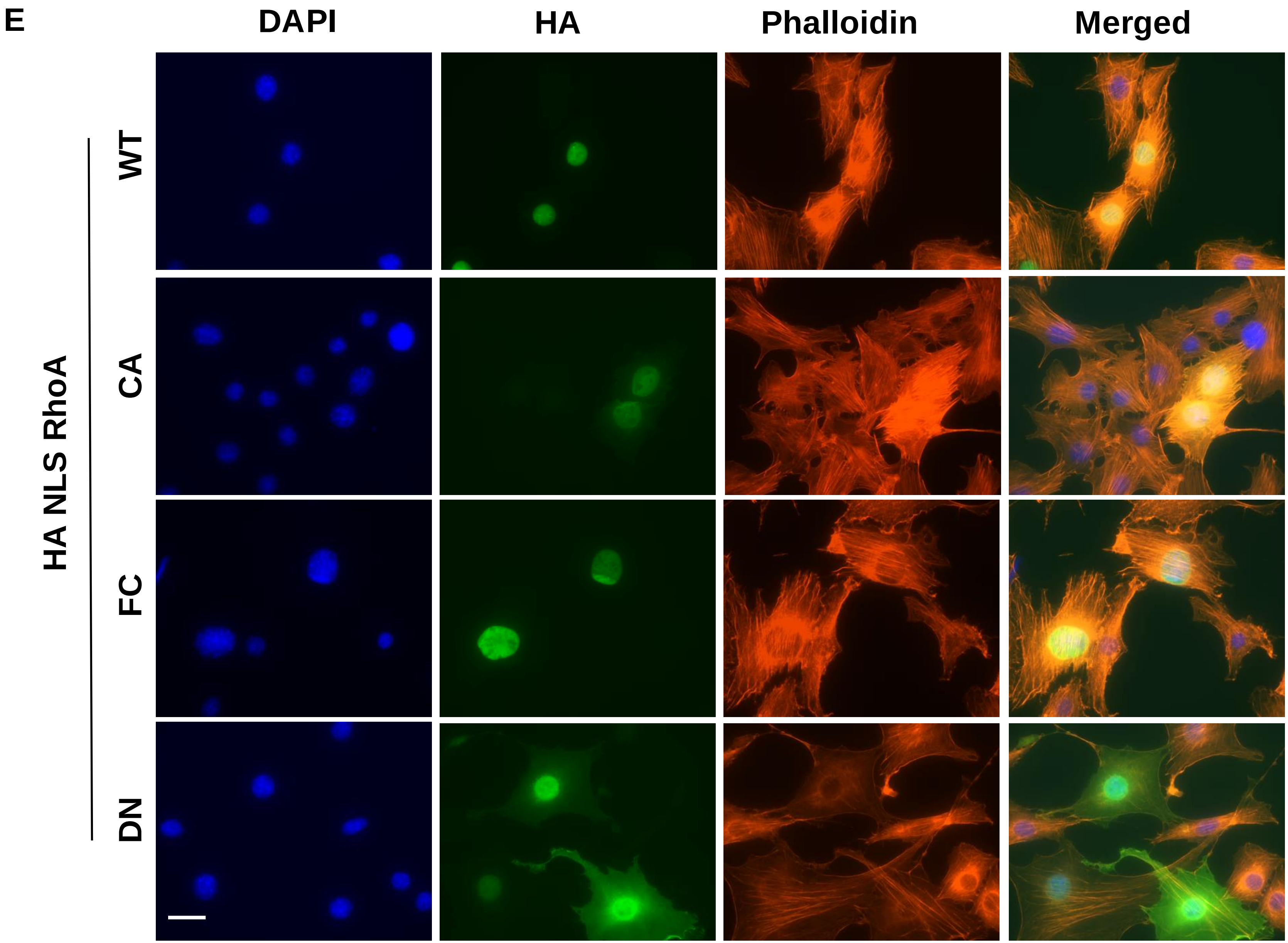
E
DAPI
HA
Phalloidin
Merged
HA NLS RhoA
WT
CA
FC
DN

Figure S3: Cell area is highly correlated with total cellular F-actin levels

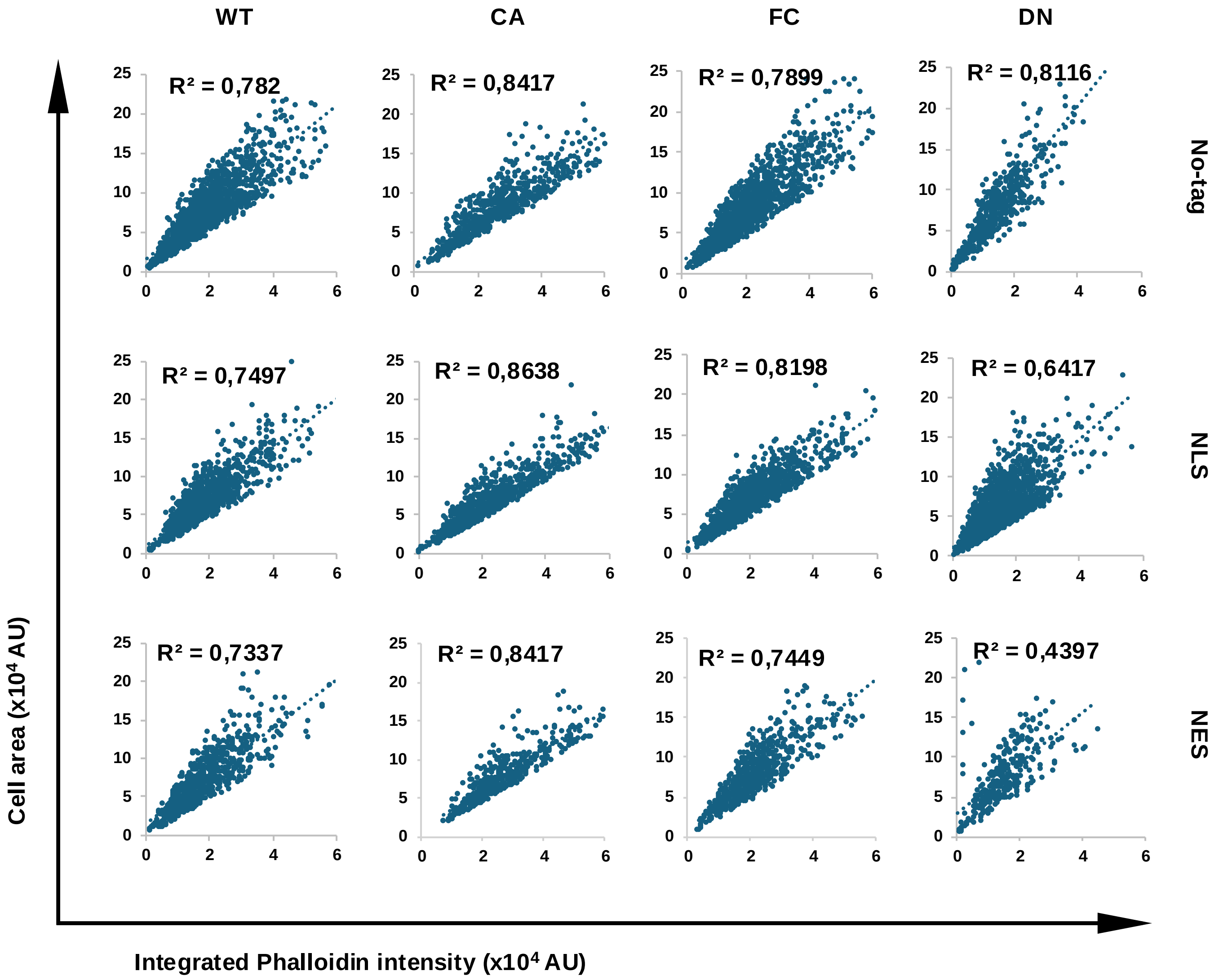

Figure S4: Nuclear activation of RhoA does not result in strong F-actin formation

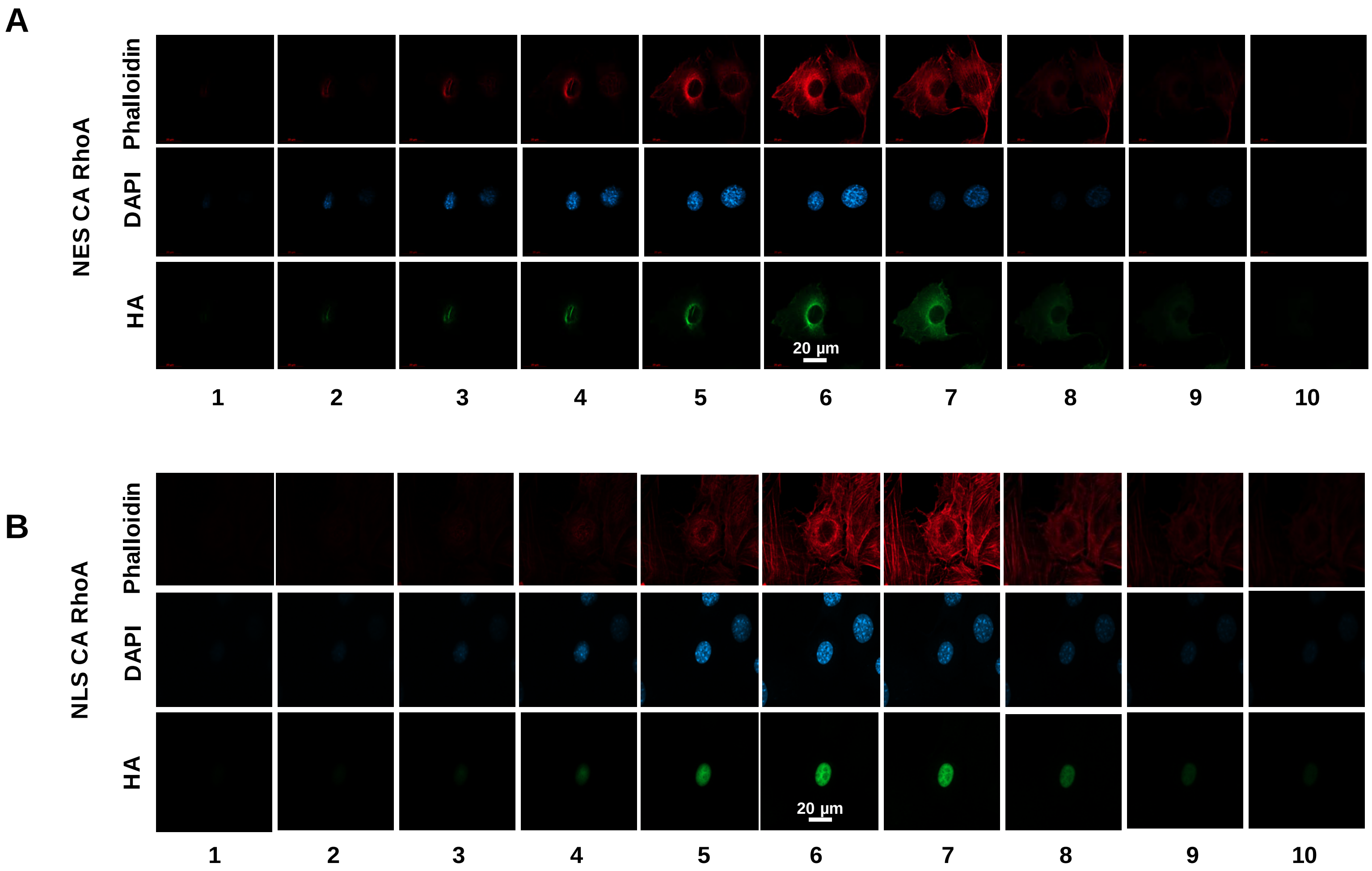

Figure S5: Nuclear area is highly correlated with DNA levels

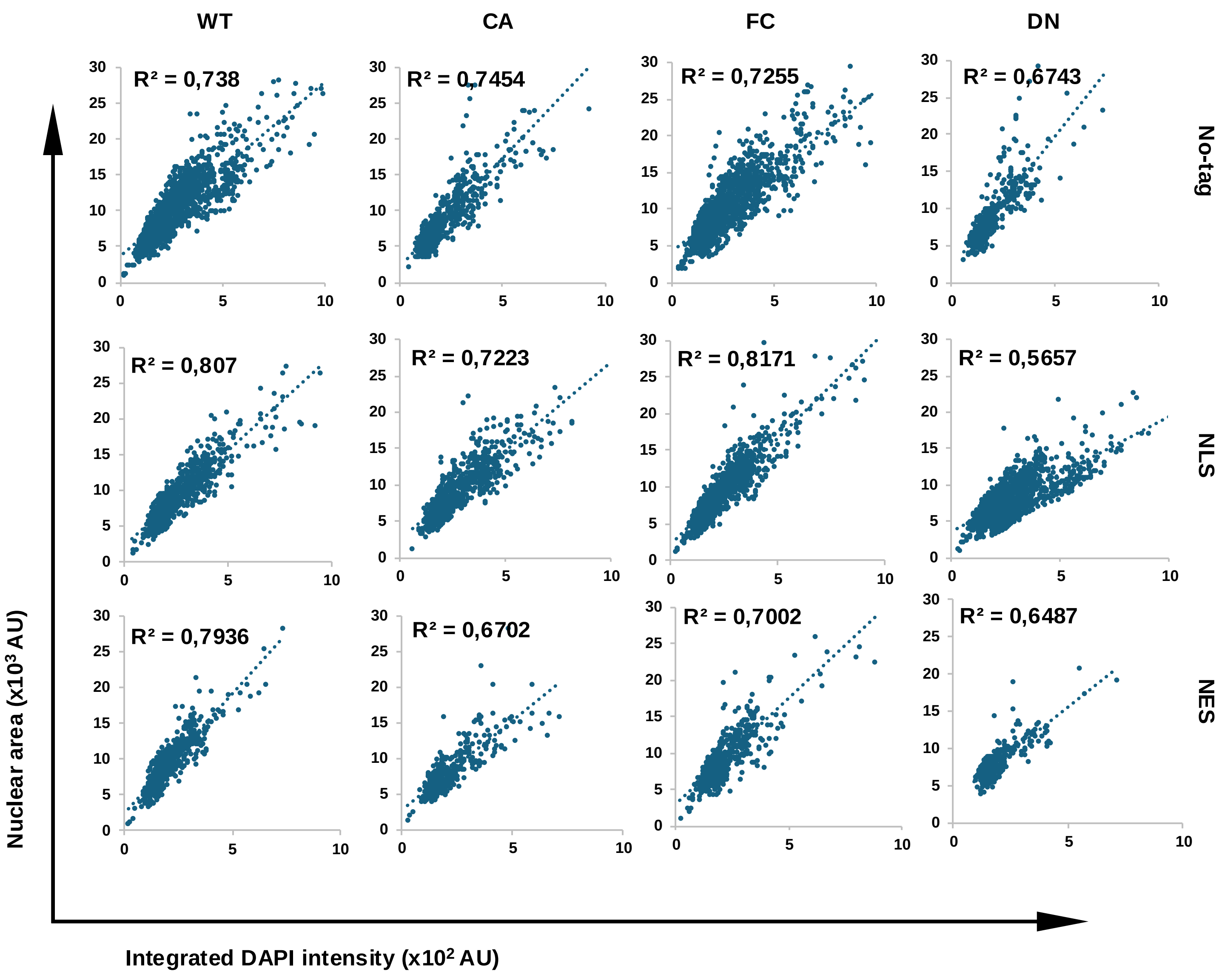

Figure S6: ROCK inhibition decreases cellular F-actin and cell area

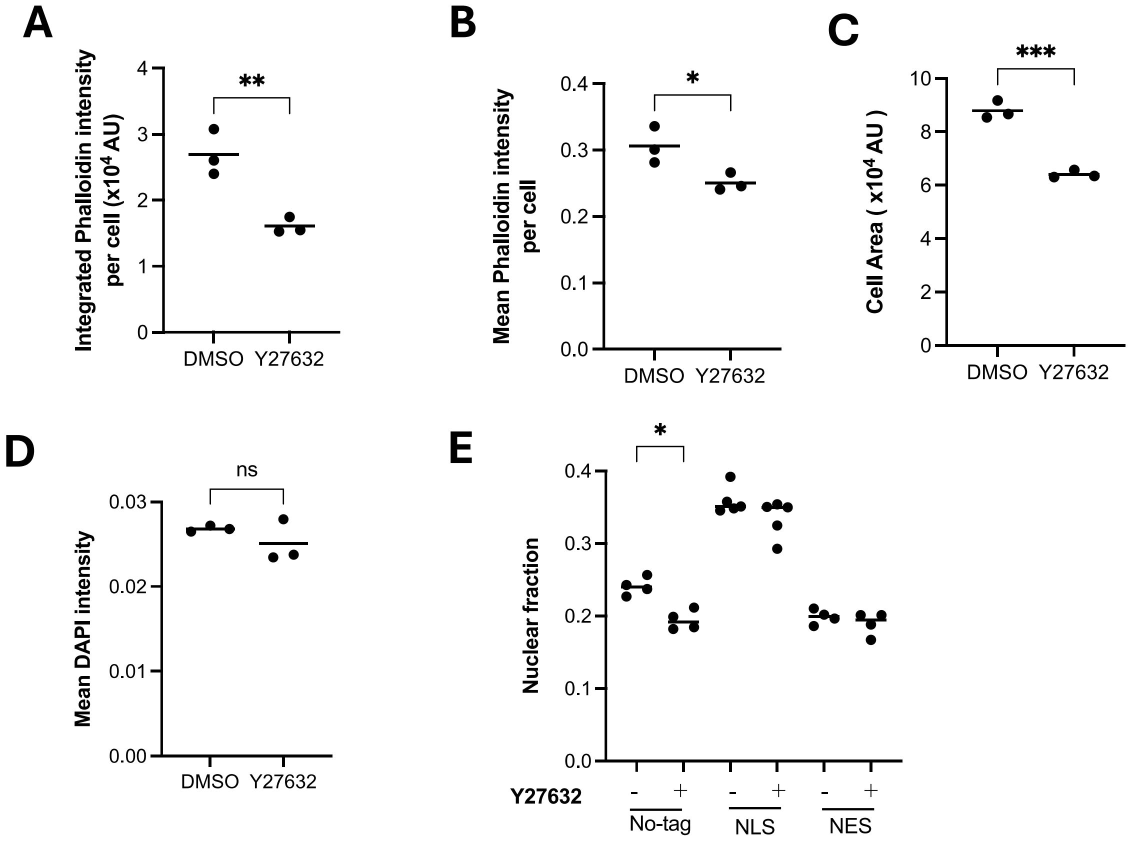

Figure S7: Activated Rac1 and Cdc42 do not efficiently translocate to the nucleus

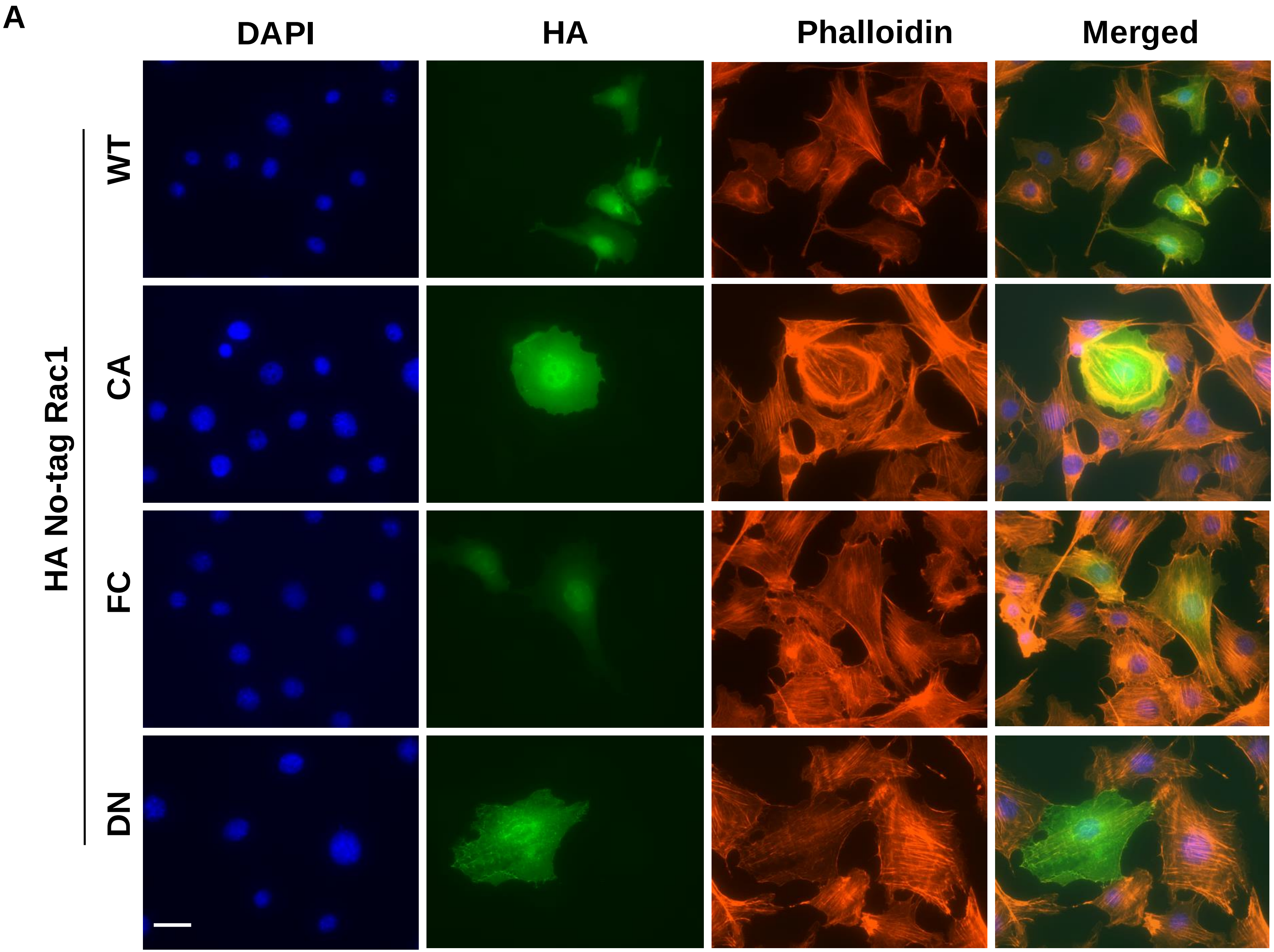

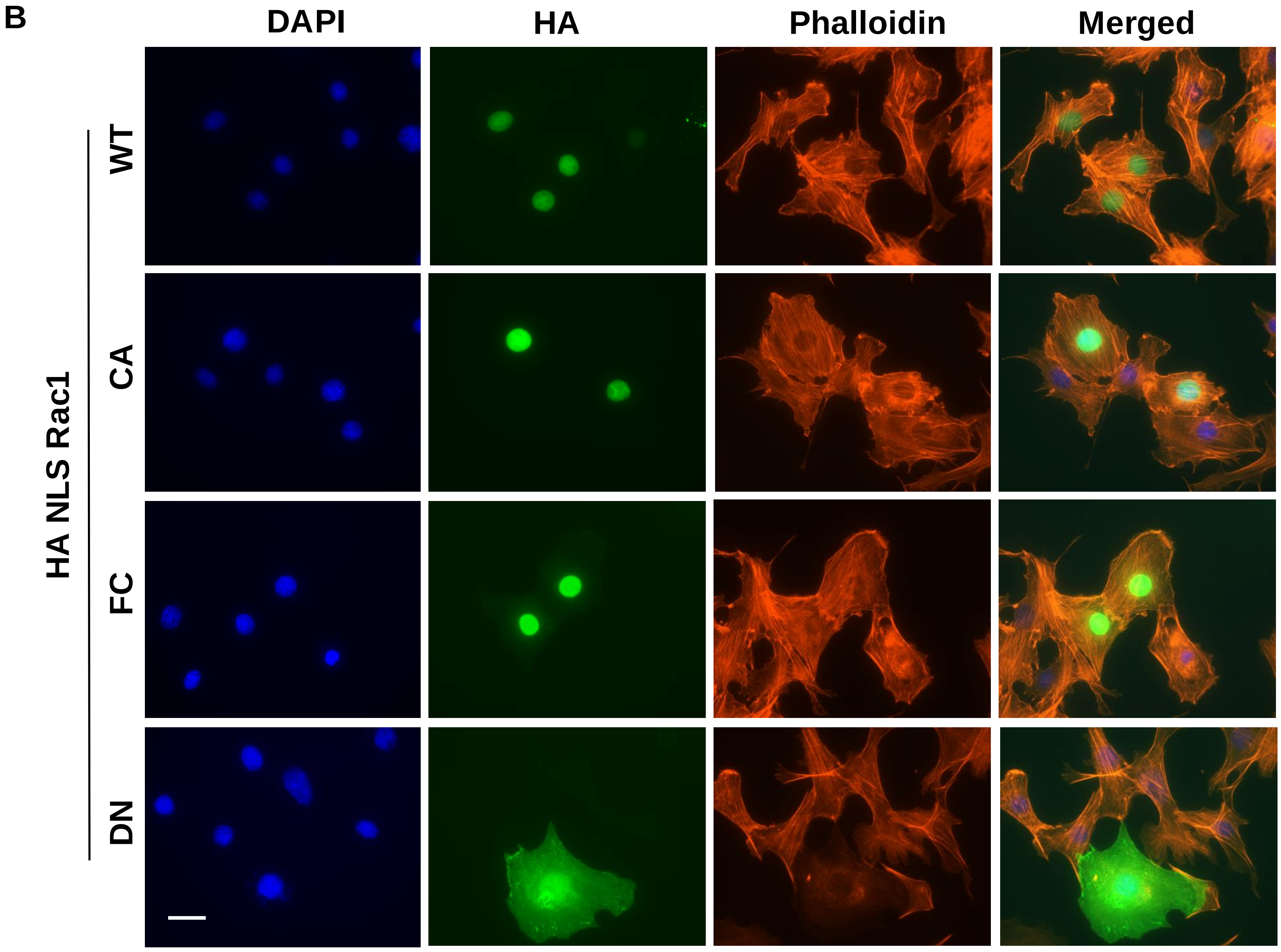
B
DAPI
HA
Phalloidin
Merged
HA NLS Rac1
WT
CA
FC
DN

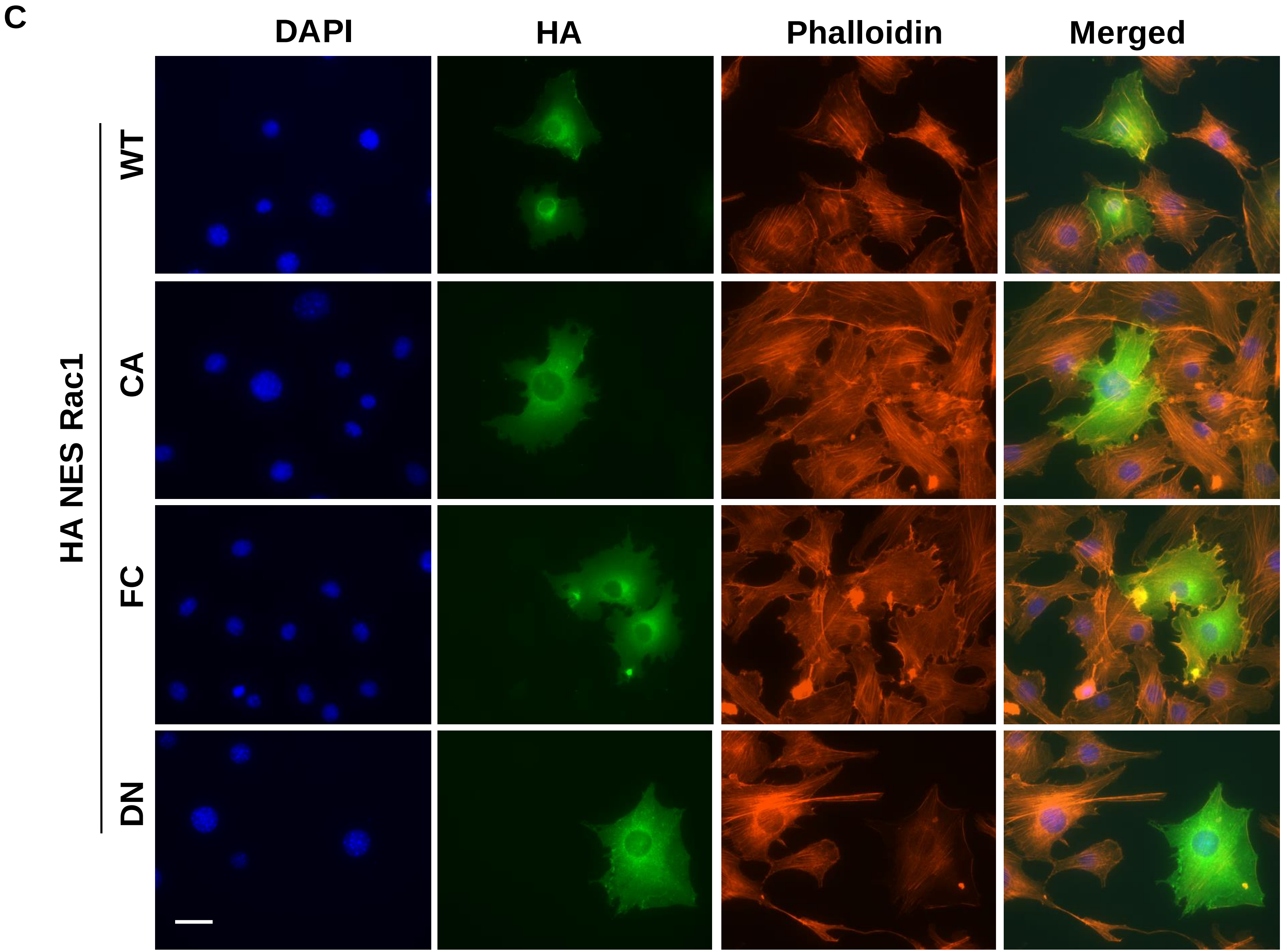
C
DAPI
HA
Phalloidin
Merged
HA NES Rac1
WT
CA
FC
DN

D

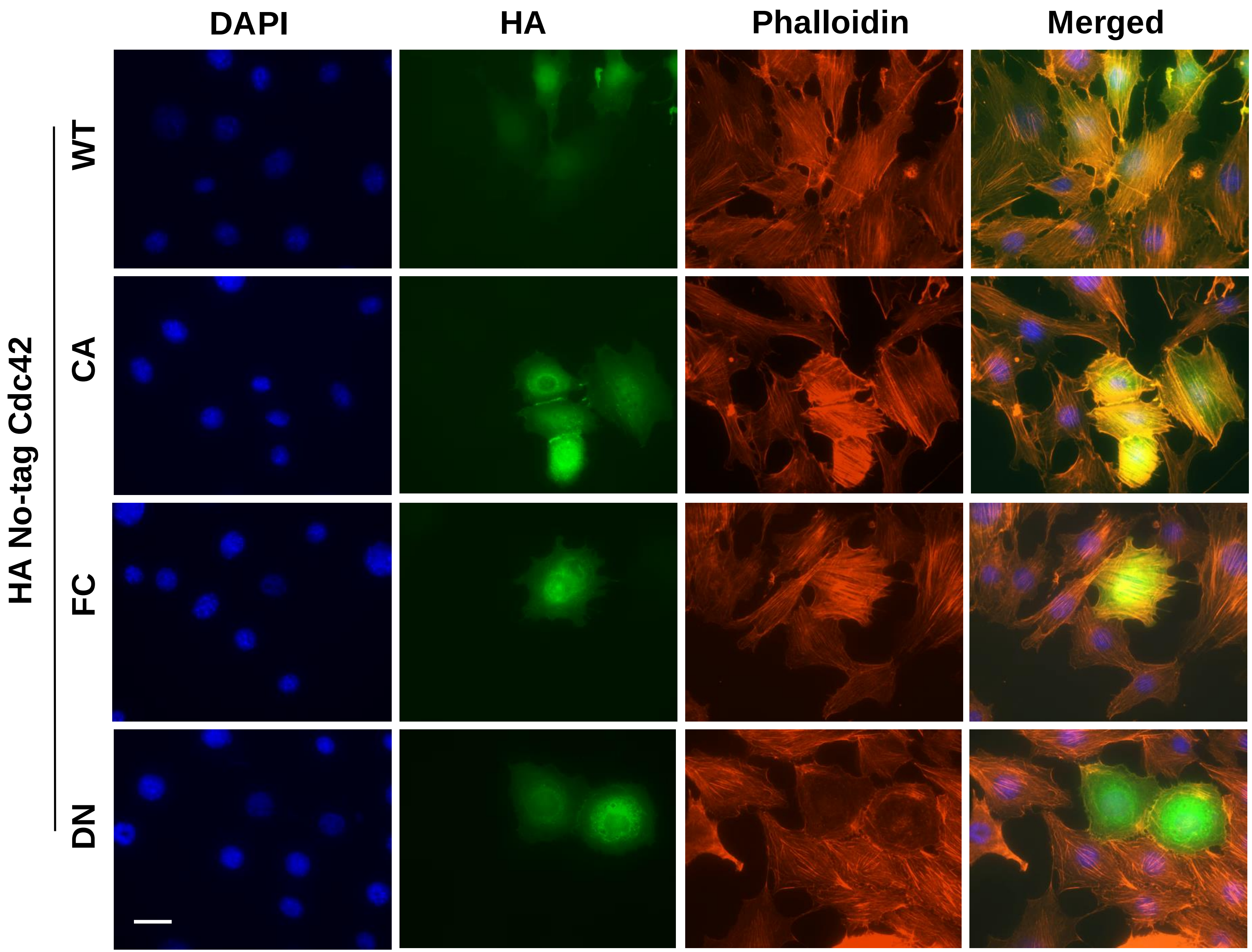

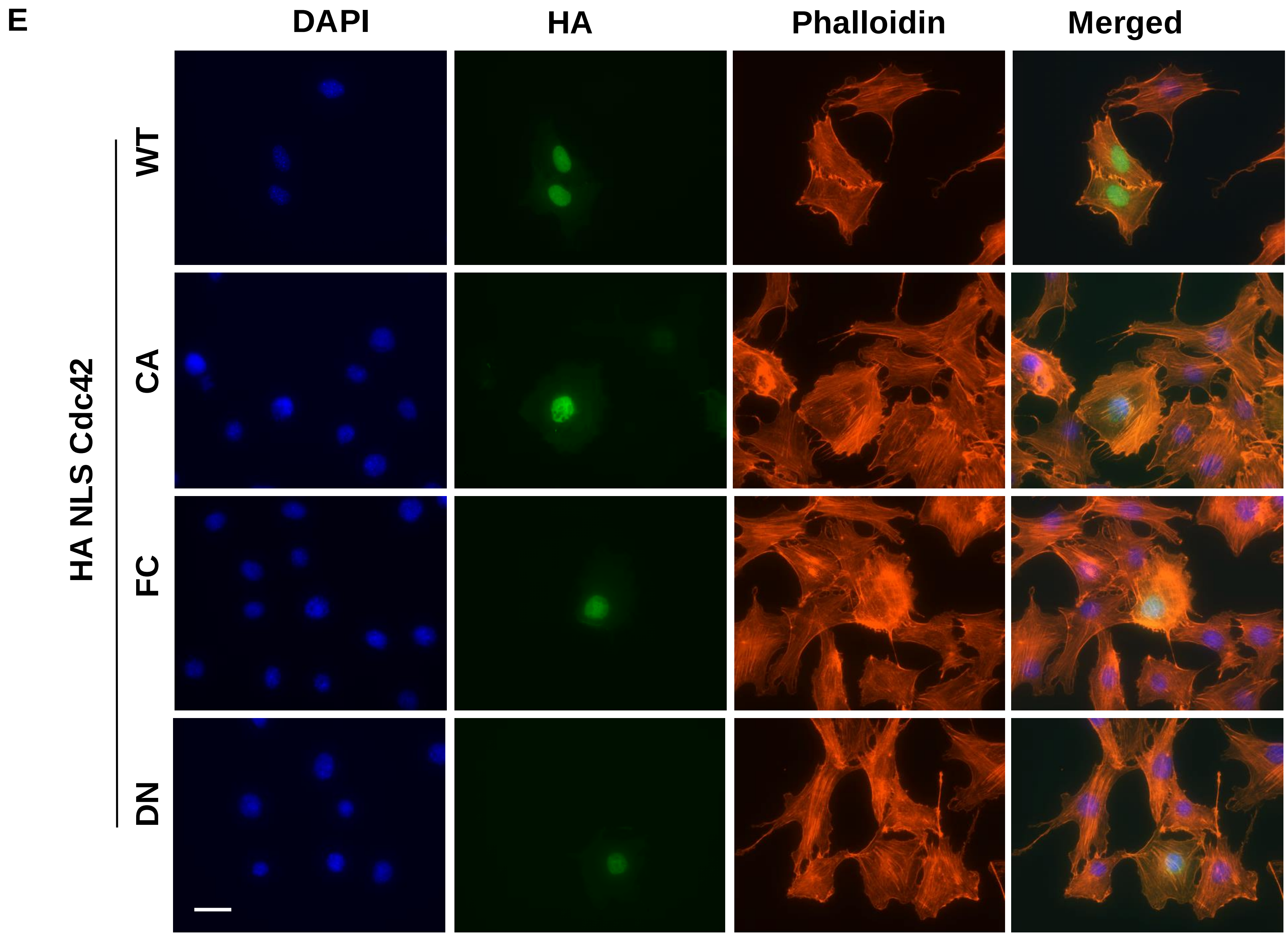
E
DAPI
HA
Phalloidin
Merged
HA NLS Cdc42
WT
CA
FC
DN

F

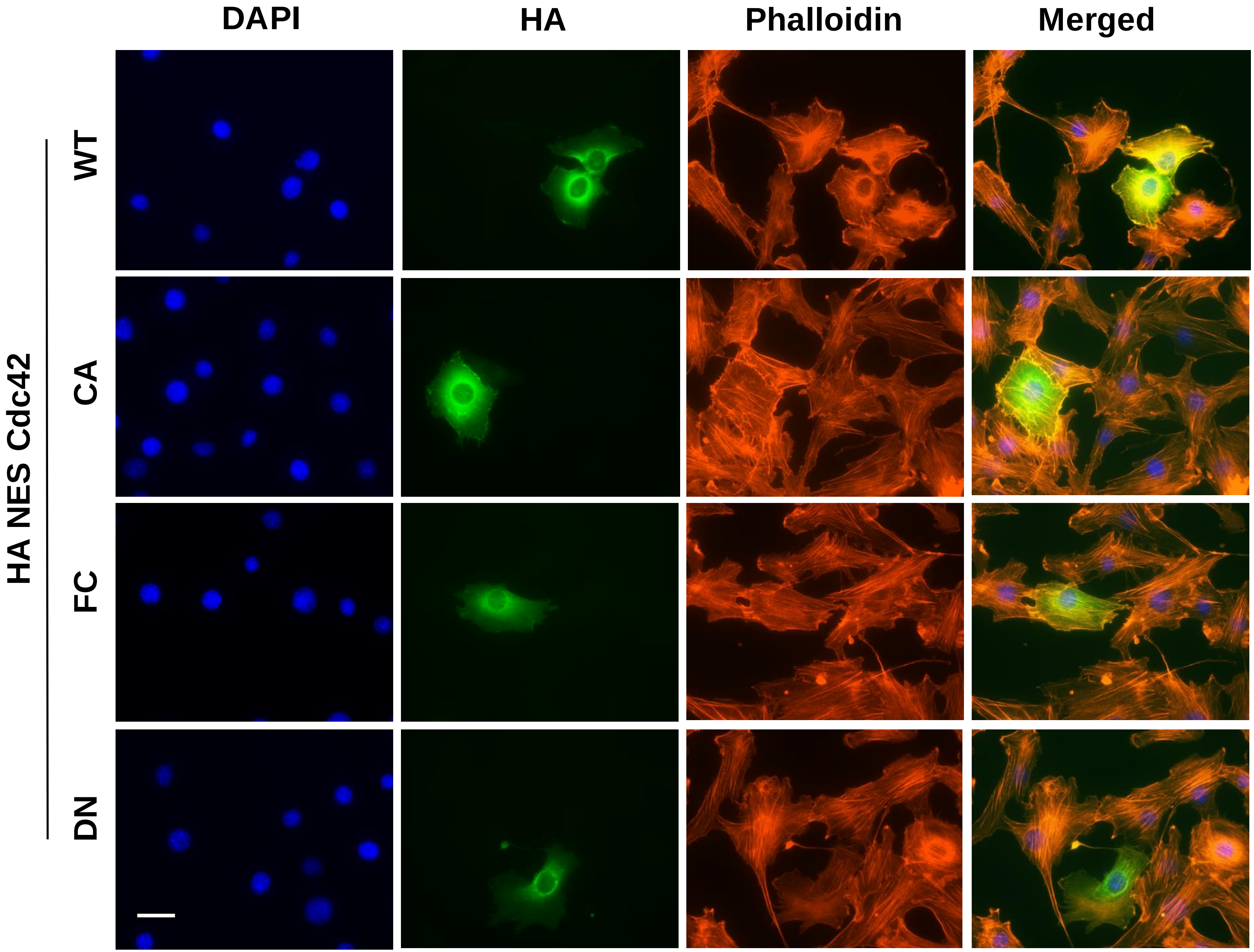

Figure S8: Activated Rac1 and Cdc42 do not efficiently translocate to the nucleus

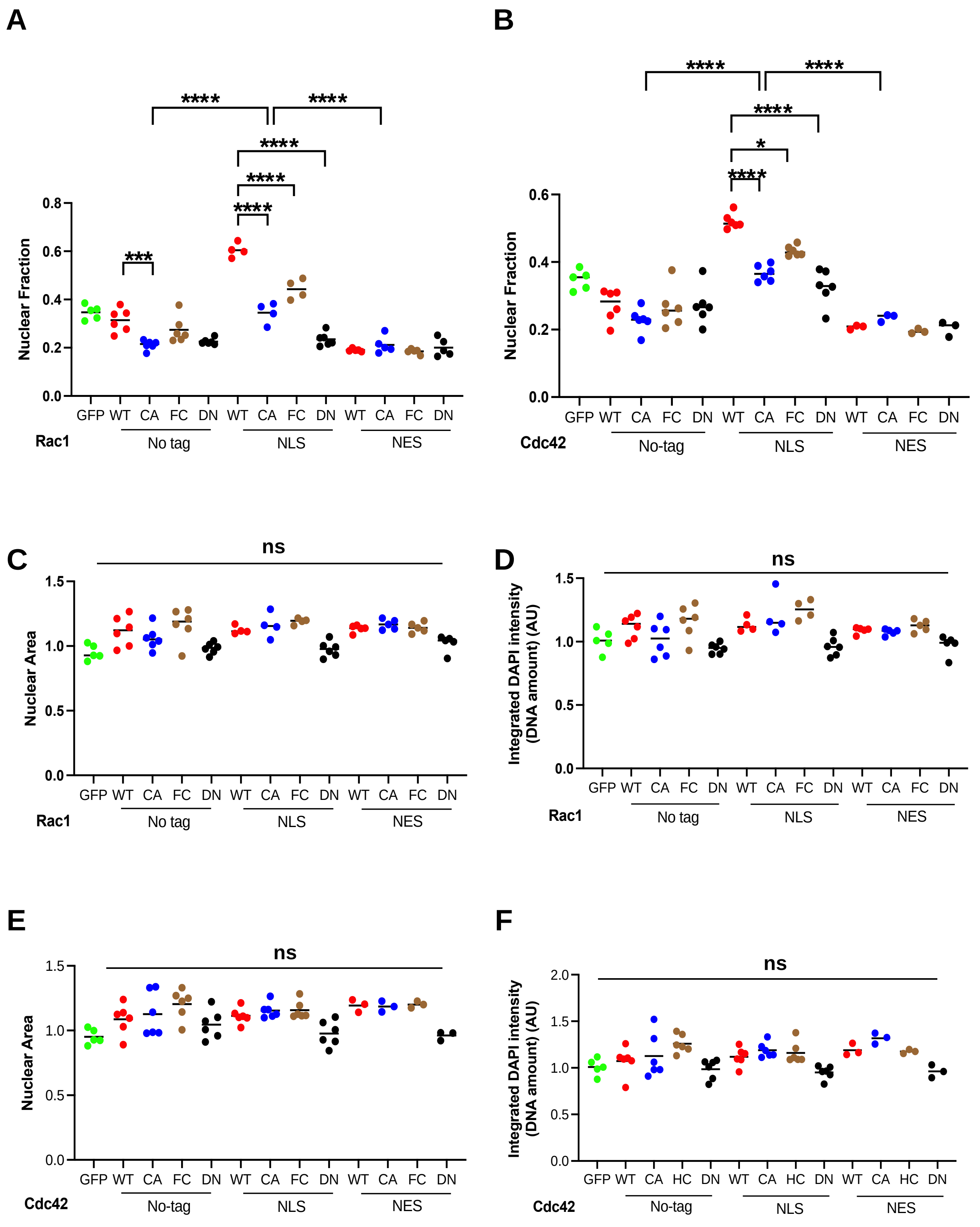

Figure S9: Nuclear RhoA regulates nuclear pErk via ROCK activation

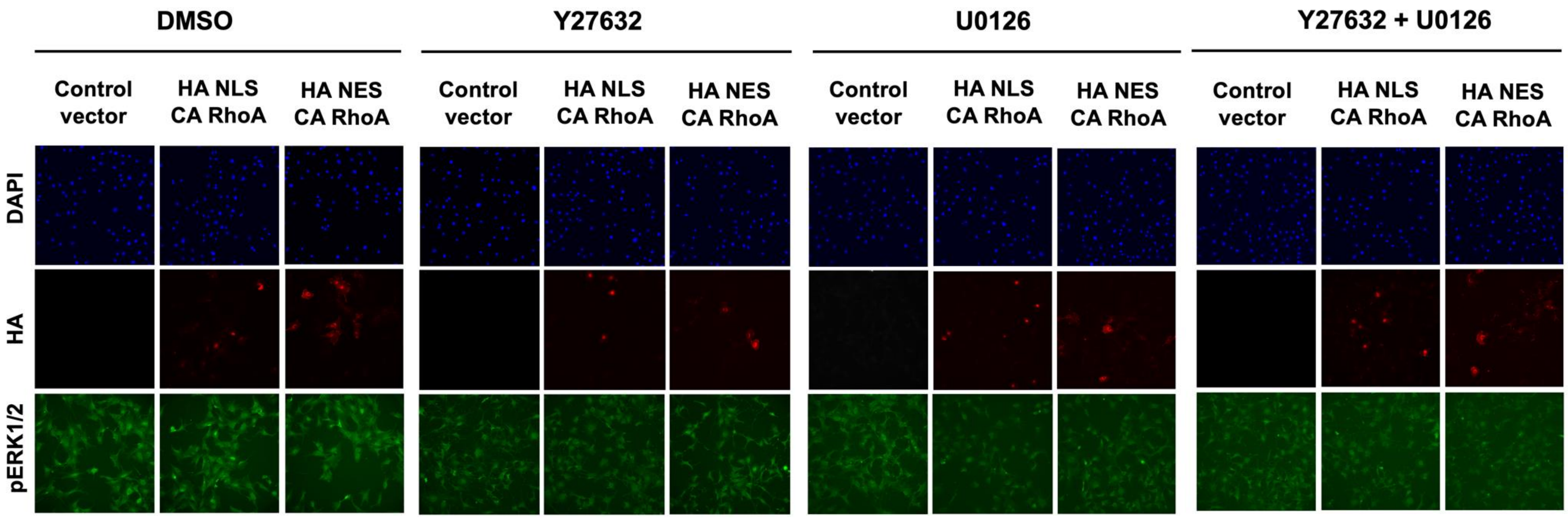

Table S1: Primers used to add HA-. NES-. or NLS-tags to the N-terminus of RhoA of Rac1 and Cdc42

HA RhoA	5’-GCGGGATCCGCCGCCACCATGTACCCATACGATGTTCCAGATTACGCTA TGGCTGCCATCCGG-3’
HA NES RhoA	5’-GCGGGATCCGCCGCCACCATGTACCCATACGATGTTCCAGATTACGCTC TGCCCCTCTGGAGAGACTGACCCTGATGGCTGATCCGG-3
HA NLS RhoA	5’-GCGGGATCCGCCGCCACCATGTACCCATACGATGTTCCAGATTACGCTC CCCTAAGAAAAAGCGGAAAGTGATGGCTGCCATCCGG-3’
Reverse RhoA	5’-GCGGAATTCTCATCATCACAAGACAAGGCACCC-3’
HA Rac1	5’-GCGGGATCCGCCGCCACCATGTACCCATACGATGTTCCAGATTACGCTA TGCAGGCCATCAAGTGT-3’
HA NES Rac1	5’-GCG GGATCCGCCGCCACCATGTACCCATACGATGTTCCAGATTACGCTC TGCCCCCTCTGGAGAGACTGACCCTGATGCAGGCCATCAAGTGT-3’
HA NLS Rac1	5’-GCGGGATCCGCCGCCACCATGTACCCATACGATGTTCCAGATTACGCTC CCCCTAAGAAAAAGCGGAAAGTGATGCAGGCCATCAAGTGT-3’
Reverse Rac1	5’-GCGGAATTCTCATCATTACAACAGCAGGCATTTTC-3’
HA Cdc42	5’-GCGGGATCCGCCGCCACCATGTACCCATACGATGTTCCAGATTACGCTA TGCAGACAATTAAGTGTGTTGTT-3’
HA NES Cdc42	5’-GCGGGATCCGCCGCCACCATGTACCCATACGATGTTCCAGATTACGCTC TGCCCCCTCTGGAGAGAGACCCTGATGCAGACAATTAAGTGTGTTGTT-3’
HA NLS Cdc42	5’-GCGGGATCCGCCGCCACCATGTACCCATACGATGTTCCAGATTACGCTC CCCCTAAGAAAAAGCGGAAAGTGATGCAGACAATTAAGTGTGTTGTT-3’
Reverse Cdc42	5’-GCGGAATTCTCATCATTAGAATATACA GCACTTCCTTTTGGG-3’

Table S2: Modules and settings of the CellProfiler pipeline used for analysis

Module Number & Name	Threshold Smoothing Scale	Threshold Correction Factor	Threshold Bound Lower - Upper	Image	Comment
#1 Identify Primary Object	2.0	1.1	0.001 – 1.0	DAPI	Identifying Nuclei
#2 Identify Secondary Object	6.0	0.9	0.01 – 0.9	Phalloidin	Identifying Cells
#3 Measure Object Intensity	-	-	-	HA-tag	Measuring HA-tag in identified objects
#4 Filter Objects	-	-	-	HA-tag	Filtering for TF nuclei with 25% higher than image background
#5 Filter Objects	-	-	-	HA-tag	Filtering for UTF nuclei, same as minimum value from module #4
#6 Relate Objects	-	-	-	-	Connecting TF nuclei with corresponding cells
#7 Relate Objects	-	-	-	-	Connecting UTF nuclei with corresponding cells
#8 Measure Object Intensity	-	-	-	DAPI<br>HA-Tag<br>Phalloidin	Measuring intensity within defined objects
#9 Measure Object Size and Shape	-	-	-	-	Measuring size and shape within defined objects

Table S3: Tukey's multiple comparisons test and p-value

**Figure 1F_Nuclear fraction**					
**Tukey's multiple comparisons test**	**Mean Diff.**	**95.00% CI of diff.**	**Significant?**	**Summary**	**Adjusted P Value**
GFP vs. Notag WT	0.04256	-0.02061 to 0.1057	No	ns	0.5012
GFP vs. Notag CA	0.1208	0.05761 to 0.1839	Yes	****	<0.0001
GFP vs. Notag FC	0.08052	0.01735 to 0.1437	Yes	**	0.0031
GFP vs. Notag DN	0.076	0.01283 to 0.1392	Yes	**	0.0067
GFP vs. NLS WT	-0.2235	-0.2935 to -0.1536	Yes	****	<0.0001
GFP vs. NLS CA	0.0339	-0.03208 to 0.09987	No	ns	0.8489
GFP vs. NLS FC	-0.1234	-0.1894 to -0.05746	Yes	****	<0.0001
GFP vs. NLS DN	0.001483	-0.06168 to 0.06465	No	ns	>0.9999
GFP vs. NES WT	0.1489	0.07888 to 0.2188	Yes	****	<0.0001
GFP vs. NES CA	0.1421	0.07209 to 0.2120	Yes	****	<0.0001
GFP vs. NES FC	0.1615	0.09154 to 0.2315	Yes	****	<0.0001
GFP vs. NES DN	0.101	0.03104 to 0.1710	Yes	***	0.0004
Notag WT vs. Notag CA	0.07822	0.01799 to 0.1384	Yes	**	0.0024
Notag WT vs. Notag FC	0.03796	-0.02226 to 0.09819	No	ns	0.604
Notag WT vs. Notag DN	0.03345	-0.02678 to 0.09367	No	ns	0.772
Notag WT vs. NLS WT	-0.2661	-0.3334 to -0.1988	Yes	****	<0.0001
Notag WT vs. NLS CA	-0.008661	-0.07183 to 0.05451	No	ns	>0.9999
Notag WT vs. NLS FC	-0.166	-0.2292 to -0.1028	Yes	****	<0.0001
Notag WT vs. NLS DN	-0.04107	-0.1013 to 0.01915	No	ns	0.482
Notag WT vs. NES WT	0.1063	0.03896 to 0.1736	Yes	****	<0.0001
Notag WT vs. NES CA	0.09951	0.03218 to 0.1669	Yes	***	0.0003
Notag WT vs. NES FC	0.119	0.05163 to 0.1863	Yes	****	<0.0001
Notag WT vs. NES DN	0.05846	-0.008873 to 0.1258	No	ns	0.1501
Notag CA vs. Notag FC	-0.04026	-0.1005 to 0.01997	No	ns	0.5136
Notag CA vs. Notag DN	-0.04478	-0.1050 to 0.01545	No	ns	0.3476
Notag CA vs. NLS WT	-0.3443	-0.4117 to -0.2770	Yes	****	<0.0001
Notag CA vs. NLS CA	-0.08688	-0.1501 to -0.02371	Yes	***	0.001
Notag CA vs. NLS FC	-0.2442	-0.3074 to -0.1810	Yes	****	<0.0001
Notag CA vs. NLS DN	-0.1193	-0.1795 to -0.05907	Yes	****	<0.0001
Notag CA vs. NES WT	0.02808	-0.03926 to 0.09542	No	ns	0.9611
Notag CA vs. NES CA	0.02129	-0.04604 to 0.08863	No	ns	0.996
Notag CA vs. NES FC	0.04074	-0.02659 to 0.1081	No	ns	0.6632
Notag CA vs. NES DN	-0.01976	-0.08709 to 0.04758	No	ns	0.998
Notag FC vs. Notag DN	-0.004519	-0.06475 to 0.05571	No	ns	>0.9999
Notag FC vs. NLS WT	-0.3041	-0.3714 to -0.2367	Yes	****	<0.0001
Notag FC vs. NLS CA	-0.04663	-0.1098 to 0.01654	No	ns	0.3584
Notag FC vs. NLS FC	-0.204	-0.2671 to -0.1408	Yes	****	<0.0001
Notag FC vs. NLS DN	-0.07904	-0.1393 to -0.01881	Yes	**	0.002
Notag FC vs. NES WT	0.06834	0.001000 to 0.1357	Yes	*	0.0437
Notag FC vs. NES CA	0.06155	-0.005786 to 0.1289	No	ns	0.1047
Notag FC vs. NES FC	0.081	0.01366 to 0.1483	Yes	**	0.0067

Notag FC vs. NES DN	0.0205	-0.04684 to 0.08784	No	ns	0.9972
Notag DN vs. NLS WT	-0.2995	-0.3669 to -0.2322	Yes	****	<0.0001
Notag DN vs. NLS CA	-0.04211	-0.1053 to 0.02106	No	ns	0.518
Notag DN vs. NLS FC	-0.1994	-0.2626 to -0.1363	Yes	****	<0.0001
Notag DN vs. NLS DN	-0.07452	-0.1347 to -0.01429	Yes	**	0.0046
Notag DN vs. NES WT	0.07286	0.005519 to 0.1402	Yes	*	0.0231
Notag DN vs. NES CA	0.06607	-0.001267 to 0.1334	No	ns	0.0592
Notag DN vs. NES FC	0.08552	0.01818 to 0.1529	Yes	**	0.0032
Notag DN vs. NES DN	0.02502	-0.04232 to 0.09236	No	ns	0.984
NLS WT vs. NLS CA	0.2574	0.1875 to 0.3274	Yes	****	<0.0001
NLS WT vs. NLS FC	0.1001	0.03013 to 0.1701	Yes	***	0.0005
NLS WT vs. NLS DN	0.225	0.1577 to 0.2924	Yes	****	<0.0001
NLS WT vs. NES WT	0.3724	0.2986 to 0.4462	Yes	****	<0.0001
NLS WT vs. NES CA	0.3656	0.2918 to 0.4394	Yes	****	<0.0001
NLS WT vs. NES FC	0.3851	0.3113 to 0.4588	Yes	****	<0.0001
NLS WT vs. NES DN	0.3246	0.2508 to 0.3983	Yes	****	<0.0001
NLS CA vs. NLS FC	-0.1573	-0.2233 to -0.09136	Yes	****	<0.0001
NLS CA vs. NLS DN	-0.03241	-0.09558 to 0.03076	No	ns	0.8499
NLS CA vs. NES WT	0.115	0.04498 to 0.1849	Yes	****	<0.0001
NLS CA vs. NES CA	0.1082	0.03820 to 0.1782	Yes	***	0.0001
NLS CA vs. NES FC	0.1276	0.05765 to 0.1976	Yes	****	<0.0001
NLS CA vs. NES DN	0.06713	-0.002853 to 0.1371	No	ns	0.0717
NLS FC vs. NLS DN	0.1249	0.06175 to 0.1881	Yes	****	<0.0001
NLS FC vs. NES WT	0.2723	0.2023 to 0.3423	Yes	****	<0.0001
NLS FC vs. NES CA	0.2655	0.1955 to 0.3355	Yes	****	<0.0001
NLS FC vs. NES FC	0.285	0.2150 to 0.3549	Yes	****	<0.0001
NLS FC vs. NES DN	0.2245	0.1545 to 0.2944	Yes	****	<0.0001
NLS DN vs. NES WT	0.1474	0.08004 to 0.2147	Yes	****	<0.0001
NLS DN vs. NES CA	0.1406	0.07325 to 0.2079	Yes	****	<0.0001
NLS DN vs. NES FC	0.16	0.09270 to 0.2274	Yes	****	<0.0001
NLS DN vs. NES DN	0.09954	0.03220 to 0.1669	Yes	***	0.0003
NES WT vs. NES CA	-0.006786	-0.08055 to 0.06698	No	ns	>0.9999
NES WT vs. NES FC	0.01266	-0.06110 to 0.08643	No	ns	>0.9999
NES WT vs. NES DN	-0.04784	-0.1216 to 0.02593	No	ns	0.5608
NES CA vs. NES FC	0.01945	-0.05431 to 0.09321	No	ns	0.9993
NES CA vs. NES DN	-0.04105	-0.1148 to 0.03271	No	ns	0.7695
NES FC vs. NES DN	-0.0605	-0.1343 to 0.01326	No	ns	0.2129

Figure 2A_Integrated cellular phalloidin					
**Tukey's multiple comparisons test**	**Mean Diff.**	**95.00% CI of diff.**	**Significant?**	**Summary**	**Adjusted P Value**
GFP vs. Notag WT	-0.2824	-0.5979 to 0.03307	No	ns	0.1219
GFP vs. Notag CA	-1.071	-1.387 to -0.7556	Yes	****	<0.0001
GFP vs. Notag FC	-0.6314	-0.9468 to -0.3159	Yes	****	<0.0001
GFP vs. Notag DN	0.2366	-0.07889 to 0.5521	No	ns	0.3346
GFP vs. NLS WT	-0.3012	-0.6507 to 0.04829	No	ns	0.1575
GFP vs. NLS CA	-0.7589	-1.088 to -0.4293	Yes	****	<0.0001
GFP vs. NLS FC	-0.369	-0.6985 to -0.03952	Yes	*	0.0158
GFP vs. NLS DN	0.2075	-0.1080 to 0.5230	No	ns	0.539
GFP vs. NES WT	-0.4006	-0.7501 to -0.05115	Yes	*	0.0121
GFP vs. NES CA	-0.7866	-1.136 to -0.4371	Yes	****	<0.0001
GFP vs. NES FC	-0.422	-0.7715 to -0.07250	Yes	**	0.0064
GFP vs. NES DN	0.09051	-0.2590 to 0.4400	No	ns	0.9994
Notag WT vs. Notag CA	-0.7887	-1.089 to -0.4879	Yes	****	<0.0001
Notag WT vs. Notag FC	-0.349	-0.6498 to -0.04816	Yes	*	0.0105
Notag WT vs. Notag DN	0.519	0.2182 to 0.8198	Yes	****	<0.0001
Notag WT vs. NLS WT	-0.0188	-0.3551 to 0.3175	No	ns	>0.9999
Notag WT vs. NLS CA	-0.4764	-0.7919 to -0.1610	Yes	***	0.0002
Notag WT vs. NLS FC	-0.08662	-0.4021 to 0.2289	No	ns	0.9989
Notag WT vs. NLS DN	0.4899	0.1891 to 0.7907	Yes	****	<0.0001
Notag WT vs. NES WT	-0.1182	-0.4545 to 0.2181	No	ns	0.9899
Notag WT vs. NES CA	-0.5042	-0.8405 to -0.1679	Yes	***	0.0002
Notag WT vs. NES FC	-0.1396	-0.4759 to 0.1967	No	ns	0.9624
Notag WT vs. NES DN	0.3729	0.03661 to 0.7092	Yes	*	0.0177
Notag CA vs. Notag FC	0.4397	0.1389 to 0.7405	Yes	***	0.0004
Notag CA vs. Notag DN	1.308	1.007 to 1.608	Yes	****	<0.0001
Notag CA vs. NLS WT	0.7699	0.4336 to 1.106	Yes	****	<0.0001
Notag CA vs. NLS CA	0.3122	-0.003234 to 0.6277	No	ns	0.0548
Notag CA vs. NLS FC	0.7021	0.3866 to 1.018	Yes	****	<0.0001
Notag CA vs. NLS DN	1.279	0.9778 to 1.579	Yes	****	<0.0001
Notag CA vs. NES WT	0.6705	0.3342 to 1.007	Yes	****	<0.0001
Notag CA vs. NES CA	0.2845	-0.05178 to 0.6208	No	ns	0.1771
Notag CA vs. NES FC	0.6491	0.3128 to 0.9854	Yes	****	<0.0001
Notag CA vs. NES DN	1.162	0.8253 to 1.498	Yes	****	<0.0001
Notag FC vs. Notag DN	0.868	0.5672 to 1.169	Yes	****	<0.0001
Notag FC vs. NLS WT	0.3302	-0.006147 to 0.6665	No	ns	0.0589
Notag FC vs. NLS CA	-0.1275	-0.4430 to 0.1880	No	ns	0.9692
Notag FC vs. NLS FC	0.2623	-0.05315 to 0.5778	No	ns	0.1966
Notag FC vs. NLS DN	0.8388	0.5380 to 1.140	Yes	****	<0.0001
Notag FC vs. NES WT	0.2307	-0.1056 to 0.5670	No	ns	0.4725
Notag FC vs. NES CA	-0.1552	-0.4915 to 0.1811	No	ns	0.9213
Notag FC vs. NES FC	0.2094	-0.1269 to 0.5457	No	ns	0.6224
Notag FC vs. NES DN	0.7219	0.3856 to 1.058	Yes	****	<0.0001
Notag DN vs. NLS WT	-0.5378	-0.8741 to -0.2015	Yes	****	<0.0001
Notag DN vs. NLS CA	-0.9954	-1.311 to -0.6800	Yes	****	<0.0001

Notag DN vs. NLS FC	-0.6056	-0.9211 to -0.2901	Yes	****	<0.0001
Notag DN vs. NLS DN	-0.02911	-0.3299 to 0.2717	No	ns	>0.9999
Notag DN vs. NES WT	-0.6372	-0.9735 to -0.3009	Yes	****	<0.0001
Notag DN vs. NES CA	-1.023	-1.359 to -0.6869	Yes	****	<0.0001
Notag DN vs. NES FC	-0.6586	-0.9949 to -0.3223	Yes	****	<0.0001
Notag DN vs. NES DN	-0.1461	-0.4824 to 0.1902	No	ns	0.9478
NLS WT vs. NLS CA	-0.4576	-0.8071 to -0.1081	Yes	**	0.0021
NLS WT vs. NLS FC	-0.06782	-0.4173 to 0.2817	No	ns	>0.9999
NLS WT vs. NLS DN	0.5087	0.1724 to 0.8450	Yes	***	0.0002
NLS WT vs. NES WT	-0.09943	-0.4678 to 0.2690	No	ns	0.9991
NLS WT vs. NES CA	-0.4854	-0.8538 to -0.1170	Yes	**	0.0019
NLS WT vs. NES FC	-0.1208	-0.4892 to 0.2476	No	ns	0.9945
NLS WT vs. NES DN	0.3917	0.02331 to 0.7601	Yes	*	0.0277
NLS CA vs. NLS FC	0.3898	0.06032 to 0.7193	Yes	**	0.0083
NLS CA vs. NLS DN	0.9663	0.6509 to 1.282	Yes	****	<0.0001
NLS CA vs. NES WT	0.3582	0.008717 to 0.7077	Yes	*	0.0398
NLS CA vs. NES CA	-0.02773	-0.3772 to 0.3218	No	ns	>0.9999
NLS CA vs. NES FC	0.3369	-0.01264 to 0.6863	No	ns	0.0689
NLS CA vs. NES DN	0.8494	0.4999 to 1.199	Yes	****	<0.0001
NLS FC vs. NLS DN	0.5765	0.2610 to 0.8920	Yes	****	<0.0001
NLS FC vs. NES WT	-0.03161	-0.3811 to 0.3179	No	ns	>0.9999
NLS FC vs. NES CA	-0.4175	-0.7670 to -0.06805	Yes	**	0.0073
NLS FC vs. NES FC	-0.05297	-0.4025 to 0.2965	No	ns	>0.9999
NLS FC vs. NES DN	0.4595	0.1100 to 0.8090	Yes	**	0.002
NLS DN vs. NES WT	-0.6081	-0.9444 to -0.2718	Yes	****	<0.0001
NLS DN vs. NES CA	-0.9941	-1.330 to -0.6578	Yes	****	<0.0001
NLS DN vs. NES FC	-0.6295	-0.9658 to -0.2932	Yes	****	<0.0001
NLS DN vs. NES DN	-0.117	-0.4533 to 0.2193	No	ns	0.9908
NES WT vs. NES CA	-0.3859	-0.7543 to -0.01754	Yes	*	0.0322
NES WT vs. NES FC	-0.02136	-0.3898 to 0.3470	No	ns	>0.9999
NES WT vs. NES DN	0.4911	0.1227 to 0.8595	Yes	**	0.0016
NES CA vs. NES FC	0.3646	-0.003820 to 0.7330	No	ns	0.0549
NES CA vs. NES DN	0.8771	0.5087 to 1.245	Yes	****	<0.0001
NES FC vs. NES DN	0.5125	0.1441 to 0.8809	Yes	***	0.0008

**Figure 2B_Mean cellular phalloidin**					
**Tukey's multiple comparisons test**	**Mean Diff.**	**95.00% CI of diff.**	**Significant?**	**Summary**	**Adjusted P Value**
GFP vs. Notag WT	-0.01386	-0.1088 to 0.08112	No	ns	>0.9999
GFP vs. Notag CA	-0.3999	-0.4949 to -0.3049	Yes	****	<0.0001
GFP vs. Notag FC	-0.07776	-0.1727 to 0.01723	No	ns	0.2152
GFP vs. Notag DN	0.2898	0.1948 to 0.3848	Yes	****	<0.0001
GFP vs. NLS WT	-0.06792	-0.1731 to 0.03731	No	ns	0.5681
GFP vs. NLS CA	-0.253	-0.3522 to -0.1538	Yes	****	<0.0001
GFP vs. NLS FC	-0.1336	-0.2328 to -0.03438	Yes	**	0.0014
GFP vs. NLS DN	0.132	0.03700 to 0.2270	Yes	***	0.0008
GFP vs. NES WT	-0.05879	-0.1640 to 0.04644	No	ns	0.7651
GFP vs. NES CA	-0.2871	-0.3924 to -0.1819	Yes	****	<0.0001
GFP vs. NES FC	-0.08147	-0.1867 to 0.02376	No	ns	0.2886
GFP vs. NES DN	0.1407	0.03550 to 0.2460	Yes	**	0.0015
Notag WT vs. Notag CA	-0.386	-0.4766 to -0.2954	Yes	****	<0.0001
Notag WT vs. Notag FC	-0.0639	-0.1545 to 0.02667	No	ns	0.4283
Notag WT vs. Notag DN	0.3036	0.2131 to 0.3942	Yes	****	<0.0001
Notag WT vs. NLS WT	-0.05405	-0.1553 to 0.04720	No	ns	0.8136
Notag WT vs. NLS CA	-0.2391	-0.3341 to -0.1441	Yes	****	<0.0001
Notag WT vs. NLS FC	-0.1197	-0.2147 to -0.02475	Yes	**	0.0036
Notag WT vs. NLS DN	0.1458	0.05528 to 0.2364	Yes	****	<0.0001
Notag WT vs. NES WT	-0.04493	-0.1462 to 0.05633	No	ns	0.9395
Notag WT vs. NES CA	-0.2733	-0.3745 to -0.1720	Yes	****	<0.0001
Notag WT vs. NES FC	-0.06761	-0.1689 to 0.03365	No	ns	0.5153
Notag WT vs. NES DN	0.1546	0.05333 to 0.2558	Yes	***	0.0002
Notag CA vs. Notag FC	0.3221	0.2315 to 0.4127	Yes	****	<0.0001
Notag CA vs. Notag DN	0.6896	0.5991 to 0.7802	Yes	****	<0.0001
Notag CA vs. NLS WT	0.332	0.2307 to 0.4332	Yes	****	<0.0001
Notag CA vs. NLS CA	0.1469	0.05190 to 0.2419	Yes	***	0.0001
Notag CA vs. NLS FC	0.2663	0.1713 to 0.3613	Yes	****	<0.0001
Notag CA vs. NLS DN	0.5319	0.4413 to 0.6224	Yes	****	<0.0001
Notag CA vs. NES WT	0.3411	0.2398 to 0.4423	Yes	****	<0.0001
Notag CA vs. NES CA	0.1127	0.01148 to 0.2140	Yes	*	0.0169
Notag CA vs. NES FC	0.3184	0.2171 to 0.4197	Yes	****	<0.0001
Notag CA vs. NES DN	0.5406	0.4393 to 0.6419	Yes	****	<0.0001
Notag FC vs. Notag DN	0.3675	0.2770 to 0.4581	Yes	****	<0.0001
Notag FC vs. NLS WT	0.009844	-0.09141 to 0.1111	No	ns	>0.9999
Notag FC vs. NLS CA	-0.1752	-0.2702 to -0.08024	Yes	****	<0.0001
Notag FC vs. NLS FC	-0.05583	-0.1508 to 0.03915	No	ns	0.7025
Notag FC vs. NLS DN	0.2097	0.1192 to 0.3003	Yes	****	<0.0001
Notag FC vs. NES WT	0.01897	-0.08229 to 0.1202	No	ns	>0.9999
Notag FC vs. NES CA	-0.2094	-0.3106 to -0.1081	Yes	****	<0.0001
Notag FC vs. NES FC	-0.003709	-0.1050 to 0.09755	No	ns	>0.9999
Notag FC vs. NES DN	0.2185	0.1172 to 0.3197	Yes	****	<0.0001
Notag DN vs. NLS WT	-0.3577	-0.4589 to -0.2564	Yes	****	<0.0001
Notag DN vs. NLS CA	-0.5428	-0.6377 to -0.4478	Yes	****	<0.0001

Notag DN vs. NLS FC	-0.4234	-0.5183 to -0.3284	Yes	****	<0.0001
Notag DN vs. NLS DN	-0.1578	-0.2483 to -0.06722	Yes	****	<0.0001
Notag DN vs. NES WT	-0.3486	-0.4498 to -0.2473	Yes	****	<0.0001
Notag DN vs. NES CA	-0.5769	-0.6782 to -0.4757	Yes	****	<0.0001
Notag DN vs. NES FC	-0.3712	-0.4725 to -0.2700	Yes	****	<0.0001
Notag DN vs. NES DN	-0.149	-0.2503 to -0.04779	Yes	***	0.0003
NLS WT vs. NLS CA	-0.1851	-0.2903 to -0.07984	Yes	****	<0.0001
NLS WT vs. NLS FC	-0.06568	-0.1709 to 0.03955	No	ns	0.6186
NLS WT vs. NLS DN	0.1999	0.09865 to 0.3012	Yes	****	<0.0001
NLS WT vs. NES WT	0.009125	-0.1018 to 0.1200	No	ns	>0.9999
NLS WT vs. NES CA	-0.2192	-0.3301 to -0.1083	Yes	****	<0.0001
NLS WT vs. NES FC	-0.01355	-0.1245 to 0.09737	No	ns	>0.9999
NLS WT vs. NES DN	0.2086	0.09772 to 0.3196	Yes	****	<0.0001
NLS CA vs. NLS FC	0.1194	0.02018 to 0.2186	Yes	**	0.0067
NLS CA vs. NLS DN	0.385	0.2900 to 0.4800	Yes	****	<0.0001
NLS CA vs. NES WT	0.1942	0.08896 to 0.2994	Yes	****	<0.0001
NLS CA vs. NES CA	-0.03416	-0.1394 to 0.07107	No	ns	0.9949
NLS CA vs. NES FC	0.1715	0.06629 to 0.2767	Yes	****	<0.0001
NLS CA vs. NES DN	0.3937	0.2885 to 0.4989	Yes	****	<0.0001
NLS FC vs. NLS DN	0.2656	0.1706 to 0.3606	Yes	****	<0.0001
NLS FC vs. NES WT	0.0748	-0.03042 to 0.1800	No	ns	0.4165
NLS FC vs. NES CA	-0.1535	-0.2588 to -0.04832	Yes	***	0.0004
NLS FC vs. NES FC	0.05213	-0.05310 to 0.1574	No	ns	0.8776
NLS FC vs. NES DN	0.2743	0.1691 to 0.3795	Yes	****	<0.0001
NLS DN vs. NES WT	-0.1908	-0.2920 to -0.08952	Yes	****	<0.0001
NLS DN vs. NES CA	-0.4191	-0.5204 to -0.3179	Yes	****	<0.0001
NLS DN vs. NES FC	-0.2135	-0.3147 to -0.1122	Yes	****	<0.0001
NLS DN vs. NES DN	0.008735	-0.09252 to 0.1100	No	ns	>0.9999
NES WT vs. NES CA	-0.2284	-0.3393 to -0.1174	Yes	****	<0.0001
NES WT vs. NES FC	-0.02268	-0.1336 to 0.08824	No	ns	>0.9999
NES WT vs. NES DN	0.1995	0.08859 to 0.3104	Yes	****	<0.0001
NES CA vs. NES FC	0.2057	0.09475 to 0.3166	Yes	****	<0.0001
NES CA vs. NES DN	0.4279	0.3169 to 0.5388	Yes	****	<0.0001
NES FC vs. NES DN	0.2222	0.1113 to 0.3331	Yes	****	<0.0001

Figure 2C_Cell Area					
**Tukey's multiple comparisons test**	**Mean Diff.**	**95.00% CI of diff.**	**Significant?**	**Summary**	**Adjusted P Value**
GFP vs. Notag WT	-0.2549	-0.5444 to 0.03460	No	ns	0.1367
GFP vs. Notag CA	-0.4986	-0.7881 to -0.2090	Yes	****	<0.0001
GFP vs. Notag FC	-0.5041	-0.7936 to -0.2146	Yes	****	<0.0001
GFP vs. Notag DN	-0.04146	-0.3310 to 0.2481	No	ns	>0.9999
GFP vs. NLS WT	-0.2174	-0.5381 to 0.1033	No	ns	0.4916
GFP vs. NLS CA	-0.3653	-0.6677 to -0.06288	Yes	**	0.0064
GFP vs. NLS FC	-0.1959	-0.4983 to 0.1065	No	ns	0.5626
GFP vs. NLS DN	0.08528	-0.2042 to 0.3748	No	ns	0.9979
GFP vs. NES WT	-0.3366	-0.6573 to -0.01585	Yes	*	0.0316
GFP vs. NES CA	-0.3576	-0.6783 to -0.03686	Yes	*	0.0167
GFP vs. NES FC	-0.3171	-0.6378 to 0.003639	No	ns	0.0554
GFP vs. NES DN	-0.1048	-0.4255 to 0.2159	No	ns	0.9946
Notag WT vs. Notag CA	-0.2436	-0.5197 to 0.03239	No	ns	0.1344
Notag WT vs. Notag FC	-0.2492	-0.5252 to 0.02683	No	ns	0.1147
Notag WT vs. Notag DN	0.2135	-0.06259 to 0.4895	No	ns	0.2903
Notag WT vs. NLS WT	0.03751	-0.2711 to 0.3461	No	ns	>0.9999
Notag WT vs. NLS CA	-0.1104	-0.3999 to 0.1792	No	ns	0.9804
Notag WT vs. NLS FC	0.05905	-0.2305 to 0.3486	No	ns	>0.9999
Notag WT vs. NLS DN	0.3402	0.06415 to 0.6162	Yes	**	0.0049
Notag WT vs. NES WT	-0.08167	-0.3903 to 0.2270	No	ns	0.9993
Notag WT vs. NES CA	-0.1027	-0.4113 to 0.2059	No	ns	0.9937
Notag WT vs. NES FC	-0.06218	-0.3708 to 0.2464	No	ns	>0.9999
Notag WT vs. NES DN	0.1501	-0.1585 to 0.4587	No	ns	0.8904
Notag CA vs. Notag FC	-0.005562	-0.2816 to 0.2705	No	ns	>0.9999
Notag CA vs. Notag DN	0.4571	0.1811 to 0.7331	Yes	****	<0.0001
Notag CA vs. NLS WT	0.2812	-0.02747 to 0.5898	No	ns	0.1074
Notag CA vs. NLS CA	0.1333	-0.1562 to 0.4228	No	ns	0.9226
Notag CA vs. NLS FC	0.3027	0.01318 to 0.5922	Yes	*	0.0328
Notag CA vs. NLS DN	0.5838	0.3078 to 0.8599	Yes	****	<0.0001
Notag CA vs. NES WT	0.162	-0.1466 to 0.4706	No	ns	0.8299
Notag CA vs. NES CA	0.141	-0.1677 to 0.4496	No	ns	0.9265
Notag CA vs. NES FC	0.1815	-0.1272 to 0.4901	No	ns	0.7021
Notag CA vs. NES DN	0.3938	0.08514 to 0.7024	Yes	**	0.003
Notag FC vs. Notag DN	0.4627	0.1866 to 0.7387	Yes	****	<0.0001
Notag FC vs. NLS WT	0.2867	-0.02190 to 0.5953	No	ns	0.0926
Notag FC vs. NLS CA	0.1389	-0.1507 to 0.4284	No	ns	0.8995
Notag FC vs. NLS FC	0.3083	0.01874 to 0.5978	Yes	*	0.0273
Notag FC vs. NLS DN	0.5894	0.3134 to 0.8654	Yes	****	<0.0001
Notag FC vs. NES WT	0.1675	-0.1411 to 0.4762	No	ns	0.7966
Notag FC vs. NES CA	0.1465	-0.1621 to 0.4552	No	ns	0.9056
Notag FC vs. NES FC	0.187	-0.1216 to 0.4957	No	ns	0.661
Notag FC vs. NES DN	0.3993	0.09070 to 0.7079	Yes	**	0.0025
Notag DN vs. NLS WT	-0.1759	-0.4846 to 0.1327	No	ns	0.7412

Notag DN vs. NLS CA	-0.3238	-0.6133 to -0.03430	Yes	*	0.0161
Notag DN vs. NLS FC	-0.1544	-0.4439 to 0.1351	No	ns	0.8146
Notag DN vs. NLS DN	0.1267	-0.1493 to 0.4028	No	ns	0.924
Notag DN vs. NES WT	-0.2951	-0.6037 to 0.01350	No	ns	0.0736
Notag DN vs. NES CA	-0.3161	-0.6248 to -0.007507	Yes	*	0.04
Notag DN vs. NES FC	-0.2756	-0.5843 to 0.03299	No	ns	0.1239
Notag DN vs. NES DN	-0.06334	-0.3720 to 0.2453	No	ns	>0.9999
NLS WT vs. NLS CA	-0.1479	-0.4686 to 0.1729	No	ns	0.9219
NLS WT vs. NLS FC	0.02154	-0.2992 to 0.3423	No	ns	>0.9999
NLS WT vs. NLS DN	0.3027	-0.005939 to 0.6113	No	ns	0.0594
NLS WT vs. NES WT	-0.1192	-0.4573 to 0.2189	No	ns	0.9897
NLS WT vs. NES CA	-0.1402	-0.4783 to 0.1979	No	ns	0.9626
NLS WT vs. NES FC	-0.09969	-0.4378 to 0.2384	No	ns	0.9979
NLS WT vs. NES DN	0.1126	-0.2255 to 0.4507	No	ns	0.9937
NLS CA vs. NLS FC	0.1694	-0.1330 to 0.4718	No	ns	0.7619
NLS CA vs. NLS DN	0.4505	0.1610 to 0.7401	Yes	***	0.0001
NLS CA vs. NES WT	0.02868	-0.2920 to 0.3494	No	ns	>0.9999
NLS CA vs. NES CA	0.007679	-0.3131 to 0.3284	No	ns	>0.9999
NLS CA vs. NES FC	0.04818	-0.2726 to 0.3689	No	ns	>0.9999
NLS CA vs. NES DN	0.2605	-0.06026 to 0.5812	No	ns	0.225
NLS FC vs. NLS DN	0.2811	-0.008366 to 0.5707	No	ns	0.0647
NLS FC vs. NES WT	-0.1407	-0.4614 to 0.1800	No	ns	0.9441
NLS FC vs. NES CA	-0.1617	-0.4825 to 0.1590	No	ns	0.8642
NLS FC vs. NES FC	-0.1212	-0.4420 to 0.1995	No	ns	0.9817
NLS FC vs. NES DN	0.09107	-0.2297 to 0.4118	No	ns	0.9985
NLS DN vs. NES WT	-0.4219	-0.7305 to -0.1132	Yes	**	0.0011
NLS DN vs. NES CA	-0.4429	-0.7515 to -0.1342	Yes	***	0.0005
NLS DN vs. NES FC	-0.4024	-0.7110 to -0.09375	Yes	**	0.0022
NLS DN vs. NES DN	-0.1901	-0.4987 to 0.1185	No	ns	0.638
NES WT vs. NES CA	-0.021	-0.3591 to 0.3171	No	ns	>0.9999
NES WT vs. NES FC	0.01949	-0.3186 to 0.3576	No	ns	>0.9999
NES WT vs. NES DN	0.2318	-0.1063 to 0.5699	No	ns	0.4736
NES CA vs. NES FC	0.0405	-0.2976 to 0.3786	No	ns	>0.9999
NES CA vs. NES DN	0.2528	-0.08529 to 0.5909	No	ns	0.339
NES FC vs. NES DN	0.2123	-0.1258 to 0.5504	No	ns	0.6096

Figure 2D_Cellular Form Factor					
Tukey's multiple comparisons test	Mean Diff.	95.00% CI of diff.	Significant?	Summary	Adjusted P Value
GFP vs. Notag WT	0.0368	-0.1017 to 0.1753	No	ns	0.9992
GFP vs. Notag CA	-0.2755	-0.4140 to -0.1370	Yes	****	<0.0001
GFP vs. Notag FC	0.03275	-0.1057 to 0.1712	No	ns	0.9998
GFP vs. Notag DN	0.1598	0.02129 to 0.2983	Yes	*	0.0112
GFP vs. NLS WT	0.02199	-0.1314 to 0.1754	No	ns	>0.9999
GFP vs. NLS CA	-0.1676	-0.3122 to -0.02291	Yes	*	0.0107
GFP vs. NLS FC	0.0007892	-0.1439 to 0.1454	No	ns	>0.9999
GFP vs. NLS DN	0.0516	-0.08689 to 0.1901	No	ns	0.9838
GFP vs. NES WT	0.01425	-0.1392 to 0.1677	No	ns	>0.9999
GFP vs. NES CA	-0.27	-0.4234 to -0.1166	Yes	****	<0.0001
GFP vs. NES FC	-0.06069	-0.2141 to 0.09273	No	ns	0.9741
GFP vs. NES DN	0.1121	-0.03254 to 0.2568	No	ns	0.2878
Notag WT vs. Notag CA	-0.3123	-0.4444 to -0.1803	Yes	****	<0.0001
Notag WT vs. Notag FC	-0.004056	-0.1361 to 0.1280	No	ns	>0.9999
Notag WT vs. Notag DN	0.123	-0.009073 to 0.2550	No	ns	0.091
Notag WT vs. NLS WT	-0.01482	-0.1624 to 0.1328	No	ns	>0.9999
Notag WT vs. NLS CA	-0.2044	-0.3429 to -0.06587	Yes	***	0.0003
Notag WT vs. NLS FC	-0.03601	-0.1745 to 0.1025	No	ns	0.9994
Notag WT vs. NLS DN	0.0148	-0.1173 to 0.1468	No	ns	>0.9999
Notag WT vs. NES WT	-0.02255	-0.1702 to 0.1251	No	ns	>0.9999
Notag WT vs. NES CA	-0.3068	-0.4544 to -0.1592	Yes	****	<0.0001
Notag WT vs. NES FC	-0.09749	-0.2451 to 0.05014	No	ns	0.5338
Notag WT vs. NES DN	0.07531	-0.06319 to 0.2138	No	ns	0.7959
Notag CA vs. Notag FC	0.3083	0.1762 to 0.4403	Yes	****	<0.0001
Notag CA vs. Notag DN	0.4353	0.3032 to 0.5673	Yes	****	<0.0001
Notag CA vs. NLS WT	0.2975	0.1499 to 0.4451	Yes	****	<0.0001
Notag CA vs. NLS CA	0.108	-0.03053 to 0.2464	No	ns	0.2797
Notag CA vs. NLS FC	0.2763	0.1378 to 0.4148	Yes	****	<0.0001
Notag CA vs. NLS DN	0.3271	0.1951 to 0.4592	Yes	****	<0.0001
Notag CA vs. NES WT	0.2898	0.1421 to 0.4374	Yes	****	<0.0001
Notag CA vs. NES CA	0.005536	-0.1421 to 0.1532	No	ns	>0.9999
Notag CA vs. NES FC	0.2148	0.06720 to 0.3625	Yes	***	0.0004
Notag CA vs. NES DN	0.3876	0.2491 to 0.5261	Yes	****	<0.0001
Notag FC vs. Notag DN	0.127	-0.005017 to 0.2591	No	ns	0.0701
Notag FC vs. NLS WT	-0.01076	-0.1584 to 0.1369	No	ns	>0.9999
Notag FC vs. NLS CA	-0.2003	-0.3388 to -0.06182	Yes	***	0.0004
Notag FC vs. NLS FC	-0.03196	-0.1704 to 0.1065	No	ns	0.9998
Notag FC vs. NLS DN	0.01885	-0.1132 to 0.1509	No	ns	>0.9999
Notag FC vs. NES WT	-0.0185	-0.1661 to 0.1291	No	ns	>0.9999
Notag FC vs. NES CA	-0.3027	-0.4504 to -0.1551	Yes	****	<0.0001
Notag FC vs. NES FC	-0.09344	-0.2411 to 0.05419	No	ns	0.5991
Notag FC vs. NES DN	0.07936	-0.05913 to 0.2179	No	ns	0.7361
Notag DN vs. NLS WT	-0.1378	-0.2854 to 0.009843	No	ns	0.0894
Notag DN vs. NLS CA	-0.3273	-0.4658 to -0.1888	Yes	****	<0.0001

Notag DN vs. NLS FC	-0.159	-0.2975 to -0.02050	Yes	*	0.0119
Notag DN vs. NLS DN	-0.1082	-0.2402 to 0.02387	No	ns	0.2148
Notag DN vs. NES WT	-0.1455	-0.2932 to 0.002108	No	ns	0.0569
Notag DN vs. NES CA	-0.4298	-0.5774 to -0.2821	Yes	****	<0.0001
Notag DN vs. NES FC	-0.2205	-0.3681 to -0.07283	Yes	***	0.0002
Notag DN vs. NES DN	-0.04767	-0.1862 to 0.09082	No	ns	0.9917
NLS WT vs. NLS CA	-0.1895	-0.3430 to -0.03612	Yes	**	0.0047
NLS WT vs. NLS FC	-0.0212	-0.1746 to 0.1322	No	ns	>0.9999
NLS WT vs. NLS DN	0.02961	-0.1180 to 0.1772	No	ns	>0.9999
NLS WT vs. NES WT	-0.007735	-0.1695 to 0.1540	No	ns	>0.9999
NLS WT vs. NES CA	-0.292	-0.4537 to -0.1302	Yes	****	<0.0001
NLS WT vs. NES FC	-0.08268	-0.2444 to 0.07905	No	ns	0.8539
NLS WT vs. NES DN	0.09012	-0.06330 to 0.2435	No	ns	0.7046
NLS CA vs. NLS FC	0.1684	0.02370 to 0.3130	Yes	*	0.0101
NLS CA vs. NLS DN	0.2192	0.08067 to 0.3577	Yes	****	<0.0001
NLS CA vs. NES WT	0.1818	0.02839 to 0.3352	Yes	**	0.0081
NLS CA vs. NES CA	-0.1024	-0.2558 to 0.05100	No	ns	0.5168
NLS CA vs. NES FC	0.1069	-0.04655 to 0.2603	No	ns	0.4496
NLS CA vs. NES DN	0.2797	0.1350 to 0.4243	Yes	****	<0.0001
NLS FC vs. NLS DN	0.05081	-0.08768 to 0.1893	No	ns	0.9857
NLS FC vs. NES WT	0.01346	-0.1400 to 0.1669	No	ns	>0.9999
NLS FC vs. NES CA	-0.2708	-0.4242 to -0.1173	Yes	****	<0.0001
NLS FC vs. NES FC	-0.06148	-0.2149 to 0.09194	No	ns	0.9714
NLS FC vs. NES DN	0.1113	-0.03333 to 0.2560	No	ns	0.2978
NLS DN vs. NES WT	-0.03735	-0.1850 to 0.1103	No	ns	0.9995
NLS DN vs. NES CA	-0.3216	-0.4692 to -0.1739	Yes	****	<0.0001
NLS DN vs. NES FC	-0.1123	-0.2599 to 0.03534	No	ns	0.3147
NLS DN vs. NES DN	0.06051	-0.07798 to 0.1990	No	ns	0.9461
NES WT vs. NES CA	-0.2842	-0.4460 to -0.1225	Yes	****	<0.0001
NES WT vs. NES FC	-0.07494	-0.2367 to 0.08678	No	ns	0.9198
NES WT vs. NES DN	0.09786	-0.05557 to 0.2513	No	ns	0.5874
NES CA vs. NES FC	0.2093	0.04757 to 0.3710	Yes	**	0.0025
NES CA vs. NES DN	0.3821	0.2287 to 0.5355	Yes	****	<0.0001
NES FC vs. NES DN	0.1728	0.01937 to 0.3262	Yes	*	0.0148

Figure 2E_Cell Eccentricity					
**Tukey's multiple comparisons test**	**Mean Diff.**	**95.00% CI of diff.**	**Significant?**	**Summary**	**Adjusted P Value**
GFP vs. Notag WT	-0.02718	-0.08435 to 0.02998	No	ns	0.9047
GFP vs. Notag CA	-0.005036	-0.06220 to 0.05213	No	ns	>0.9999
GFP vs. Notag FC	-0.02596	-0.08313 to 0.03121	No	ns	0.9294
GFP vs. Notag DN	-0.009601	-0.06677 to 0.04757	No	ns	>0.9999
GFP vs. NLS WT	-0.03364	-0.09697 to 0.02969	No	ns	0.8186
GFP vs. NLS CA	-0.01255	-0.07226 to 0.04716	No	ns	>0.9999
GFP vs. NLS FC	-0.0166	-0.07631 to 0.04311	No	ns	0.9988
GFP vs. NLS DN	-0.02323	-0.08040 to 0.03394	No	ns	0.9679
GFP vs. NES WT	-0.02985	-0.09319 to 0.03348	No	ns	0.9099
GFP vs. NES CA	0.008742	-0.05459 to 0.07208	No	ns	>0.9999
GFP vs. NES FC	-0.03321	-0.09655 to 0.03012	No	ns	0.8306
GFP vs. NES DN	-0.0061	-0.06943 to 0.05723	No	ns	>0.9999
Notag WT vs. Notag CA	0.02215	-0.03236 to 0.07666	No	ns	0.9679
Notag WT vs. Notag FC	0.001227	-0.05328 to 0.05574	No	ns	>0.9999
Notag WT vs. Notag DN	0.01758	-0.03692 to 0.07209	No	ns	0.9952
Notag WT vs. NLS WT	-0.006456	-0.06740 to 0.05449	No	ns	>0.9999
Notag WT vs. NLS CA	0.01463	-0.04253 to 0.07180	No	ns	0.9995
Notag WT vs. NLS FC	0.01059	-0.04658 to 0.06776	No	ns	>0.9999
Notag WT vs. NLS DN	0.003954	-0.05055 to 0.05846	No	ns	>0.9999
Notag WT vs. NES WT	-0.002668	-0.06361 to 0.05827	No	ns	>0.9999
Notag WT vs. NES CA	0.03593	-0.02502 to 0.09687	No	ns	0.6986
Notag WT vs. NES FC	-0.00603	-0.06697 to 0.05491	No	ns	>0.9999
Notag WT vs. NES DN	0.02108	-0.03986 to 0.08203	No	ns	0.9912
Notag CA vs. Notag FC	-0.02092	-0.07543 to 0.03359	No	ns	0.9793
Notag CA vs. Notag DN	-0.004565	-0.05907 to 0.04994	No	ns	>0.9999
Notag CA vs. NLS WT	-0.0286	-0.08955 to 0.03234	No	ns	0.9123
Notag CA vs. NLS CA	-0.007514	-0.06468 to 0.04965	No	ns	>0.9999
Notag CA vs. NLS FC	-0.01156	-0.06873 to 0.04561	No	ns	>0.9999
Notag CA vs. NLS DN	-0.01819	-0.07270 to 0.03631	No	ns	0.9935
Notag CA vs. NES WT	-0.02482	-0.08576 to 0.03613	No	ns	0.9673
Notag CA vs. NES CA	0.01378	-0.04716 to 0.07472	No	ns	0.9998
Notag CA vs. NES FC	-0.02818	-0.08912 to 0.03276	No	ns	0.9204
Notag CA vs. NES DN	-0.001064	-0.06201 to 0.05988	No	ns	>0.9999
Notag FC vs. Notag DN	0.01636	-0.03815 to 0.07087	No	ns	0.9975
Notag FC vs. NLS WT	-0.007683	-0.06862 to 0.05326	No	ns	>0.9999
Notag FC vs. NLS CA	0.01341	-0.04376 to 0.07058	No	ns	0.9998
Notag FC vs. NLS FC	0.009361	-0.04781 to 0.06653	No	ns	>0.9999
Notag FC vs. NLS DN	0.002728	-0.05178 to 0.05724	No	ns	>0.9999
Notag FC vs. NES WT	-0.003895	-0.06484 to 0.05705	No	ns	>0.9999
Notag FC vs. NES CA	0.0347	-0.02624 to 0.09564	No	ns	0.7427
Notag FC vs. NES FC	-0.007257	-0.06820 to 0.05369	No	ns	>0.9999
Notag FC vs. NES DN	0.01986	-0.04108 to 0.08080	No	ns	0.9948
Notag DN vs. NLS WT	-0.02404	-0.08498 to 0.03690	No	ns	0.9744
Notag DN vs. NLS CA	-0.002949	-0.06012 to 0.05422	No	ns	>0.9999

Notag DN vs. NLS FC	-0.006996	-0.06417 to 0.05017	No	ns	>0.9999
Notag DN vs. NLS DN	-0.01363	-0.06814 to 0.04088	No	ns	0.9996
Notag DN vs. NES WT	-0.02025	-0.08119 to 0.04069	No	ns	0.9938
Notag DN vs. NES CA	0.01834	-0.04260 to 0.07929	No	ns	0.9975
Notag DN vs. NES FC	-0.02361	-0.08456 to 0.03733	No	ns	0.9777
Notag DN vs. NES DN	0.003501	-0.05744 to 0.06444	No	ns	>0.9999
NLS WT vs. NLS CA	0.02109	-0.04224 to 0.08442	No	ns	0.9937
NLS WT vs. NLS FC	0.01704	-0.04629 to 0.08038	No	ns	0.9991
NLS WT vs. NLS DN	0.01041	-0.05053 to 0.07135	No	ns	>0.9999
NLS WT vs. NES WT	0.003787	-0.06297 to 0.07055	No	ns	>0.9999
NLS WT vs. NES CA	0.04238	-0.02438 to 0.1091	No	ns	0.5932
NLS WT vs. NES FC	0.0004258	-0.06633 to 0.06718	No	ns	>0.9999
NLS WT vs. NES DN	0.02754	-0.03922 to 0.09430	No	ns	0.964
NLS CA vs. NLS FC	-0.004047	-0.06376 to 0.05566	No	ns	>0.9999
NLS CA vs. NLS DN	-0.01068	-0.06785 to 0.04649	No	ns	>0.9999
NLS CA vs. NES WT	-0.0173	-0.08064 to 0.04603	No	ns	0.999
NLS CA vs. NES CA	0.02129	-0.04204 to 0.08463	No	ns	0.9931
NLS CA vs. NES FC	-0.02066	-0.08400 to 0.04267	No	ns	0.9947
NLS CA vs. NES DN	0.00645	-0.05688 to 0.06978	No	ns	>0.9999
NLS FC vs. NLS DN	-0.006633	-0.06380 to 0.05054	No	ns	>0.9999
NLS FC vs. NES WT	-0.01326	-0.07659 to 0.05008	No	ns	>0.9999
NLS FC vs. NES CA	0.02534	-0.03799 to 0.08867	No	ns	0.9714
NLS FC vs. NES FC	-0.01662	-0.07995 to 0.04672	No	ns	0.9993
NLS FC vs. NES DN	0.0105	-0.05284 to 0.07383	No	ns	>0.9999
NLS DN vs. NES WT	-0.006623	-0.06757 to 0.05432	No	ns	>0.9999
NLS DN vs. NES CA	0.03197	-0.02897 to 0.09291	No	ns	0.8303
NLS DN vs. NES FC	-0.009984	-0.07093 to 0.05096	No	ns	>0.9999
NLS DN vs. NES DN	0.01713	-0.04381 to 0.07807	No	ns	0.9987
NES WT vs. NES CA	0.0386	-0.02816 to 0.1054	No	ns	0.7239
NES WT vs. NES FC	-0.003362	-0.07012 to 0.06340	No	ns	>0.9999
NES WT vs. NES DN	0.02375	-0.04301 to 0.09051	No	ns	0.9888
NES CA vs. NES FC	-0.04196	-0.1087 to 0.02480	No	ns	0.6083
NES CA vs. NES DN	-0.01484	-0.08160 to 0.05192	No	ns	0.9999
NES FC vs. NES DN	0.02711	-0.03964 to 0.09387	No	ns	0.968

**Figure 3A_Nuclear perimeter**					
**Tukey's multiple comparisons test**	**Mean Diff.**	**95.00% CI of diff.**	**Significant?**	**Summary**	**Adjusted P Value**
GFP vs. Notag WT	-0.1021	-0.1719 to -0.03225	Yes	***	0.0004
GFP vs. Notag CA	-0.01176	-0.08160 to 0.05808	No	ns	>0.9999
GFP vs. Notag FC	-0.153	-0.2229 to -0.08321	Yes	****	<0.0001
GFP vs. Notag DN	-0.05403	-0.1239 to 0.01581	No	ns	0.2896
GFP vs. NLS WT	-0.1089	-0.1863 to -0.03151	Yes	***	0.0007
GFP vs. NLS CA	-0.1342	-0.2072 to -0.06127	Yes	****	<0.0001
GFP vs. NLS FC	-0.123	-0.1960 to -0.05010	Yes	****	<0.0001
GFP vs. NLS DN	-0.007699	-0.07754 to 0.06214	No	ns	>0.9999
GFP vs. NES WT	-0.1105	-0.1879 to -0.03313	Yes	***	0.0005
GFP vs. NES CA	0.002116	-0.07526 to 0.07949	No	ns	>0.9999
GFP vs. NES FC	-0.08943	-0.1668 to -0.01205	Yes	*	0.011
GFP vs. NES DN	-0.01422	-0.09159 to 0.06315	No	ns	>0.9999
Notag WT vs. Notag CA	0.09033	0.02374 to 0.1569	Yes	**	0.0012
Notag WT vs. Notag FC	-0.05095	-0.1175 to 0.01564	No	ns	0.3053
Notag WT vs. Notag DN	0.04806	-0.01853 to 0.1147	No	ns	0.3929
Notag WT vs. NLS WT	-0.006784	-0.08124 to 0.06767	No	ns	>0.9999
Notag WT vs. NLS CA	-0.03212	-0.1020 to 0.03772	No	ns	0.9231
Notag WT vs. NLS FC	-0.02095	-0.09080 to 0.04889	No	ns	0.9975
Notag WT vs. NLS DN	0.0944	0.02780 to 0.1610	Yes	***	0.0006
Notag WT vs. NES WT	-0.008404	-0.08286 to 0.06605	No	ns	>0.9999
Notag WT vs. NES CA	0.1042	0.02976 to 0.1787	Yes	***	0.0007
Notag WT vs. NES FC	0.01267	-0.06178 to 0.08712	No	ns	>0.9999
Notag WT vs. NES DN	0.08787	0.01342 to 0.1623	Yes	**	0.0085
Notag CA vs. Notag FC	-0.1413	-0.2079 to -0.07469	Yes	****	<0.0001
Notag CA vs. Notag DN	-0.04227	-0.1089 to 0.02432	No	ns	0.5934
Notag CA vs. NLS WT	-0.09712	-0.1716 to -0.02267	Yes	**	0.0022
Notag CA vs. NLS CA	-0.1225	-0.1923 to -0.05261	Yes	****	<0.0001
Notag CA vs. NLS FC	-0.1113	-0.1811 to -0.04144	Yes	****	<0.0001
Notag CA vs. NLS DN	0.004062	-0.06253 to 0.07065	No	ns	>0.9999
Notag CA vs. NES WT	-0.09874	-0.1732 to -0.02429	Yes	**	0.0017
Notag CA vs. NES CA	0.01388	-0.06058 to 0.08833	No	ns	>0.9999
Notag CA vs. NES FC	-0.07766	-0.1521 to -0.003211	Yes	*	0.0336
Notag CA vs. NES DN	-0.002459	-0.07691 to 0.07199	No	ns	>0.9999
Notag FC vs. Notag DN	0.09902	0.03242 to 0.1656	Yes	***	0.0003
Notag FC vs. NLS WT	0.04417	-0.03028 to 0.1186	No	ns	0.6902
Notag FC vs. NLS CA	0.01883	-0.05101 to 0.08867	No	ns	0.9991
Notag FC vs. NLS FC	0.03	-0.03984 to 0.09984	No	ns	0.9518
Notag FC vs. NLS DN	0.1453	0.07876 to 0.2119	Yes	****	<0.0001
Notag FC vs. NES WT	0.04255	-0.03190 to 0.1170	No	ns	0.7382
Notag FC vs. NES CA	0.1552	0.08071 to 0.2296	Yes	****	<0.0001
Notag FC vs. NES FC	0.06362	-0.01083 to 0.1381	No	ns	0.1663
Notag FC vs. NES DN	0.1388	0.06438 to 0.2133	Yes	****	<0.0001
Notag DN vs. NLS WT	-0.05485	-0.1293 to 0.01961	No	ns	0.3614
Notag DN vs. NLS CA	-0.08018	-0.1500 to -0.01034	Yes	*	0.0119

Notag DN vs. NLS FC	-0.06902	-0.1389 to 0.0008272	No	ns	0.0556
Notag DN vs. NLS DN	0.04633	-0.02026 to 0.1129	No	ns	0.4503
Notag DN vs. NES WT	-0.05647	-0.1309 to 0.01799	No	ns	0.3181
Notag DN vs. NES CA	0.05615	-0.01830 to 0.1306	No	ns	0.3263
Notag DN vs. NES FC	-0.03539	-0.1098 to 0.03906	No	ns	0.9049
Notag DN vs. NES DN	0.03981	-0.03464 to 0.1143	No	ns	0.812
NLS WT vs. NLS CA	-0.02534	-0.1027 to 0.05203	No	ns	0.9945
NLS WT vs. NLS FC	-0.01417	-0.09154 to 0.06320	No	ns	>0.9999
NLS WT vs. NLS DN	0.1012	0.02673 to 0.1756	Yes	**	0.0012
NLS WT vs. NES WT	-0.00162	-0.08318 to 0.07994	No	ns	>0.9999
NLS WT vs. NES CA	0.111	0.02944 to 0.1926	Yes	**	0.0012
NLS WT vs. NES FC	0.01945	-0.06210 to 0.1010	No	ns	0.9997
NLS WT vs. NES DN	0.09466	0.01310 to 0.1762	Yes	*	0.0105
NLS CA vs. NLS FC	0.01117	-0.06178 to 0.08412	No	ns	>0.9999
NLS CA vs. NLS DN	0.1265	0.05668 to 0.1964	Yes	****	<0.0001
NLS CA vs. NES WT	0.02372	-0.05366 to 0.1011	No	ns	0.997
NLS CA vs. NES CA	0.1363	0.05896 to 0.2137	Yes	****	<0.0001
NLS CA vs. NES FC	0.04479	-0.03258 to 0.1222	No	ns	0.7221
NLS CA vs. NES DN	0.12	0.04262 to 0.1974	Yes	***	0.0001
NLS FC vs. NLS DN	0.1153	0.04551 to 0.1852	Yes	****	<0.0001
NLS FC vs. NES WT	0.01255	-0.06482 to 0.08992	No	ns	>0.9999
NLS FC vs. NES CA	0.1252	0.04779 to 0.2025	Yes	****	<0.0001
NLS FC vs. NES FC	0.03362	-0.04375 to 0.1110	No	ns	0.9477
NLS FC vs. NES DN	0.1088	0.03145 to 0.1862	Yes	***	0.0007
NLS DN vs. NES WT	-0.1028	-0.1773 to -0.02835	Yes	***	0.0009
NLS DN vs. NES CA	0.009816	-0.06464 to 0.08427	No	ns	>0.9999
NLS DN vs. NES FC	-0.08173	-0.1562 to -0.007273	Yes	*	0.0198
NLS DN vs. NES DN	-0.006521	-0.08097 to 0.06793	No	ns	>0.9999
NES WT vs. NES CA	0.1126	0.03106 to 0.1942	Yes	***	0.0009
NES WT vs. NES FC	0.02107	-0.06048 to 0.1026	No	ns	0.9994
NES WT vs. NES DN	0.09628	0.01472 to 0.1778	Yes	**	0.0085
NES CA vs. NES FC	-0.09154	-0.1731 to -0.009982	Yes	*	0.0155
NES CA vs. NES DN	-0.01634	-0.09790 to 0.06522	No	ns	>0.9999
NES FC vs. NES DN	0.0752	-0.006355 to 0.1568	No	ns	0.0981

Figure 3B_Nuclear Area					
**Tukey's multiple comparisons test**	**Mean Diff.**	**95.00% CI of diff.**	**Significant?**	**Summary**	**Adjusted P Value**
GFP vs. Notag WT	-0.2076	-0.3368 to -0.07843	Yes	****	<0.0001
GFP vs. Notag CA	-0.01713	-0.1463 to 0.1121	No	ns	>0.9999
GFP vs. Notag FC	-0.3153	-0.4445 to -0.1861	Yes	****	<0.0001
GFP vs. Notag DN	-0.05964	-0.1888 to 0.06957	No	ns	0.9213
GFP vs. NLS WT	-0.2265	-0.3696 to -0.08335	Yes	****	<0.0001
GFP vs. NLS CA	-0.2534	-0.3883 to -0.1184	Yes	****	<0.0001
GFP vs. NLS FC	-0.24	-0.3749 to -0.1050	Yes	****	<0.0001
GFP vs. NLS DN	-0.01026	-0.1395 to 0.1189	No	ns	>0.9999
GFP vs. NES WT	-0.2139	-0.3571 to -0.07081	Yes	***	0.0002
GFP vs. NES CA	-0.008219	-0.1514 to 0.1349	No	ns	>0.9999
GFP vs. NES FC	-0.1645	-0.3076 to -0.02134	Yes	*	0.0118
GFP vs. NES DN	0.001915	-0.1412 to 0.1450	No	ns	>0.9999
Notag WT vs. Notag CA	0.1905	0.06732 to 0.3137	Yes	***	0.0001
Notag WT vs. Notag FC	-0.1076	-0.2308 to 0.01554	No	ns	0.1439
Notag WT vs. Notag DN	0.148	0.02481 to 0.2712	Yes	**	0.0068
Notag WT vs. NLS WT	-0.01885	-0.1566 to 0.1189	No	ns	>0.9999
Notag WT vs. NLS CA	-0.04573	-0.1749 to 0.08348	No	ns	0.9893
Notag WT vs. NLS FC	-0.03235	-0.1615 to 0.09686	No	ns	0.9996
Notag WT vs. NLS DN	0.1974	0.07418 to 0.3206	Yes	****	<0.0001
Notag WT vs. NES WT	-0.006303	-0.1440 to 0.1314	No	ns	>0.9999
Notag WT vs. NES CA	0.1994	0.06169 to 0.3371	Yes	***	0.0004
Notag WT vs. NES FC	0.04316	-0.09457 to 0.1809	No	ns	0.9963
Notag WT vs. NES DN	0.2096	0.07182 to 0.3473	Yes	***	0.0002
Notag CA vs. Notag FC	-0.2982	-0.4213 to -0.1750	Yes	****	<0.0001
Notag CA vs. Notag DN	-0.04251	-0.1657 to 0.08068	No	ns	0.9914
Notag CA vs. NLS WT	-0.2094	-0.3471 to -0.07163	Yes	***	0.0002
Notag CA vs. NLS CA	-0.2362	-0.3654 to -0.1070	Yes	****	<0.0001
Notag CA vs. NLS FC	-0.2229	-0.3521 to -0.09365	Yes	****	<0.0001
Notag CA vs. NLS DN	0.006864	-0.1163 to 0.1301	No	ns	>0.9999
Notag CA vs. NES WT	-0.1968	-0.3345 to -0.05908	Yes	***	0.0005
Notag CA vs. NES CA	0.008908	-0.1288 to 0.1466	No	ns	>0.9999
Notag CA vs. NES FC	-0.1474	-0.2851 to -0.009621	Yes	*	0.026
Notag CA vs. NES DN	0.01904	-0.1187 to 0.1568	No	ns	>0.9999
Notag FC vs. Notag DN	0.2556	0.1325 to 0.3788	Yes	****	<0.0001
Notag FC vs. NLS WT	0.08879	-0.04894 to 0.2265	No	ns	0.5698
Notag FC vs. NLS CA	0.06192	-0.06728 to 0.1911	No	ns	0.9
Notag FC vs. NLS FC	0.0753	-0.05390 to 0.2045	No	ns	0.7136
Notag FC vs. NLS DN	0.305	0.1818 to 0.4282	Yes	****	<0.0001
Notag FC vs. NES WT	0.1013	-0.03639 to 0.2391	No	ns	0.3632
Notag FC vs. NES CA	0.3071	0.1693 to 0.4448	Yes	****	<0.0001
Notag FC vs. NES FC	0.1508	0.01307 to 0.2885	Yes	*	0.0203
Notag FC vs. NES DN	0.3172	0.1795 to 0.4549	Yes	****	<0.0001
Notag DN vs. NLS WT	-0.1669	-0.3046 to -0.02912	Yes	**	0.0061
Notag DN vs. NLS CA	-0.1937	-0.3229 to -0.06452	Yes	***	0.0002

Notag DN vs. NLS FC	-0.1803	-0.3095 to -0.05114	Yes	***	0.0008
Notag DN vs. NLS DN	0.04937	-0.07382 to 0.1726	No	ns	0.971
Notag DN vs. NES WT	-0.1543	-0.2920 to -0.01657	Yes	*	0.0158
Notag DN vs. NES CA	0.05142	-0.08631 to 0.1891	No	ns	0.9834
Notag DN vs. NES FC	-0.1048	-0.2426 to 0.03289	No	ns	0.3127
Notag DN vs. NES DN	0.06155	-0.07618 to 0.1993	No	ns	0.9365
NLS WT vs. NLS CA	-0.02687	-0.1700 to 0.1163	No	ns	>0.9999
NLS WT vs. NLS FC	-0.01349	-0.1566 to 0.1296	No	ns	>0.9999
NLS WT vs. NLS DN	0.2162	0.07850 to 0.3540	Yes	****	<0.0001
NLS WT vs. NES WT	0.01255	-0.1383 to 0.1634	No	ns	>0.9999
NLS WT vs. NES CA	0.2183	0.06739 to 0.3691	Yes	***	0.0004
NLS WT vs. NES FC	0.06201	-0.08887 to 0.2129	No	ns	0.965
NLS WT vs. NES DN	0.2284	0.07753 to 0.3793	Yes	***	0.0002
NLS CA vs. NLS FC	0.01338	-0.1216 to 0.1483	No	ns	>0.9999
NLS CA vs. NLS DN	0.2431	0.1139 to 0.3723	Yes	****	<0.0001
NLS CA vs. NES WT	0.03942	-0.1037 to 0.1826	No	ns	0.9989
NLS CA vs. NES CA	0.2451	0.1020 to 0.3883	Yes	****	<0.0001
NLS CA vs. NES FC	0.08888	-0.05425 to 0.2320	No	ns	0.626
NLS CA vs. NES DN	0.2553	0.1121 to 0.3984	Yes	****	<0.0001
NLS FC vs. NLS DN	0.2297	0.1005 to 0.3589	Yes	****	<0.0001
NLS FC vs. NES WT	0.02604	-0.1171 to 0.1692	No	ns	>0.9999
NLS FC vs. NES CA	0.2318	0.08863 to 0.3749	Yes	****	<0.0001
NLS FC vs. NES FC	0.0755	-0.06763 to 0.2186	No	ns	0.8252
NLS FC vs. NES DN	0.2419	0.09876 to 0.3850	Yes	****	<0.0001
NLS DN vs. NES WT	-0.2037	-0.3414 to -0.06595	Yes	***	0.0003
NLS DN vs. NES CA	0.002043	-0.1357 to 0.1398	No	ns	>0.9999
NLS DN vs. NES FC	-0.1542	-0.2919 to -0.01648	Yes	*	0.0159
NLS DN vs. NES DN	0.01218	-0.1256 to 0.1499	No	ns	>0.9999
NES WT vs. NES CA	0.2057	0.05484 to 0.3566	Yes	**	0.0011
NES WT vs. NES FC	0.04946	-0.1014 to 0.2003	No	ns	0.9945
NES WT vs. NES DN	0.2159	0.06498 to 0.3667	Yes	***	0.0005
NES CA vs. NES FC	-0.1563	-0.3071 to -0.005383	Yes	*	0.036
NES CA vs. NES DN	0.01013	-0.1407 to 0.1610	No	ns	>0.9999
NES FC vs. NES DN	0.1664	0.01552 to 0.3173	Yes	*	0.0188

Figure 3C_Nuclear Eccentricity					
**Tukey's multiple comparisons test**	**Mean Diff.**	**95.00% CI of diff.**	**Significant?**	**Summary**	**Adjusted P Value**
GFP vs. Notag WT	-0.04368	-0.1351 to 0.04774	No	ns	0.9018
GFP vs. Notag CA	-0.1093	-0.2007 to -0.01785	Yes	**	0.0073
GFP vs. Notag FC	-0.04858	-0.1400 to 0.04284	No	ns	0.8182
GFP vs. Notag DN	0.01008	-0.08134 to 0.1015	No	ns	>0.9999
GFP vs. NLS WT	-0.05071	-0.1520 to 0.05056	No	ns	0.8696
GFP vs. NLS CA	-0.04841	-0.1439 to 0.04708	No	ns	0.8599
GFP vs. NLS FC	-0.06831	-0.1638 to 0.02717	No	ns	0.4065
GFP vs. NLS DN	0.01554	-0.07588 to 0.1070	No	ns	>0.9999
GFP vs. NES WT	-0.06378	-0.1651 to 0.03750	No	ns	0.6053
GFP vs. NES CA	-0.03539	-0.1367 to 0.06588	No	ns	0.9904
GFP vs. NES FC	-0.08388	-0.1852 to 0.01740	No	ns	0.2012
GFP vs. NES DN	-0.04656	-0.1478 to 0.05472	No	ns	0.9233
Notag WT vs. Notag CA	-0.06558	-0.1527 to 0.02158	No	ns	0.3297
Notag WT vs. Notag FC	-0.004897	-0.09206 to 0.08227	No	ns	>0.9999
Notag WT vs. Notag DN	0.05376	-0.03340 to 0.1409	No	ns	0.6359
Notag WT vs. NLS WT	-0.007031	-0.1045 to 0.09042	No	ns	>0.9999
Notag WT vs. NLS CA	-0.004725	-0.09614 to 0.08669	No	ns	>0.9999
Notag WT vs. NLS FC	-0.02463	-0.1160 to 0.06679	No	ns	0.9991
Notag WT vs. NLS DN	0.05922	-0.02795 to 0.1464	No	ns	0.488
Notag WT vs. NES WT	-0.0201	-0.1176 to 0.07736	No	ns	>0.9999
Notag WT vs. NES CA	0.008288	-0.08917 to 0.1057	No	ns	>0.9999
Notag WT vs. NES FC	-0.0402	-0.1377 to 0.05726	No	ns	0.9641
Notag WT vs. NES DN	-0.002877	-0.1003 to 0.09458	No	ns	>0.9999
Notag CA vs. Notag FC	0.06069	-0.02648 to 0.1479	No	ns	0.4493
Notag CA vs. Notag DN	0.1193	0.03218 to 0.2065	Yes	**	0.0011
Notag CA vs. NLS WT	0.05855	-0.03890 to 0.1560	No	ns	0.673
Notag CA vs. NLS CA	0.06086	-0.03056 to 0.1523	No	ns	0.52
Notag CA vs. NLS FC	0.04095	-0.05047 to 0.1324	No	ns	0.9355
Notag CA vs. NLS DN	0.1248	0.03764 to 0.2120	Yes	***	0.0005
Notag CA vs. NES WT	0.04549	-0.05197 to 0.1429	No	ns	0.9154
Notag CA vs. NES CA	0.07387	-0.02358 to 0.1713	No	ns	0.3188
Notag CA vs. NES FC	0.02539	-0.07207 to 0.1228	No	ns	0.9994
Notag CA vs. NES DN	0.06271	-0.03475 to 0.1602	No	ns	0.5728
Notag FC vs. Notag DN	0.05866	-0.02851 to 0.1458	No	ns	0.5029
Notag FC vs. NLS WT	-0.002134	-0.09959 to 0.09532	No	ns	>0.9999
Notag FC vs. NLS CA	0.0001717	-0.09125 to 0.09159	No	ns	>0.9999
Notag FC vs. NLS FC	-0.01973	-0.1112 to 0.07169	No	ns	>0.9999
Notag FC vs. NLS DN	0.06412	-0.02305 to 0.1513	No	ns	0.3637
Notag FC vs. NES WT	-0.0152	-0.1127 to 0.08225	No	ns	>0.9999
Notag FC vs. NES CA	0.01319	-0.08427 to 0.1106	No	ns	>0.9999
Notag FC vs. NES FC	-0.0353	-0.1328 to 0.06215	No	ns	0.987
Notag FC vs. NES DN	0.00202	-0.09543 to 0.09947	No	ns	>0.9999
Notag DN vs. NLS WT	-0.06079	-0.1582 to 0.03666	No	ns	0.6193
Notag DN vs. NLS CA	-0.05849	-0.1499 to 0.03293	No	ns	0.5815

Notag DN vs. NLS FC	-0.07839	-0.1698 to 0.01303	No	ns	0.1627
Notag DN vs. NLS DN	0.005456	-0.08171 to 0.09262	No	ns	>0.9999
Notag DN vs. NES WT	-0.07386	-0.1713 to 0.02359	No	ns	0.319
Notag DN vs. NES CA	-0.04547	-0.1429 to 0.05198	No	ns	0.9155
Notag DN vs. NES FC	-0.09396	-0.1914 to 0.003494	No	ns	0.0687
Notag DN vs. NES DN	-0.05664	-0.1541 to 0.04081	No	ns	0.7171
NLS WT vs. NLS CA	0.002306	-0.09897 to 0.1036	No	ns	>0.9999
NLS WT vs. NLS FC	-0.0176	-0.1189 to 0.08368	No	ns	>0.9999
NLS WT vs. NLS DN	0.06625	-0.03120 to 0.1637	No	ns	0.487
NLS WT vs. NES WT	-0.01307	-0.1198 to 0.09369	No	ns	>0.9999
NLS WT vs. NES CA	0.01532	-0.09144 to 0.1221	No	ns	>0.9999
NLS WT vs. NES FC	-0.03317	-0.1399 to 0.07359	No	ns	0.9966
NLS WT vs. NES DN	0.004154	-0.1026 to 0.1109	No	ns	>0.9999
NLS CA vs. NLS FC	-0.0199	-0.1154 to 0.07558	No	ns	>0.9999
NLS CA vs. NLS DN	0.06394	-0.02748 to 0.1554	No	ns	0.4419
NLS CA vs. NES WT	-0.01537	-0.1166 to 0.08590	No	ns	>0.9999
NLS CA vs. NES CA	0.01301	-0.08826 to 0.1143	No	ns	>0.9999
NLS CA vs. NES FC	-0.03547	-0.1367 to 0.06581	No	ns	0.9902
NLS CA vs. NES DN	0.001848	-0.09943 to 0.1031	No	ns	>0.9999
NLS FC vs. NLS DN	0.08385	-0.007571 to 0.1753	No	ns	0.1021
NLS FC vs. NES WT	0.004532	-0.09675 to 0.1058	No	ns	>0.9999
NLS FC vs. NES CA	0.03292	-0.06836 to 0.1342	No	ns	0.9949
NLS FC vs. NES FC	-0.01557	-0.1168 to 0.08571	No	ns	>0.9999
NLS FC vs. NES DN	0.02175	-0.07952 to 0.1230	No	ns	>0.9999
NLS DN vs. NES WT	-0.07932	-0.1768 to 0.01814	No	ns	0.2223
NLS DN vs. NES CA	-0.05093	-0.1484 to 0.04652	No	ns	0.8338
NLS DN vs. NES FC	-0.09942	-0.1969 to -0.001962	Yes	*	0.0416
NLS DN vs. NES DN	-0.0621	-0.1596 to 0.03536	No	ns	0.5877
NES WT vs. NES CA	0.02839	-0.07837 to 0.1351	No	ns	0.9992
NES WT vs. NES FC	-0.0201	-0.1269 to 0.08666	No	ns	>0.9999
NES WT vs. NES DN	0.01722	-0.08954 to 0.1240	No	ns	>0.9999
NES CA vs. NES FC	-0.04849	-0.1552 to 0.05827	No	ns	0.9292
NES CA vs. NES DN	-0.01117	-0.1179 to 0.09559	No	ns	>0.9999
NES FC vs. NES DN	0.03732	-0.06944 to 0.1441	No	ns	0.9904

Figure 3D_Nuclear Form Factor					
**Tukey's multiple comparisons test**	**Mean Diff.**	**95.00% CI of diff.**	**Significant?**	**Summary**	**Adjusted P Value**
GFP vs. Notag WT	0.01237	-0.01469 to 0.03942	No	ns	0.9266
GFP vs. Notag CA	0.01821	-0.008848 to 0.04526	No	ns	0.5042
GFP vs. Notag FC	0.02032	-0.006738 to 0.04737	No	ns	0.3334
GFP vs. Notag DN	0.03218	0.005120 to 0.05923	Yes	**	0.0077
GFP vs. NLS WT	0.005961	-0.02401 to 0.03593	No	ns	>0.9999
GFP vs. NLS CA	0.02303	-0.005232 to 0.05128	No	ns	0.2214
GFP vs. NLS FC	0.02096	-0.007300 to 0.04922	No	ns	0.3521
GFP vs. NLS DN	0.006784	-0.02027 to 0.03384	No	ns	0.9996
GFP vs. NES WT	0.0167	-0.01327 to 0.04667	No	ns	0.7693
GFP vs. NES CA	-0.003737	-0.03371 to 0.02623	No	ns	>0.9999
GFP vs. NES FC	0.01954	-0.01043 to 0.04952	No	ns	0.5535
GFP vs. NES DN	0.01831	-0.009952 to 0.04656	No	ns	0.5637
Notag WT vs. Notag CA	0.005837	-0.01996 to 0.03163	No	ns	0.9998
Notag WT vs. Notag FC	0.007948	-0.01785 to 0.03374	No	ns	0.9969
Notag WT vs. Notag DN	0.01981	-0.005990 to 0.04560	No	ns	0.3011
Notag WT vs. NLS WT	-0.006409	-0.03525 to 0.02243	No	ns	0.9999
Notag WT vs. NLS CA	0.01066	-0.01640 to 0.03771	No	ns	0.9749
Notag WT vs. NLS FC	0.008588	-0.01847 to 0.03564	No	ns	0.9959
Notag WT vs. NLS DN	-0.005585	-0.03138 to 0.02021	No	ns	>0.9999
Notag WT vs. NES WT	0.00433	-0.02451 to 0.03317	No	ns	>0.9999
Notag WT vs. NES CA	-0.01611	-0.04495 to 0.01273	No	ns	0.7666
Notag WT vs. NES FC	0.007174	-0.02167 to 0.03601	No	ns	0.9996
Notag WT vs. NES DN	0.005936	-0.02112 to 0.03299	No	ns	0.9999
Notag CA vs. Notag FC	0.002111	-0.02369 to 0.02791	No	ns	>0.9999
Notag CA vs. Notag DN	0.01397	-0.01183 to 0.03976	No	ns	0.8002
Notag CA vs. NLS WT	-0.01225	-0.04109 to 0.01659	No	ns	0.956
Notag CA vs. NLS CA	0.004819	-0.02224 to 0.03187	No	ns	>0.9999
Notag CA vs. NLS FC	0.002751	-0.02430 to 0.02981	No	ns	>0.9999
Notag CA vs. NLS DN	-0.01142	-0.03722 to 0.01437	No	ns	0.9409
Notag CA vs. NES WT	-0.001507	-0.03035 to 0.02733	No	ns	>0.9999
Notag CA vs. NES CA	-0.02194	-0.05078 to 0.006897	No	ns	0.3142
Notag CA vs. NES FC	0.001337	-0.02750 to 0.03018	No	ns	>0.9999
Notag CA vs. NES DN	0.00009935	-0.02696 to 0.02715	No	ns	>0.9999
Notag FC vs. Notag DN	0.01186	-0.01394 to 0.03765	No	ns	0.9239
Notag FC vs. NLS WT	-0.01436	-0.04320 to 0.01448	No	ns	0.8749
Notag FC vs. NLS CA	0.002709	-0.02435 to 0.02976	No	ns	>0.9999
Notag FC vs. NLS FC	0.0006404	-0.02641 to 0.02770	No	ns	>0.9999
Notag FC vs. NLS DN	-0.01353	-0.03933 to 0.01226	No	ns	0.8313
Notag FC vs. NES WT	-0.003617	-0.03246 to 0.02522	No	ns	>0.9999
Notag FC vs. NES CA	-0.02405	-0.05290 to 0.004786	No	ns	0.1935
Notag FC vs. NES FC	-0.0007735	-0.02961 to 0.02807	No	ns	>0.9999
Notag FC vs. NES DN	-0.002011	-0.02907 to 0.02504	No	ns	>0.9999
Notag DN vs. NLS WT	-0.02621	-0.05506 to 0.002626	No	ns	0.1093
Notag DN vs. NLS CA	-0.009149	-0.03620 to 0.01791	No	ns	0.9928

Notag DN vs. NLS FC	-0.01122	-0.03827 to 0.01584	No	ns	0.963
Notag DN vs. NLS DN	-0.02539	-0.05119 to 0.0004048	No	ns	0.0576
Notag DN vs. NES WT	-0.01548	-0.04432 to 0.01337	No	ns	0.8096
Notag DN vs. NES CA	-0.03591	-0.06475 to -0.007072	Yes	**	0.0042
Notag DN vs. NES FC	-0.01263	-0.04147 to 0.01621	No	ns	0.9452
Notag DN vs. NES DN	-0.01387	-0.04092 to 0.01319	No	ns	0.8516
NLS WT vs. NLS CA	0.01707	-0.01291 to 0.04704	No	ns	0.7439
NLS WT vs. NLS FC	0.015	-0.01498 to 0.04497	No	ns	0.871
NLS WT vs. NLS DN	0.0008234	-0.02802 to 0.02966	No	ns	>0.9999
NLS WT vs. NES WT	0.01074	-0.02085 to 0.04233	No	ns	0.9925
NLS WT vs. NES CA	-0.009698	-0.04129 to 0.02190	No	ns	0.997
NLS WT vs. NES FC	0.01358	-0.01801 to 0.04518	No	ns	0.9519
NLS WT vs. NES DN	0.01235	-0.01763 to 0.04232	No	ns	0.9648
NLS CA vs. NLS FC	-0.002068	-0.03033 to 0.02619	No	ns	>0.9999
NLS CA vs. NLS DN	-0.01624	-0.04330 to 0.01081	No	ns	0.6753
NLS CA vs. NES WT	-0.006326	-0.03630 to 0.02365	No	ns	>0.9999
NLS CA vs. NES CA	-0.02676	-0.05674 to 0.003209	No	ns	0.1242
NLS CA vs. NES FC	-0.003482	-0.03345 to 0.02649	No	ns	>0.9999
NLS CA vs. NES DN	-0.00472	-0.03298 to 0.02354	No	ns	>0.9999
NLS FC vs. NLS DN	-0.01417	-0.04123 to 0.01288	No	ns	0.8326
NLS FC vs. NES WT	-0.004258	-0.03423 to 0.02571	No	ns	>0.9999
NLS FC vs. NES CA	-0.0247	-0.05467 to 0.005277	No	ns	0.2079
NLS FC vs. NES FC	-0.001414	-0.03139 to 0.02856	No	ns	>0.9999
NLS FC vs. NES DN	-0.002652	-0.03091 to 0.02561	No	ns	>0.9999
NLS DN vs. NES WT	0.009916	-0.01893 to 0.03876	No	ns	0.9917
NLS DN vs. NES CA	-0.01052	-0.03936 to 0.01832	No	ns	0.9864
NLS DN vs. NES FC	0.01276	-0.01608 to 0.04160	No	ns	0.9412
NLS DN vs. NES DN	0.01152	-0.01553 to 0.03858	No	ns	0.9551
NES WT vs. NES CA	-0.02044	-0.05203 to 0.01116	No	ns	0.5659
NES WT vs. NES FC	0.002844	-0.02875 to 0.03444	No	ns	>0.9999
NES WT vs. NES DN	0.001606	-0.02837 to 0.03158	No	ns	>0.9999
NES CA vs. NES FC	0.02328	-0.008312 to 0.05487	No	ns	0.3619
NES CA vs. NES DN	0.02204	-0.007929 to 0.05202	No	ns	0.3648
NES FC vs. NES DN	-0.001238	-0.03121 to 0.02873	No	ns	>0.9999

Figure 3F_Integrated nuclear Phalloidin					
**Tukey's multiple comparisons test**	**Mean Diff.**	**95.00% CI of diff.**	**Significant?**	**Summary**	**Adjusted P Value**
unTF vs. CA NLS	-1067	-1534 to -599.7	Yes	****	<0.0001
unTF vs. DN NLS	90.45	-349.3 to 530.2	No	ns	0.949
unTF vs. CA NES	-673.1	-1083 to -263.6	Yes	***	0.0003
CA NLS vs. DN NLS	1157	685.1 to 1629	Yes	****	<0.0001
CA NLS vs. CA NES	393.6	-50.45 to 837.7	No	ns	0.1007
DN NLS vs. CA NES	-763.6	-1179 to -348.3	Yes	****	<0.0001

Figure 3F_Mean nuclear Phalloidin					
**Tukey's multiple comparisons test**	**Mean Diff.**	**95.00% CI of diff.**	**Significant?**	**Summary**	**Adjusted P Value**
unTF vs. CA NLS	-0.03619	-0.04624 to -0.02615	Yes	****	<0.0001
unTF vs. DN NLS	-0.002225	-0.01168 to 0.007231	No	ns	0.9262
unTF vs. CA NES	-0.03021	-0.03902 to -0.02141	Yes	****	<0.0001
CA NLS vs. DN NLS	0.03397	0.02382 to 0.04412	Yes	****	<0.0001
CA NLS vs. CA NES	0.005979	-0.003569 to 0.01553	No	ns	0.3607
DN NLS vs. CA NES	-0.02799	-0.03692 to -0.01906	Yes	****	<0.0001

Figure 4A_Integrated DAPI Intensity					
**Tukey's multiple comparisons test**	**Mean Diff.**	**95.00% CI of diff.**	**Significant?**	**Summary**	**Adjusted P Value**
GFP vs. Notag WT	-0.1554	-0.2934 to -0.01734	Yes	*	0.015
GFP vs. Notag CA	0.0009337	-0.1371 to 0.1390	No	ns	>0.9999
GFP vs. Notag FC	-0.29	-0.4280 to -0.1519	Yes	****	<0.0001
GFP vs. Notag DN	0.03204	-0.1060 to 0.1701	No	ns	0.9998
GFP vs. NLS WT	-0.1433	-0.2962 to 0.009657	No	ns	0.0867
GFP vs. NLS CA	-0.1881	-0.3323 to -0.04391	Yes	**	0.0022
GFP vs. NLS FC	-0.1566	-0.3008 to -0.01239	Yes	*	0.0222
GFP vs. NLS DN	0.07315	-0.06490 to 0.2112	No	ns	0.8208
GFP vs. NES WT	-0.1362	-0.2891 to 0.01678	No	ns	0.1266
GFP vs. NES CA	0.01049	-0.1424 to 0.1634	No	ns	>0.9999
GFP vs. NES FC	-0.07697	-0.2299 to 0.07597	No	ns	0.8657
GFP vs. NES DN	0.1591	0.006128 to 0.3120	Yes	*	0.0345
Notag WT vs. Notag CA	0.1563	0.02470 to 0.2879	Yes	**	0.0079
Notag WT vs. Notag FC	-0.1346	-0.2662 to -0.002960	Yes	*	0.0407
Notag WT vs. Notag DN	0.1874	0.05581 to 0.3191	Yes	***	0.0006
Notag WT vs. NLS WT	0.01211	-0.1351 to 0.1593	No	ns	>0.9999
Notag WT vs. NLS CA	-0.03271	-0.1708 to 0.1053	No	ns	0.9998
Notag WT vs. NLS FC	-0.001187	-0.1392 to 0.1369	No	ns	>0.9999
Notag WT vs. NLS DN	0.2285	0.09692 to 0.3602	Yes	****	<0.0001
Notag WT vs. NES WT	0.01923	-0.1279 to 0.1664	No	ns	>0.9999
Notag WT vs. NES CA	0.1659	0.01872 to 0.3130	Yes	*	0.0147
Notag WT vs. NES FC	0.07842	-0.06874 to 0.2256	No	ns	0.8154
Notag WT vs. NES DN	0.3145	0.1673 to 0.4616	Yes	****	<0.0001
Notag CA vs. Notag FC	-0.2909	-0.4225 to -0.1593	Yes	****	<0.0001
Notag CA vs. Notag DN	0.03111	-0.1005 to 0.1627	No	ns	0.9998
Notag CA vs. NLS WT	-0.1442	-0.2914 to 0.002950	No	ns	0.0598
Notag CA vs. NLS CA	-0.189	-0.3271 to -0.05098	Yes	**	0.0011
Notag CA vs. NLS FC	-0.1575	-0.2956 to -0.01946	Yes	*	0.0128
Notag CA vs. NLS DN	0.07222	-0.05941 to 0.2038	No	ns	0.7852
Notag CA vs. NES WT	-0.1371	-0.2843 to 0.01007	No	ns	0.0907
Notag CA vs. NES CA	0.009558	-0.1376 to 0.1567	No	ns	>0.9999
Notag CA vs. NES FC	-0.0779	-0.2251 to 0.06926	No	ns	0.8219
Notag CA vs. NES DN	0.1581	0.01097 to 0.3053	Yes	*	0.0248
Notag FC vs. Notag DN	0.322	0.1904 to 0.4536	Yes	****	<0.0001
Notag FC vs. NLS WT	0.1467	-0.0004657 to 0.2939	No	ns	0.0514
Notag FC vs. NLS CA	0.1019	-0.03617 to 0.2399	No	ns	0.3587
Notag FC vs. NLS FC	0.1334	-0.004651 to 0.2715	No	ns	0.0674
Notag FC vs. NLS DN	0.3631	0.2315 to 0.4948	Yes	****	<0.0001
Notag FC vs. NES WT	0.1538	0.006656 to 0.3010	Yes	*	0.0329
Notag FC vs. NES CA	0.3005	0.1533 to 0.4476	Yes	****	<0.0001
Notag FC vs. NES FC	0.213	0.06585 to 0.3602	Yes	***	0.0004
Notag FC vs. NES DN	0.449	0.3019 to 0.5962	Yes	****	<0.0001
Notag DN vs. NLS WT	-0.1753	-0.3225 to -0.02816	Yes	**	0.0076
Notag DN vs. NLS CA	-0.2201	-0.3582 to -0.08209	Yes	****	<0.0001

Notag DN vs. NLS FC	-0.1886	-0.3267 to -0.05057	Yes	**	0.0011
Notag DN vs. NLS DN	0.04111	-0.09052 to 0.1727	No	ns	0.9964
Notag DN vs. NES WT	-0.1682	-0.3154 to -0.02104	Yes	*	0.0125
Notag DN vs. NES CA	-0.02155	-0.1687 to 0.1256	No	ns	>0.9999
Notag DN vs. NES FC	-0.109	-0.2562 to 0.03815	No	ns	0.353
Notag DN vs. NES DN	0.127	-0.02014 to 0.2742	No	ns	0.156
NLS WT vs. NLS CA	-0.04482	-0.1978 to 0.1081	No	ns	0.998
NLS WT vs. NLS FC	-0.0133	-0.1662 to 0.1396	No	ns	>0.9999
NLS WT vs. NLS DN	0.2164	0.06927 to 0.3636	Yes	***	0.0003
NLS WT vs. NES WT	0.007122	-0.1541 to 0.1683	No	ns	>0.9999
NLS WT vs. NES CA	0.1538	-0.007438 to 0.3150	No	ns	0.0751
NLS WT vs. NES FC	0.06631	-0.09490 to 0.2275	No	ns	0.9648
NLS WT vs. NES DN	0.3023	0.1411 to 0.4636	Yes	****	<0.0001
NLS CA vs. NLS FC	0.03152	-0.1127 to 0.1757	No	ns	0.9999
NLS CA vs. NLS DN	0.2613	0.1232 to 0.3993	Yes	****	<0.0001
NLS CA vs. NES WT	0.05194	-0.1010 to 0.2049	No	ns	0.9925
NLS CA vs. NES CA	0.1986	0.04565 to 0.3515	Yes	**	0.0024
NLS CA vs. NES FC	0.1111	-0.04180 to 0.2641	No	ns	0.3823
NLS CA vs. NES DN	0.3472	0.1942 to 0.5001	Yes	****	<0.0001
NLS FC vs. NLS DN	0.2297	0.09168 to 0.3678	Yes	****	<0.0001
NLS FC vs. NES WT	0.02042	-0.1325 to 0.1734	No	ns	>0.9999
NLS FC vs. NES CA	0.1671	0.01413 to 0.3200	Yes	*	0.0209
NLS FC vs. NES FC	0.07961	-0.07333 to 0.2325	No	ns	0.8374
NLS FC vs. NES DN	0.3156	0.1627 to 0.4686	Yes	****	<0.0001
NLS DN vs. NES WT	-0.2093	-0.3565 to -0.06215	Yes	***	0.0006
NLS DN vs. NES CA	-0.06266	-0.2098 to 0.08450	No	ns	0.9547
NLS DN vs. NES FC	-0.1501	-0.2973 to -0.002958	Yes	*	0.0416
NLS DN vs. NES DN	0.08591	-0.06125 to 0.2331	No	ns	0.7115
NES WT vs. NES CA	0.1466	-0.01456 to 0.3079	No	ns	0.1085
NES WT vs. NES FC	0.05919	-0.1020 to 0.2204	No	ns	0.9855
NES WT vs. NES DN	0.2952	0.1340 to 0.4564	Yes	****	<0.0001
NES CA vs. NES FC	-0.08746	-0.2487 to 0.07375	No	ns	0.7973
NES CA vs. NES DN	0.1486	-0.01264 to 0.3098	No	ns	0.0985
NES FC vs. NES DN	0.236	0.07482 to 0.3972	Yes	***	0.0004

Figure 4B_Mean DAPI Intensity					
**Tukey's multiple comparisons test**	**Mean Diff.**	**95.00% CI of diff.**	**Significant?**	**Summary**	**Adjusted P Value**
GFP vs. Notag WT	-0.003824	-0.06397 to 0.05632	No	ns	>0.9999
GFP vs. Notag CA	-0.04056	-0.1007 to 0.01958	No	ns	0.4995
GFP vs. Notag FC	-0.01489	-0.07503 to 0.04526	No	ns	0.9996
GFP vs. Notag DN	0.02241	-0.03774 to 0.08255	No	ns	0.9837
GFP vs. NLS WT	0.02402	-0.04261 to 0.09065	No	ns	0.9875
GFP vs. NLS CA	0.01094	-0.05188 to 0.07376	No	ns	>0.9999
GFP vs. NLS FC	0.02509	-0.03773 to 0.08791	No	ns	0.9718
GFP vs. NLS DN	0.02644	-0.03371 to 0.08658	No	ns	0.9433
GFP vs. NES WT	0.01850	-0.04813 to 0.08513	No	ns	0.9988
GFP vs. NES CA	-0.04612	-0.1128 to 0.02051	No	ns	0.4584
GFP vs. NES FC	0.02933	-0.03730 to 0.09596	No	ns	0.9428
GFP vs. NES DN	0.1000	0.03341 to 0.1667	Yes	***	0.0002
Notag WT vs. Notag CA	-0.03674	-0.09409 to 0.02061	No	ns	0.5794
Notag WT vs. Notag FC	-0.01106	-0.06841 to 0.04629	No	ns	>0.9999
Notag WT vs. Notag DN	0.02623	-0.03111 to 0.08358	No	ns	0.9258
Notag WT vs. NLS WT	0.02784	-0.03627 to 0.09196	No	ns	0.9479
Notag WT vs. NLS CA	0.01476	-0.04538 to 0.07491	No	ns	0.9996
Notag WT vs. NLS FC	0.02891	-0.03123 to 0.08906	No	ns	0.8980
Notag WT vs. NLS DN	0.03026	-0.02708 to 0.08761	No	ns	0.8248
Notag WT vs. NES WT	0.02232	-0.04179 to 0.08644	No	ns	0.9907
Notag WT vs. NES CA	-0.04230	-0.1064 to 0.02182	No	ns	0.5342
Notag WT vs. NES FC	0.03315	-0.03096 to 0.09727	No	ns	0.8433
Notag WT vs. NES DN	0.1039	0.03975 to 0.1680	Yes	****	<0.0001
Notag CA vs. Notag FC	0.02568	-0.03167 to 0.08302	No	ns	0.9357
Notag CA vs. Notag DN	0.06297	0.005626 to 0.1203	Yes	*	0.0197
Notag CA vs. NLS WT	0.06458	0.0004664 to 0.1287	Yes	*	0.0468
Notag CA vs. NLS CA	0.05150	-0.008642 to 0.1116	No	ns	0.1642
Notag CA vs. NLS FC	0.06565	0.005507 to 0.1258	Yes	*	0.0210
Notag CA vs. NLS DN	0.06700	0.009656 to 0.1243	Yes	**	0.0097
Notag CA vs. NES WT	0.05906	-0.005052 to 0.1232	No	ns	0.0988
Notag CA vs. NES CA	-0.005559	-0.06968 to 0.05856	No	ns	>0.9999
Notag CA vs. NES FC	0.06989	0.005776 to 0.1340	Yes	*	0.0213
Notag CA vs. NES DN	0.1406	0.07649 to 0.2047	Yes	****	<0.0001
Notag FC vs. Notag DN	0.03729	-0.02005 to 0.09464	No	ns	0.5564
Notag FC vs. NLS WT	0.03890	-0.02521 to 0.1030	No	ns	0.6592
Notag FC vs. NLS CA	0.02583	-0.03432 to 0.08597	No	ns	0.9519
Notag FC vs. NLS FC	0.03998	-0.02017 to 0.1001	No	ns	0.5225
Notag FC vs. NLS DN	0.04133	-0.01602 to 0.09867	No	ns	0.3953
Notag FC vs. NES WT	0.03339	-0.03073 to 0.09750	No	ns	0.8371
Notag FC vs. NES CA	-0.03124	-0.09535 to 0.03288	No	ns	0.8893
Notag FC vs. NES FC	0.04421	-0.01990 to 0.1083	No	ns	0.4644
Notag FC vs. NES DN	0.1149	0.05081 to 0.1790	Yes	****	<0.0001
Notag DN vs. NLS WT	0.001609	-0.06251 to 0.06572	No	ns	>0.9999
Notag DN vs. NLS CA	-0.01147	-0.07162 to 0.04868	No	ns	>0.9999

Notag DN vs. NLS FC	0.002680	-0.05747 to 0.06283	No	ns	>0.9999
Notag DN vs. NLS DN	0.004030	-0.05332 to 0.06138	No	ns	>0.9999
Notag DN vs. NES WT	-0.003910	-0.06803 to 0.06021	No	ns	>0.9999
Notag DN vs. NES CA	-0.06853	-0.1326 to -0.004417	Yes	*	0.0262
Notag DN vs. NES FC	0.006919	-0.05720 to 0.07103	No	ns	>0.9999
Notag DN vs. NES DN	0.07763	0.01352 to 0.1417	Yes	**	0.0062
NLS WT vs. NLS CA	-0.01308	-0.07971 to 0.05355	No	ns	>0.9999
NLS WT vs. NLS FC	0.001071	-0.06556 to 0.06770	No	ns	>0.9999
NLS WT vs. NLS DN	0.002421	-0.06169 to 0.06654	No	ns	>0.9999
NLS WT vs. NES WT	-0.005519	-0.07575 to 0.06472	No	ns	>0.9999
NLS WT vs. NES CA	-0.07014	-0.1404 to 9.366e-00	No	ns	0.0506
NLS WT vs. NES FC	0.005310	-0.06493 to 0.07554	No	ns	>0.9999
NLS WT vs. NES DN	0.07602	0.005787 to 0.1463	Yes	*	0.0230
NLS CA vs. NLS FC	0.01415	-0.04867 to 0.07697	No	ns	0.9999
NLS CA vs. NLS DN	0.01550	-0.04465 to 0.07565	No	ns	0.9994
NLS CA vs. NES WT	0.007560	-0.05907 to 0.07419	No	ns	>0.9999
NLS CA vs. NES CA	-0.05706	-0.1237 to 0.009568	No	ns	0.1641
NLS CA vs. NES FC	0.01839	-0.04824 to 0.08502	No	ns	0.9989
NLS CA vs. NES DN	0.08910	0.02247 to 0.1557	Yes	**	0.0015
NLS FC vs. NLS DN	0.001350	-0.05880 to 0.06150	No	ns	>0.9999
NLS FC vs. NES WT	-0.006590	-0.07322 to 0.06004	No	ns	>0.9999
NLS FC vs. NES CA	-0.07121	-0.1378 to -0.004582	Yes	*	0.0263
NLS FC vs. NES FC	0.004239	-0.06239 to 0.07087	No	ns	>0.9999
NLS FC vs. NES DN	0.07495	0.008320 to 0.1416	Yes	*	0.0151
NLS DN vs. NES WT	-0.007940	-0.07206 to 0.05618	No	ns	>0.9999
NLS DN vs. NES CA	-0.07256	-0.1367 to -0.008447	Yes	*	0.0141
NLS DN vs. NES FC	0.002889	-0.06123 to 0.06700	No	ns	>0.9999
NLS DN vs. NES DN	0.07360	0.009485 to 0.1377	Yes	*	0.0119
NES WT vs. NES CA	-0.06462	-0.1349 to 0.005613	No	ns	0.0997
NES WT vs. NES FC	0.01083	-0.05941 to 0.08106	No	ns	>0.9999
NES WT vs. NES DN	0.08154	0.01131 to 0.1518	Yes	*	0.0104
NES CA vs. NES FC	0.07545	0.005216 to 0.1457	Yes	*	0.0249
NES CA vs. NES DN	0.1462	0.07593 to 0.2164	Yes	****	<0.0001
NES FC vs. NES DN	0.07071	0.0004770 to 0.1409	Yes	*	0.0470

**Figure 4E_DMSO - Nuclear Area**					
**Tukey's multiple comparisons test**	**Mean Diff.**	**95.00% CI of diff.**	**Significant?**	**Summary**	**Adjusted P Value**
Notag DMSO vs. NLS DMSO	-0.1343	-0.2212 to -0.04751	Yes	**	0.0044
Notag DMSO vs. NES DMSO	0.03448	-0.05705 to 0.1260	No	ns	0.5743
NLS DMSO vs. NES DMSO	0.1688	0.08199 to 0.2557	Yes	***	0.0009

**Figure 4F_Y27632 - Nuclear Area**					
**Tukey's multiple comparisons test**	**Mean Diff.**	**95.00% CI of diff.**	**Significant?**	**Summary**	**Adjusted P Value**
Notag Y27632 vs. NLS Y27632	-0.04286	-0.1565 to 0.07078	No	ns	0.5736
Notag Y27632 vs. NES Y27632	-0.0106	-0.1304 to 0.1092	No	ns	0.9682
NLS Y27632 vs. NES Y27632	0.03226	-0.08137 to 0.1459	No	ns	0.7241

**Figure 4G_DMSO - Integrated DAPI**					
**Tukey's multiple comparisons test**	**Mean Diff.**	**95.00% CI of diff.**	**Significant?**	**Summary**	**Adjusted P Value**
Notag DMSO vs. NLS DMSO	-0.1951	-0.3229 to -0.06726	Yes	**	0.0048
Notag DMSO vs. NES DMSO	-0.01773	-0.1524 to 0.1170	No	ns	0.9312
NLS DMSO vs. NES DMSO	0.1773	0.04953 to 0.3051	Yes	**	0.0088

**Figure 4H_Y27632 - Integrated DAPI**					
**Tukey's multiple comparisons test**	**Mean Diff.**	**95.00% CI of diff.**	**Significant?**	**Summary**	**Adjusted P Value**
Notag Y27632 vs. NLS Y27632	0.01786	-0.1212 to 0.1569	No	ns	0.9344
Notag Y27632 vs. NES Y27632	-0.00679	-0.1534 to 0.1398	No	ns	0.9912
NLS Y27632 vs. NES Y27632	-0.02465	-0.1637 to 0.1144	No	ns	0.8795

Figure 5A_Integrated Nuclear pERK					
Tukey's multiple comparisons test	Mean Diff.	95.00% CI of diff.	Significant?	Summary	Adjusted P Value
DMSO vs. Y26732	2.156	1.004 to 3.308	Yes	**	0.0015
DMSO vs. U0126	1.974	0.8226 to 3.126	Yes	**	0.0026
DMSO vs. Y26732+U0126	2.458	1.306 to 3.609	Yes	***	0.0006
Y26732 vs. U0126	-0.1817	-1.333 to 0.9702	No	ns	0.9556
Y26732 vs. Y26732+U0126	0.3014	-0.8504 to 1.453	No	ns	0.8353
U0126 vs. Y26732+U0126	0.4831	-0.6687 to 1.635	No	ns	0.5639

Figure 5B_Nuclear Area					
Tukey's multiple comparisons test	Mean Diff.	95.00% CI of diff.	Significant?	Summary	Adjusted P Value
DMSO vs. Y26732	654.3	201.1 to 1107	Yes	**	0.0074
DMSO vs. U0126	457.9	4.738 to 911.0	Yes	*	0.0477
DMSO vs. Y26732+U0126	754	300.9 to 1207	Yes	**	0.0031
Y26732 vs. U0126	-196.4	-649.5 to 256.8	No	ns	0.5395
Y26732 vs. Y26732+U0126	99.77	-353.4 to 552.9	No	ns	0.8923
U0126 vs. Y26732+U0126	296.2	-157.0 to 749.3	No	ns	0.2339

Figure 5C_Integrated DAPI intensity					
Tukey's multiple comparisons test	Mean Diff.	95.00% CI of diff.	Significant?	Summary	Adjusted P Value
DMSO vs. Y26732	6.907	2.609 to 11.20	Yes	**	0.0039
DMSO vs. U0126	6.514	2.216 to 10.81	Yes	**	0.0055
DMSO vs. Y26732+U0126	7.476	3.179 to 11.77	Yes	**	0.0024
Y26732 vs. U0126	-0.3933	-4.691 to 3.904	No	ns	0.9906
Y26732 vs. Y26732+U0126	0.5696	-3.728 to 4.867	No	ns	0.9727
U0126 vs. Y26732+U0126	0.9629	-3.335 to 5.261	No	ns	0.8875

Figure 5D_Integrated nuclear pERK intensity					
Tukey's multiple comparisons test	Mean Diff.	95.00% CI of diff.	Significant?	Summary	Adjusted P Value
DMSO_Empty Vector vs. DMSO_NLS CA RhoA	-432	-851.3 to -13.45	Yes	*	0.0389
DMSO_Empty Vector vs. DMSO_NES CA RhoA	126.9	-292.0 to 545.9	No	ns	0.9922
DMSO_Empty Vector vs. Y27632_Empty Vector	426.1	7.134 to 845.0	Yes	*	0.0438
DMSO_Empty Vector vs. Y27632_NLS CA RhoA	410.1	-8.801 to 829.1	No	ns	0.0588
DMSO_Empty Vector vs. Y27632_NES CA RhoA	452.5	33.52 to 871.4	Yes	*	0.0265
DMSO_Empty Vector vs. U0126_Empty Vector	375.2	-43.74 to 794.1	No	ns	0.1091
DMSO_Empty Vector vs. U0126_NLS CA RhoA	385.9	-33.00 to 804.9	No	ns	0.0906
DMSO_Empty Vector vs. U0126_NES CA RhoA	490	71.07 to 909.0	Yes	*	0.0126
DMSO_Empty Vector vs. Y27632+U0126_Empty Vector	497.2	78.25 to 916.1	Yes	*	0.0109
DMSO_Empty Vector vs. Y27632+U0126_NLS CA RhoA	651.6	232.7 to 1071	Yes	***	0.0005
DMSO_Empty Vector vs. Y27632+U0126_NES CA RhoA	665.2	246.3 to 1084	Yes	***	0.0003
DMSO_NLS CA RhoA vs. DMSO_NES CA RhoA	559.3	140.4 to 978.3	Yes	**	0.0031
DMSO_NLS CA RhoA vs. Y27632_Empty Vector	858.5	439.5 to 1277	Yes	****	<0.0001
DMSO_NLS CA RhoA vs. Y27632_NLS CA RhoA	842.5	423.6 to 1261	Yes	****	<0.0001
DMSO_NLS CA RhoA vs. Y27632_NES CA RhoA	884.9	465.9 to 1304	Yes	****	<0.0001

DMSO_NLS CA RhoA vs. U0126_Empty Vector	807.6	388.7 to 1227	Yes	****	<0.0001
DMSO_NLS CA RhoA vs. U0126_NLS CA RhoA	818.3	399.4 to 1237	Yes	****	<0.0001
DMSO_NLS CA RhoA vs. U0126_NES CA RhoA	922.4	503.5 to 1341	Yes	****	<0.0001
DMSO_NLS CA RhoA vs. Y27632+U0126_Empty Vector	929.6	510.7 to 1349	Yes	****	<0.0001
DMSO_NLS CA RhoA vs. Y27632+U0126_NLS CA RhoA	1084	665.0 to 1503	Yes	****	<0.0001
DMSO_NLS CA RhoA vs. Y27632+U0126_NES CA RhoA	1098	678.7 to 1517	Yes	****	<0.0001
DMSO_NES CA RhoA vs. Y27632_Empty Vector	299.2	-119.8 to 718.1	No	ns	0.345
DMSO_NES CA RhoA vs. Y27632_NLS CA RhoA	283.2	-135.7 to 702.2	No	ns	0.4201
DMSO_NES CA RhoA vs. Y27632_NES CA RhoA	325.5	-93.40 to 744.5	No	ns	0.2399
DMSO_NES CA RhoA vs. U0126_Empty Vector	248.3	-170.7 to 667.2	No	ns	0.6043
DMSO_NES CA RhoA vs. U0126_NLS CA RhoA	259	-159.9 to 678.0	No	ns	0.5461
DMSO_NES CA RhoA vs. U0126_NES CA RhoA	363.1	-55.85 to 782.0	No	ns	0.1336
DMSO_NES CA RhoA vs. Y27632+U0126_Empty Vector	370.3	-48.66 to 789.2	No	ns	0.1185
DMSO_NES CA RhoA vs. Y27632+U0126_NLS CA RhoA	524.7	105.7 to 943.6	Yes	**	0.0063
DMSO_NES CA RhoA vs. Y27632+U0126_NES CA RhoA	538.3	119.4 to 957.3	Yes	**	0.0047
Y27632_Empty Vector vs. Y27632_NLS CA RhoA	-15.9	-434.9 to 403.0	No	ns	>0.9999
Y27632_Empty Vector vs. Y27632_NES CA RhoA	26.38	-392.6 to 445.3	No	ns	>0.9999
Y27632_Empty Vector vs. U0126_Empty Vector	-50.8	-469.8 to 368.1	No	ns	>0.9999
Y27632_Empty Vector vs. U0126_NLS CA RhoA	-40.1	-459.1 to 378.8	No	ns	>0.9999
Y27632_Empty Vector vs. U0126_NES CA RhoA	63.94	-355.0 to 482.9	No	ns	>0.9999
Y27632_Empty Vector vs. Y27632+U0126_Empty Vector	71.12	-347.8 to 490.1	No	ns	>0.9999
Y27632_Empty Vector vs. Y27632+U0126_NLS CA RhoA	225.5	-193.4 to 644.5	No	ns	0.7247
Y27632_Empty Vector vs. Y27632+U0126_NES CA RhoA	239.2	-179.8 to 658.1	No	ns	0.6534
Y27632_NLS CA RhoA vs. Y27632_NES CA RhoA	42.32	-376.6 to 461.3	No	ns	>0.9999
Y27632_NLS CA RhoA vs. U0126_Empty Vector	-34.9	-453.9 to 384.0	No	ns	>0.9999
Y27632_NLS CA RhoA vs. U0126_NLS CA RhoA	-24.2	-443.1 to 394.7	No	ns	>0.9999
Y27632_NLS CA RhoA vs. U0126_NES CA RhoA	79.87	-339.1 to 498.8	No	ns	0.9999
Y27632_NLS CA RhoA vs. Y27632+U0126_Empty Vector	87.06	-331.9 to 506.0	No	ns	0.9997
Y27632_NLS CA RhoA vs. Y27632+U0126_NLS CA RhoA	241.5	-177.5 to 660.4	No	ns	0.6412
Y27632_NLS CA RhoA vs. Y27632+U0126_NES CA RhoA	255.1	-163.8 to 674.0	No	ns	0.5673
Y27632_NES CA RhoA vs. U0126_Empty Vector	-77.2	-496.2 to 341.7	No	ns	>0.9999
Y27632_NES CA RhoA vs. U0126_NLS CA RhoA	-66.5	-485.5 to 352.4	No	ns	>0.9999
Y27632_NES CA RhoA vs. U0126_NES CA RhoA	37.55	-381.4 to 456.5	No	ns	>0.9999
Y27632_NES CA RhoA vs. Y27632+U0126_Empty Vector	44.74	-374.2 to 463.7	No	ns	>0.9999
Y27632_NES CA RhoA vs. Y27632+U0126_NLS CA RhoA	199.1	-219.8 to 618.1	No	ns	0.8452
Y27632_NES CA RhoA vs. Y27632+U0126_NES CA RhoA	212.8	-206.2 to 631.7	No	ns	0.7865
U0126_Empty Vector vs. U0126_NLS CA RhoA	10.74	-408.2 to 429.7	No	ns	>0.9999
U0126_Empty Vector vs. U0126_NES CA RhoA	114.8	-304.1 to 533.8	No	ns	0.9966
U0126_Empty Vector vs. Y27632+U0126_Empty Vector	122	-296.9 to 540.9	No	ns	0.9943
U0126_Empty Vector vs. Y27632+U0126_NLS CA RhoA	276.4	-142.5 to 695.3	No	ns	0.4545
U0126_Empty Vector vs. Y27632+U0126_NES CA RhoA	290	-128.9 to 709.0	No	ns	0.387
U0126_NLS CA RhoA vs. U0126_NES CA RhoA	104.1	-314.9 to 523.0	No	ns	0.9985
U0126_NLS CA RhoA vs. Y27632+U0126_Empty Vector	111.3	-307.7 to 530.2	No	ns	0.9974
U0126_NLS CA RhoA vs. Y27632+U0126_NLS CA RhoA	265.7	-153.3 to 684.6	No	ns	0.5106
U0126_NLS CA RhoA vs. Y27632+U0126_NES CA RhoA	279.3	-139.6 to 698.2	No	ns	0.4397
U0126_NES CA RhoA vs. Y27632+U0126_Empty Vector	7.183	-411.8 to 426.1	No	ns	>0.9999

U0126_NES CA RhoA vs. Y27632+U0126_NLS CA RhoA	161.6	-257.4 to 580.5	No	ns	0.9544
U0126_NES CA RhoA vs. Y27632+U0126_NES CA RhoA	175.2	-243.7 to 594.2	No	ns	0.9241
Y27632+U0126_Empty Vector vs. Y27632+U0126_NLS CA RhoA	154.4	-264.5 to 573.3	No	ns	0.9664
Y27632+U0126_Empty Vector vs. Y27632+U0126_NES CA RhoA	168	-250.9 to 587.0	No	ns	0.9414
Y27632+U0126_NLS CA RhoA vs. Y27632+U0126_NES CA RhoA	13.64	-405.3 to 432.6	No	ns	>0.9999

**Figure 5E_Nuclear Area**					
**Tukey's multiple comparisons test**	**Mean Diff.**	**95.00% CI of diff.**	**Significant?**	**Summary**	**Adjusted P Value**
DMSO_Empty Vector vs. DMSO_NLS CA RhoA	-4.701	-9.505 to 0.1037	No	ns	0.0591
DMSO_Empty Vector vs. DMSO_NES CA RhoA	0.8699	-3.934 to 5.674	No	ns	>0.9999
DMSO_Empty Vector vs. Y27632_Empty Vector	4.641	-0.1629 to 9.446	No	ns	0.0649
DMSO_Empty Vector vs. Y27632_NLS CA RhoA	3.655	-1.149 to 8.459	No	ns	0.2646
DMSO_Empty Vector vs. Y27632_NES CA RhoA	5.756	0.9515 to 10.56	Yes	**	0.0099
DMSO_Empty Vector vs. U0126_Empty Vector	4.184	-0.6203 to 8.988	No	ns	0.1298
DMSO_Empty Vector vs. U0126_NLS CA RhoA	4.553	-0.2514 to 9.357	No	ns	0.0745
DMSO_Empty Vector vs. U0126_NES CA RhoA	3.881	-0.9231 to 8.686	No	ns	0.1978
DMSO_Empty Vector vs. Y27632+U0126_Empty Vector	5.922	1.117 to 10.73	Yes	**	0.0074
DMSO_Empty Vector vs. Y27632+U0126_NLS CA RhoA	7.566	2.761 to 12.37	Yes	***	0.0004
DMSO_Empty Vector vs. Y27632+U0126_NES CA RhoA	6.491	1.687 to 11.30	Yes	**	0.0027
DMSO_NLS CA RhoA vs. DMSO_NES CA RhoA	5.571	0.7662 to 10.37	Yes	*	0.0137
DMSO_NLS CA RhoA vs. Y27632_Empty Vector	9.342	4.538 to 14.15	Yes	****	<0.0001
DMSO_NLS CA RhoA vs. Y27632_NLS CA RhoA	8.356	3.551 to 13.16	Yes	****	<0.0001
DMSO_NLS CA RhoA vs. Y27632_NES CA RhoA	10.46	5.652 to 15.26	Yes	****	<0.0001
DMSO_NLS CA RhoA vs. U0126_Empty Vector	8.885	4.080 to 13.69	Yes	****	<0.0001
DMSO_NLS CA RhoA vs. U0126_NLS CA RhoA	9.254	4.449 to 14.06	Yes	****	<0.0001
DMSO_NLS CA RhoA vs. U0126_NES CA RhoA	8.582	3.777 to 13.39	Yes	****	<0.0001
DMSO_NLS CA RhoA vs. Y27632+U0126_Empty Vector	10.62	5.818 to 15.43	Yes	****	<0.0001
DMSO_NLS CA RhoA vs. Y27632+U0126_NLS CA RhoA	12.27	7.462 to 17.07	Yes	****	<0.0001
DMSO_NLS CA RhoA vs. Y27632+U0126_NES CA RhoA	11.19	6.387 to 16.00	Yes	****	<0.0001
DMSO_NES CA RhoA vs. Y27632_Empty Vector	3.772	-1.033 to 8.576	No	ns	0.2284
DMSO_NES CA RhoA vs. Y27632_NLS CA RhoA	2.785	-2.019 to 7.589	No	ns	0.6336
DMSO_NES CA RhoA vs. Y27632_NES CA RhoA	4.886	0.08160 to 9.690	Yes	*	0.0438
DMSO_NES CA RhoA vs. U0126_Empty Vector	3.314	-1.490 to 8.118	No	ns	0.392
DMSO_NES CA RhoA vs. U0126_NLS CA RhoA	3.683	-1.121 to 8.487	No	ns	0.2556
DMSO_NES CA RhoA vs. U0126_NES CA RhoA	3.011	-1.793 to 7.816	No	ns	0.5269
DMSO_NES CA RhoA vs. Y27632+U0126_Empty Vector	5.052	0.2474 to 9.856	Yes	*	0.0333
DMSO_NES CA RhoA vs. Y27632+U0126_NLS CA RhoA	6.696	1.891 to 11.50	Yes	**	0.0018
DMSO_NES CA RhoA vs. Y27632+U0126_NES CA RhoA	5.621	0.8167 to 10.43	Yes	*	0.0126
Y27632_Empty Vector vs. Y27632_NLS CA RhoA	-0.9864	-5.791 to 3.818	No	ns	0.9997
Y27632_Empty Vector vs. Y27632_NES CA RhoA	1.114	-3.690 to 5.919	No	ns	0.9992
Y27632_Empty Vector vs. U0126_Empty Vector	-0.4575	-5.262 to 4.347	No	ns	>0.9999
Y27632_Empty Vector vs. U0126_NLS CA RhoA	-0.08856	-4.893 to 4.716	No	ns	>0.9999
Y27632_Empty Vector vs. U0126_NES CA RhoA	-0.7603	-5.565 to 4.044	No	ns	>0.9999
Y27632_Empty Vector vs. Y27632+U0126_Empty Vector	1.28	-3.524 to 6.085	No	ns	0.9973

Y27632_Empty Vector vs. Y27632+U0126_NLS CA RhoA	2.924	-1.880 to 7.728	No	ns	0.5679
Y27632_Empty Vector vs. Y27632+U0126_NES CA RhoA	1.85	-2.955 to 6.654	No	ns	0.955
Y27632_NLS CA RhoA vs. Y27632_NES CA RhoA	2.101	-2.704 to 6.905	No	ns	0.9014
Y27632_NLS CA RhoA vs. U0126_Empty Vector	0.529	-4.275 to 5.333	No	ns	>0.9999
Y27632_NLS CA RhoA vs. U0126_NLS CA RhoA	0.8979	-3.906 to 5.702	No	ns	0.9999
Y27632_NLS CA RhoA vs. U0126_NES CA RhoA	0.2262	-4.578 to 5.031	No	ns	>0.9999
Y27632_NLS CA RhoA vs. Y27632+U0126_Empty Vector	2.267	-2.538 to 7.071	No	ns	0.851
Y27632_NLS CA RhoA vs. Y27632+U0126_NLS CA RhoA	3.911	-0.8938 to 8.715	No	ns	0.1902
Y27632_NLS CA RhoA vs. Y27632+U0126_NES CA RhoA	2.836	-1.968 to 7.640	No	ns	0.6096
Y27632_NES CA RhoA vs. U0126_Empty Vector	-1.572	-6.376 to 3.232	No	ns	0.9858
Y27632_NES CA RhoA vs. U0126_NLS CA RhoA	-1.203	-6.007 to 3.601	No	ns	0.9984
Y27632_NES CA RhoA vs. U0126_NES CA RhoA	-1.875	-6.679 to 2.930	No	ns	0.9508
Y27632_NES CA RhoA vs. Y27632+U0126_Empty Vector	0.1658	-4.638 to 4.970	No	ns	>0.9999
Y27632_NES CA RhoA vs. Y27632+U0126_NLS CA RhoA	1.81	-2.995 to 6.614	No	ns	0.9611
Y27632_NES CA RhoA vs. Y27632+U0126_NES CA RhoA	0.7351	-4.069 to 5.539	No	ns	>0.9999
U0126_Empty Vector vs. U0126_NLS CA RhoA	0.3689	-4.435 to 5.173	No	ns	>0.9999
U0126_Empty Vector vs. U0126_NES CA RhoA	-0.3028	-5.107 to 4.502	No	ns	>0.9999
U0126_Empty Vector vs. Y27632+U0126_Empty Vector	1.738	-3.067 to 6.542	No	ns	0.9705
U0126_Empty Vector vs. Y27632+U0126_NLS CA RhoA	3.382	-1.423 to 8.186	No	ns	0.3644
U0126_Empty Vector vs. Y27632+U0126_NES CA RhoA	2.307	-2.497 to 7.111	No	ns	0.837
U0126_NLS CA RhoA vs. U0126_NES CA RhoA	-0.6717	-5.476 to 4.133	No	ns	>0.9999
U0126_NLS CA RhoA vs. Y27632+U0126_Empty Vector	1.369	-3.436 to 6.173	No	ns	0.9952
U0126_NLS CA RhoA vs. Y27632+U0126_NLS CA RhoA	3.013	-1.792 to 7.817	No	ns	0.5263
U0126_NLS CA RhoA vs. Y27632+U0126_NES CA RhoA	1.938	-2.866 to 6.742	No	ns	0.9392
U0126_NES CA RhoA vs. Y27632+U0126_Empty Vector	2.041	-2.764 to 6.845	No	ns	0.9168
U0126_NES CA RhoA vs. Y27632+U0126_NLS CA RhoA	3.684	-1.120 to 8.489	No	ns	0.2552
U0126_NES CA RhoA vs. Y27632+U0126_NES CA RhoA	2.61	-2.195 to 7.414	No	ns	0.7142
Y27632+U0126_Empty Vector vs. Y27632+U0126_NLS CA RhoA	1.644	-3.160 to 6.448	No	ns	0.9802
Y27632+U0126_Empty Vector vs. Y27632+U0126_NES CA RhoA	0.5693	-4.235 to 5.374	No	ns	>0.9999
Y27632+U0126_NLS CA RhoA vs. Y27632+U0126_NES CA RhoA	-1.075	-5.879 to 3.730	No	ns	0.9994

**Figure 5F_Integrated DAPI intensity**					
**Tukey's multiple comparisons test**	**Mean Diff.**	**95.00% CI of diff.**	**Significant?**	**Summary**	**Adjusted P Value**
DMSO_Empty Vector vs. DMSO_NLS CA RhoA	-2.715	-5.423 to -0.007461	Yes	*	0.0489
DMSO_Empty Vector vs. DMSO_NES CA RhoA	0.8079	-1.900 to 3.515	No	ns	0.9931
DMSO_Empty Vector vs. Y27632_Empty Vector	3.073	0.3652 to 5.780	Yes	*	0.0169
DMSO_Empty Vector vs. Y27632_NLS CA RhoA	1.96	-0.7480 to 4.667	No	ns	0.3273
DMSO_Empty Vector vs. Y27632_NES CA RhoA	3.535	0.8278 to 6.243	Yes	**	0.004
DMSO_Empty Vector vs. U0126_Empty Vector	3.399	0.6909 to 6.106	Yes	**	0.0061
DMSO_Empty Vector vs. U0126_NLS CA RhoA	3.159	0.4509 to 5.866	Yes	*	0.013
DMSO_Empty Vector vs. U0126_NES CA RhoA	3.531	0.8238 to 6.239	Yes	**	0.004
DMSO_Empty Vector vs. Y27632+U0126_Empty Vector	3.904	1.196 to 6.611	Yes	**	0.0012
DMSO_Empty Vector vs. Y27632+U0126_NLS CA RhoA	4.133	1.425 to 6.841	Yes	***	0.0006
DMSO_Empty Vector vs. Y27632+U0126_NES CA RhoA	3.306	0.5988 to 6.014	Yes	**	0.0082
DMSO_NLS CA RhoA vs. DMSO_NES CA RhoA	3.523	0.8153 to 6.231	Yes	**	0.0041

DMSO_NLS CA RhoA vs. Y27632_Empty Vector	5.788	3.080 to 8.495	Yes	****	<0.0001
DMSO_NLS CA RhoA vs. Y27632_NLS CA RhoA	4.675	1.967 to 7.382	Yes	***	0.0001
DMSO_NLS CA RhoA vs. Y27632_NES CA RhoA	6.251	3.543 to 8.958	Yes	****	<0.0001
DMSO_NLS CA RhoA vs. U0126_Empty Vector	6.114	3.406 to 8.821	Yes	****	<0.0001
DMSO_NLS CA RhoA vs. U0126_NLS CA RhoA	5.874	3.166 to 8.581	Yes	****	<0.0001
DMSO_NLS CA RhoA vs. U0126_NES CA RhoA	6.246	3.539 to 8.954	Yes	****	<0.0001
DMSO_NLS CA RhoA vs. Y27632+U0126_Empty Vector	6.619	3.911 to 9.326	Yes	****	<0.0001
DMSO_NLS CA RhoA vs. Y27632+U0126_NLS CA RhoA	6.848	4.140 to 9.556	Yes	****	<0.0001
DMSO_NLS CA RhoA vs. Y27632+U0126_NES CA RhoA	6.021	3.314 to 8.729	Yes	****	<0.0001
DMSO_NES CA RhoA vs. Y27632_Empty Vector	2.265	-0.4427 to 4.973	No	ns	0.164
DMSO_NES CA RhoA vs. Y27632_NLS CA RhoA	1.152	-1.556 to 3.859	No	ns	0.916
DMSO_NES CA RhoA vs. Y27632_NES CA RhoA	2.728	0.01996 to 5.435	Yes	*	0.0472
DMSO_NES CA RhoA vs. U0126_Empty Vector	2.591	-0.1170 to 5.298	No	ns	0.0696
DMSO_NES CA RhoA vs. U0126_NLS CA RhoA	2.351	-0.3569 to 5.058	No	ns	0.1322
DMSO_NES CA RhoA vs. U0126_NES CA RhoA	2.723	0.01588 to 5.431	Yes	*	0.0478
DMSO_NES CA RhoA vs. Y27632+U0126_Empty Vector	3.096	0.3881 to 5.803	Yes	*	0.0157
DMSO_NES CA RhoA vs. Y27632+U0126_NLS CA RhoA	3.325	0.6175 to 6.033	Yes	**	0.0077
DMSO_NES CA RhoA vs. Y27632+U0126_NES CA RhoA	2.499	-0.2091 to 5.206	No	ns	0.0896
Y27632_Empty Vector vs. Y27632_NLS CA RhoA	-1.113	-3.821 to 1.594	No	ns	0.9316
Y27632_Empty Vector vs. Y27632_NES CA RhoA	0.4626	-2.245 to 3.170	No	ns	>0.9999
Y27632_Empty Vector vs. U0126_Empty Vector	0.3257	-2.382 to 3.033	No	ns	>0.9999
Y27632_Empty Vector vs. U0126_NLS CA RhoA	0.08575	-2.622 to 2.793	No	ns	>0.9999
Y27632_Empty Vector vs. U0126_NES CA RhoA	0.4586	-2.249 to 3.166	No	ns	>0.9999
Y27632_Empty Vector vs. Y27632+U0126_Empty Vector	0.8308	-1.877 to 3.538	No	ns	0.9913
Y27632_Empty Vector vs. Y27632+U0126_NLS CA RhoA	1.06	-1.647 to 3.768	No	ns	0.9497
Y27632_Empty Vector vs. Y27632+U0126_NES CA RhoA	0.2336	-2.474 to 2.941	No	ns	>0.9999
Y27632_NLS CA RhoA vs. Y27632_NES CA RhoA	1.576	-1.132 to 4.283	No	ns	0.6283
Y27632_NLS CA RhoA vs. U0126_Empty Vector	1.439	-1.269 to 4.147	No	ns	0.7391
Y27632_NLS CA RhoA vs. U0126_NLS CA RhoA	1.199	-1.509 to 3.907	No	ns	0.8941
Y27632_NLS CA RhoA vs. U0126_NES CA RhoA	1.572	-1.136 to 4.279	No	ns	0.6317
Y27632_NLS CA RhoA vs. Y27632+U0126_Empty Vector	1.944	-0.7636 to 4.652	No	ns	0.3378
Y27632_NLS CA RhoA vs. Y27632+U0126_NLS CA RhoA	2.173	-0.5342 to 4.881	No	ns	0.2044
Y27632_NLS CA RhoA vs. Y27632+U0126_NES CA RhoA	1.347	-1.361 to 4.054	No	ns	0.8063
Y27632_NES CA RhoA vs. U0126_Empty Vector	-0.1369	-2.845 to 2.571	No	ns	>0.9999
Y27632_NES CA RhoA vs. U0126_NLS CA RhoA	-0.3769	-3.085 to 2.331	No	ns	>0.9999
Y27632_NES CA RhoA vs. U0126_NES CA RhoA	-0.0040	-2.712 to 2.704	No	ns	>0.9999
Y27632_NES CA RhoA vs. Y27632+U0126_Empty Vector	0.3681	-2.340 to 3.076	No	ns	>0.9999
Y27632_NES CA RhoA vs. Y27632+U0126_NLS CA RhoA	0.5976	-2.110 to 3.305	No	ns	0.9995
Y27632_NES CA RhoA vs. Y27632+U0126_NES CA RhoA	-0.229	-2.937 to 2.479	No	ns	>0.9999
U0126_Empty Vector vs. U0126_NLS CA RhoA	-0.24	-2.948 to 2.468	No	ns	>0.9999
U0126_Empty Vector vs. U0126_NES CA RhoA	0.1329	-2.575 to 2.840	No	ns	>0.9999
U0126_Empty Vector vs. Y27632+U0126_Empty Vector	0.5051	-2.203 to 3.213	No	ns	0.9999
U0126_Empty Vector vs. Y27632+U0126_NLS CA RhoA	0.7345	-1.973 to 3.442	No	ns	0.9968
U0126_Empty Vector vs. Y27632+U0126_NES CA RhoA	-0.0921	-2.800 to 2.616	No	ns	>0.9999
U0126_NLS CA RhoA vs. U0126_NES CA RhoA	0.3728	-2.335 to 3.080	No	ns	>0.9999

U0126_NLS CA RhoA vs. Y27632+U0126_Empty Vector	0.745	-1.963 to 3.453	No	ns	0.9964
U0126_NLS CA RhoA vs. Y27632+U0126_NLS CA RhoA	0.9745	-1.733 to 3.682	No	ns	0.9715
U0126_NLS CA RhoA vs. Y27632+U0126_NES CA RhoA	0.1479	-2.560 to 2.855	No	ns	>0.9999
U0126_NES CA RhoA vs. Y27632+U0126_Empty Vector	0.3722	-2.335 to 3.080	No	ns	>0.9999
U0126_NES CA RhoA vs. Y27632+U0126_NLS CA RhoA	0.6017	-2.106 to 3.309	No	ns	0.9995
U0126_NES CA RhoA vs. Y27632+U0126_NES CA RhoA	-0.225	-2.933 to 2.483	No	ns	>0.9999
Y27632+U0126_Empty Vector vs. Y27632+U0126_NLS CA RhoA	0.2295	-2.478 to 2.937	No	ns	>0.9999
Y27632+U0126_Empty Vector vs. Y27632+U0126_NES CA RhoA	-0.5972	-3.305 to 2.110	No	ns	0.9995
Y27632+U0126_NLS CA RhoA vs. Y27632+U0126_NES CA RhoA	-0.8266	-3.534 to 1.881	No	ns	0.9917
